# YTHDC2 suppresses bladder cancer by inhibiting SOX2-mediated tumor plasticity

**DOI:** 10.1038/s41419-025-08079-w

**Published:** 2025-10-27

**Authors:** Yi Cai, Cong Zhu, Ming-Hui Shi, Jin-Hui Zhang, Shu-Yan Liu, Jin-Long Cui, Meng-Meng Guo, Dao-Jing Ming, Xian-Tao Zeng, Shuai Yuan, Hong Weng

**Affiliations:** 1 Center for Evidence-Based and Translational Medicine, Zhongnan Hospital of Wuhan University, Wuhan, China; 2 Department of Urology, Zhongnan Hospital of Wuhan University, Wuhan, China; 3 Laboratory of Molecular and Cellular Biology, Institute of Metabolism and Health, School of Basic Medical Sciences, Department of General Surgery of Huaihe Hospital, Henan University, Kaifeng, Henan China; 4 Zhongzhou Laboratory, Kaifeng, China; 5 Department of Urology, Shanghai Pudong New Area Gongli Hospital, Shanghai, China

**Keywords:** Tumour-suppressor proteins, Cancer stem cells, Bladder cancer

## Abstract

Pluripotent cancer stem cells play a pivotal role in inducing phenotypic plasticity across various cancer types, including bladder cancer. This plasticity, crucial for cancer progression, is largely regulated by epigenetic modifications including N6-methyladenosine (m^6^A) in RNAs. However, the role of the m^6^A reader protein YTHDC2 in this process remains poorly understood. In this study, we uncovered that the depletion of YTHDC2 significantly increased the pool of bladder cancer stem cells (BCSCs), resulting in a phenotypic shift towards a more invasive subtype of bladder cancer. This shift was characterized by enhanced proliferation, migration, invasion, and self-renewal capabilities of cancer cells, highlighting YTHDC2’s function as a tumor suppressor. Mechanistically, YTHDC2 recognized and bound to m^6^A-modified *SOX2* mRNA, resulting in translational inhibition of SOX2. In conclusion, our study identifies YTHDC2 as a tumor suppressor in bladder cancer through inhibiting SOX2-mediated cell pluripotency and underscores the therapeutic potential of targeting the YTHDC2-SOX2 axis in bladder cancer.

## Introduction

Bladder cancer (BCa) ranks as the tenth most common cancer worldwide, with 573,278 new cases and 212,536 deaths reported in 2020 [1, 2]. One of the most concerning aspects of BCa is its high recurrence rate [2]. For a long time, researchers have been dedicated to unraveling the intricate mechanisms behind these recurrences and metastases. Substantial evidence suggests the involvement of cancer stem cells (CSCs), a small subpopulation of cancer cells [3–5]. CSCs have self-renewal abilities like normal epithelial stem cells, but differentiate into cancer cells. As tumor-initiating cells (TICs), they drive tumorigenesis, metastasis, drug resistance, and contribute to frequent recurrences under extreme conditions [6–8].

Four key transcription factors—NANOG, OCT4, SOX2 and MYC—are instrumental in regulating the pluripotency of stem cells [9, 10]. SOX2, in particular, plays a critical role in transcription regulation and chromatin architecture [11]. As a member of HMG-domain family (High Mobility Group domain family) of DNA-binding proteins, SOX2 is initially identified for the ability to reprogram somatic cells into induced pluripotent stem cells (iPSC) and maintain the pluripotency by regulating the transcription of targeted genes through its central HMG domains [12]. Interestingly, low expression of SOX2 in the endoderm and mesoderm (precursors to the urinary bladder) is crucial for proper lineage specification [13, 14]. While in tumorigenesis, SOX2 acts as an oncogene [15, 16]. Recent single-cell RNA sequencing studies have identified a positive correlation between higher SOX2 expression and tumor progression [17, 18]. In N-butyl-N-(4-hydroxybutyl) nitrosamine (BBN)-induced murine bladder cancer model, a subpopulation of bladder cancer cells with high SOX2 levels displays stem cell-like features, including self-renewal and enhanced lineage plasticity [17]. SOX2 plays multifaceted regulatory roles in the tumorigenesis across solid tumors; however, the underlying mechanisms cannot be fully explained through canonical mutation-based paradigms alone[19–21].

*N*^*6*^-methyladenosine (m^6^A), the most prevalent reversible methyl modification of adenosine in RNA, plays a crucial role in post-transcriptional regulation, mRNA splicing, RNA export, RNA decay, and translation [22, 23]. The function of m^6^A modification relies on m^6^A readers. YTH domain-containing 2 (YTHDC2), a newly identified m^6^A reader, has been shown to activate spermatogenesis and germline differentiation [24, 25]. In neuronal lineage differentiation of hESCs, YTHDC2 can conversely promote endodermal/mesodermal specification [26]. The role and mechanism of YTHDC2 in regulating cancer stem cells remain unclear.

In this study, we identified YTHDC2 as a tumor suppressor in bladder cancer. Specifically, we found that YTHDC2 can regulate the characteristics of bladder cancer stem cells by recognizing m^6^A-modified SOX2.

## Results

### YTHDC2 is down-regulated in bladder cancer and its reduced expression is associated with poor prognosis

**Fig. 1:**
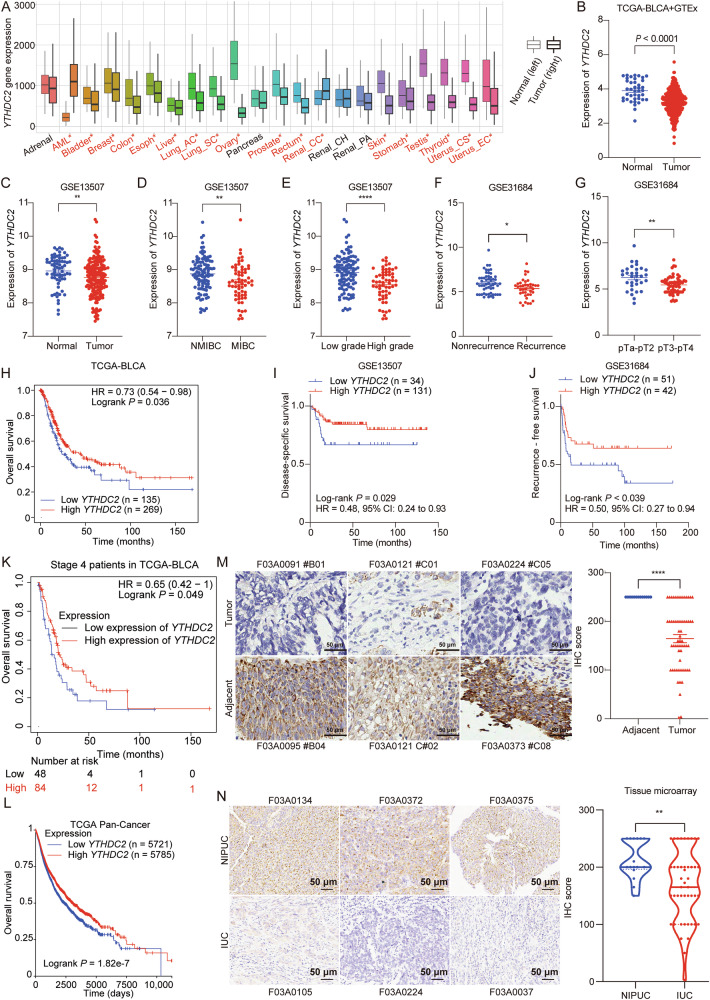
YTHDC2 is downregulated in BLCA and correlates with better survival. **A** The mRNA expression level of *YTHDC2* in normal and tumor tissues. The plot was generated from TNMplot [60] (https://tnmplot.com/analysis/). Significant differences by Mann–Whitney U test were marked with red*. **B** The mRNA expression level of *YTHDC2* in normal (*n* = 40) and BLCA (*n* = 406) tissues from GTEx and TCGA-BLCA datasets. **C** The mRNA expression level of *YTHDC2* in normal (*n* = 68) and BLCA (*n* = 188) tissues from GSE13507 dataset. **D** Expression of *YTHDC2* mRNA in NMIBC (*n* = 103) and MIBC (*n* = 62) from GSE13507 dataset. **E** Expression of *YTHDC2* mRNA in low grade (*n* = 105) and high grade (*n* = 60) BLCA from GSE13507 dataset. **F** Expression of *YTHDC2* mRNA in nonrecurrence (*n* = 54) and recurrence (*n* = 39) BLCA from GSE31684 dataset. **G** Expression of *YTHDC2* mRNA in pTa-pT2 (*n* = 32) and pT3-pT4 (*n* = 61) BLCA from GSE31684 dataset. **H** Overall survival analysis in BLCA patients grouped by *YTHDC2* expression in TCGA-BLCA dataset. **I** Differences of disease-specific survival in BLCA patients grouped by *YTHDC2* expression (high vs. low: *n* = 131vs *n* = 34) in GSE13507 dataset. **J** Recurrence-free survival in BLCA patients based on *YTHDC2* expression in GSE31684 dataset. **K** Kaplan–Meier analysis (KM analysis) was utilized to compare the difference of overall survival rate between two groups of BLCA patients in stage 4. Patients were stratified by expression level of *YTHDC2*. HR value and Log-rank *P* value were shown on the graph. Gene expression and overall survival data came from the public database TCGA-BLCA. **L** Overall survival analysis in pan-cancer based on *YTHDC2* expression in TCGA Pan-Cancer dataset. **M** Representative IHC staining of YTHDC2 expression in adjacent (*n* = 13) and tumor (*n* = 60) tissue from tissue microarrays of bladder cancer patients. Scatter dot plot analysis of YTHDC2 expression from the tissue microarrays are shown on the right. Scale bar: 50 μm. **N** Representative images of YTHDC2 IHC staining in tissues from different bladder cancer subtypes (left), and the quantification of the staining by IHC score. Scale bar: 50 μm. Data were analyzed with Mann–Whitney U test. NIPUC non-invasive papillary urothelial carcinoma, IUC invasive urothelial carcinoma. *P < 0.05, **P < 0.01, ***P < 0.001, ****P < 0.0001.

To compare *YTHDC2* mRNA expression levels between normal and tumor tissues, we analyzed RNA-seq data from 56,938 unique samples in the TNMplot database, comprising 15,648 normal, 40,442 tumor, and 848 metastasis samples. Our analysis revealed a significant difference in *YTHDC2* expression between normal and tumor tissues across 18 of 22 tissue types examined, including bladder. Notably, 88.9% (16/18) of these tumor types exhibited reduced *YTHDC2* levels in tumor tissues (Fig. 1A), suggesting a negative correlation between *YTHDC2* expression and tumorigenesis. Analysis of RNA-seq data from TCGA-BLCA, GTEx, and GSE13507 cohorts also revealed significantly lower *YTHDC2* expression in bladder tumor tissues compared to normal tissues (Fig. 1B, C). *YTHDC2* expression was significantly lower in aggressive tumor subtypes (MIBC, high-grade, recurrent cases, and T3–T4) compared to less advanced disease (Fig. 1D–G).

For survival analysis in BCa, lower *YTHDC2* expression was significantly associated with lower survival rates, including overall, disease-specific and recurrence-free survival (Fig. 1H–J and Supplementary Fig. S1A). Among patients with advanced BCa, particularly stage 4, *YTHDC2* expression also positively correlated with patients’ overall survival rate (Fig. 1K). Aggressive BCa is often characterized by higher tumor mutation burden (TMB) [27]; the somatic mutation rate of invasive BCa subtype, MIBC, is about twofold higher than that of non-invasive subtype, NMIBC [28–30]. In high-TMB Bladder Urothelial Carcinoma (BLCA) samples, lower *YTHDC2*’s expression level was also significantly linked to lower overall survival rate (Supplementary Fig. S1B). Classic tumor suppressor genes (TSGs), *PTEN*, *TP53*, and *RB1*, are frequently altered across malignancies [31–33]. Their expressions were also positively correlated with *YTHDC2* expression in BCa cohorts from TCGA and GEO databases (Supplementary Fig. S2A, B). In other cancer types and pan-cancer cohorts from TCGA, higher *YTHDC2* expression also correlates with higher overall survival rates (Supplementary Fig. S1C and Fig. 1L).

To validate the RNA-seq based bioinformatic analysis, we performed immunohistochemical (IHC) staining on tissue microarrays from bladder cancer patients, including 60 tumor tissues and 13 adjacent normal tissues. IHC results confirmed that YTHDC2 protein level was significantly lower in tumor tissues than in normal tissues (Fig. 1M). YTHDC2 protein level was significantly elevated in non-invasive papillary urothelial carcinoma (NIPUC) compared to invasive urothelial carcinoma (IUC) (Fig. 1N), as well as in early-stage tumors (Tis/T1) compared to advanced-stage tumors (T2) (Supplementary Fig. S1D). Collectively, these findings suggest that YTHDC2 potentially functions as a tumor suppressor in bladder cancer, where its downregulation correlates with aggressive clinicopathological features.

### YTHDC2 inhibits the cell proliferation and epithelial-mesenchymal transition (EMT)-driven plasticity in vitro, as well as tumor growth in vivo

We next explored the potential biological functions of YTHDC2 in bladder cancer. Pearson correlation analysis using TCGA-BLCA data identified 1367 genes positively correlated with *YTHDC2* and 765 negatively correlated genes (Supplementary Fig. S3A). Functional enrichment showed that these YTHDC2-associated genes were significantly enriched in RNA metabolism, protein processing, and DNA repair (Supplementary Fig. S3B–D). Intersection analysis of YTHDC2-associated genes with the Catalogue of Cancer Genes (CCG) revealed several cancer-related genes, such as *DMXL1*, *CHD1*, and *APC*, showing strong positive association with *YTHDC2* expression (Supplementary Fig. S3E). GO enrichment showed these genes were primarily involved in protein modification and phosphorylation processes (Supplementary Fig. S3F). KEGG analysis indicated enrichment in multiple cancer-related pathways, including MAPK signaling, proteoglycans in cancer, and endocrine resistance (Supplementary Fig. S3G). Hallmark analysis further highlighted associations with cell cycle regulation, apoptosis, PI3K-AKT-mTOR signaling, and inflammatory pathways (Supplementary Fig. S3H). Among the YTHDC2 associated and cancer-related genes (CRGs), CHD1 regulates WNT signaling, EMT and pluripotency genes like *SOX2* and *NANOG* [34]. Interestingly, YTHDC2 associated CRGs showed significant enrichment in WNT/β-catenin pathway (Supplementary Fig. S3H), a pivotal and classic pathway inducing cancer cell stemness and promoting EMT in cancer [35–37].

**Fig. 2:**
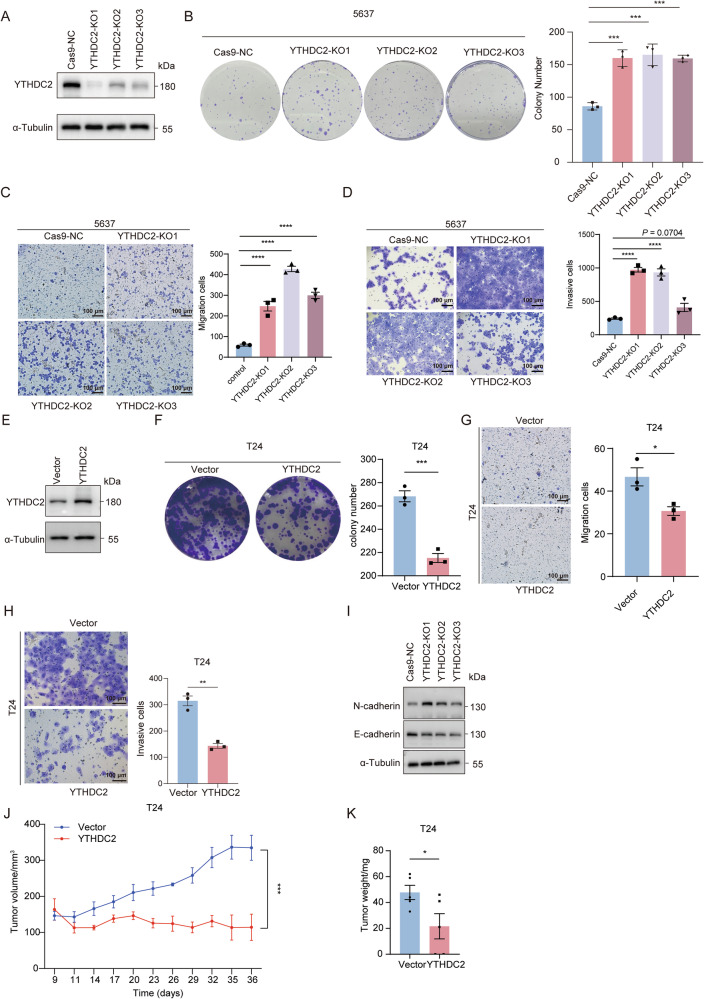
YTHDC2 acted as a tumor suppressor in BLCA by inhibiting cell proliferation and EMT plasticity. **A** Western blotting verification of YTHDC2 protein level in 5637 cells infected with Cas9-NC or YTHDC2 knockout lentivirus. Protein levels were normalized to α-Tubulin levels. **B** Representative images for colony formation results of Cas9-NC and YTHDC2-KO groups of 5637 cells. Graph of quantification of colony numbers is on the right. **C** Representative images of transwell migration assay results and quantification for migrating cells of Cas9-NC and YTHDC2-KO groups of 5637 cells. Scale bar: 100 µm. **D** Representative images of invasion assays and quantification for invading cells of Cas9-NC and YTHDC2-KO groups of 5637 cells. Upper bottom surface of transwell inserts was coated with Matrigel. Scale bar: 100 µm. For the transwell invasion assay, 8 × 10⁴ cells per well were seeded into the upper chambers pre-coated with Matrigel. A higher cell number was used compared to migration assays (4 × 10⁴ cells per well), as the Matrigel coating significantly increases the physical barrier against cell movement. **E** Western blotting verification for YTHDC2 protein level in T24 cells infected with empty-vector (Vector) or YTHDC2 overexpression lentivirus (YTHDC2). Protein levels were normalized to α-Tubulin levels. **F** The colony formation assays were conducted in T24 cells transfected with control or YTHDC2 overexpression vectors. The graph on the right shows the quantification of colony numbers in each group (*n* = 3). **G** Representative images of migration assay results and quantification for migrating cells of T24 transfected with control or YTHDC2 overexpression vectors. Scale bar: 100 µm. **H** Representative images of invasion assays and quantification for invading cells of T24 transfected with control or YTHDC2 overexpression vectors. Upper bottom surface of transwell inserts was coated with Matrigel. Scale bar: 100 µm. For the Transwell invasion assay, 8 × 10⁴ cells per well were seeded into the upper chambers pre-coated with Matrigel. A higher cell number was used compared to migration assays (4 × 10⁴ cells per well), as the Matrigel coating significantly increases the physical barrier to cell movement. **I** Protein abundance visualization of E-cadherin and N-cadherin by Western Blotting, protein levels were normalized to α-Tubulin levels. **J** Tumor volumes of nude mice were monitored for 35 days following subcutaneous injection of T24 cells stably expressing either control vector or YTHDC2 overexpression construct, data were analyzed by repeated measures analysis of variance (ANOVA) to compare the difference of tumor growth rate between vector and YTHDC2 groups. **K** Graph for weight of tumor dissected subcutaneously from nude mice. *P < 0.05, **P < 0.01, ***P < 0.001, ****P  < 0.0001.

To verify the biological function of YTHDC2 in bladder cancer, we firstly detected the endogenous YTHDC2 level in 5637, T24, UM-UC-3, J82 (Supplementary Fig. S4A). Cells with lowest and highest endogenous YTHDC2 level were selected respectively to construct gain- and loss-of-function cell models—a widely adopted strategy in cancer research [38–42]. On the basis of this strategy, YTHDC2 deletion in 5637 cell line significantly promoted cell proliferation, as demonstrated by colony formation assays (Fig. 2A, B) and CCK-8 assays (Supplementary Fig. S4B). Meanwhile, transwell migration and invasion assays identified enhanced capability of cell migration and invasion after YTHDC2 deletion (Fig. 2C, D). In contrast, with the overexpression of YTHDC2 (Fig. 2E), the cell proliferation, migration, and invasion of T24 were significantly attenuated (Fig. 2F–H). We next evaluated the protein levels of EMT plasticity markers. The deletion of YTHDC2 in 5637 elevated the level of N-cadherin, yet reduced that of E-cadherin (Fig. 2I). These molecular changes confirmed the epithelial-to-mesenchymal transition.

We further detected the effect of YTHDC2 on tumor growth using subcutaneous xenograft models in nude mice. Overexpression of YTHDC2 did restrict the growth of T24 tumors in mice (Fig. 2J, K; Supplementary Fig. S4C, D). In footpad xenograft model, nude mice injected with YTHDC2-overexpressed T24 also showed significantly slower tumor growth than control group (Supplementary Fig. S4E, F). IHC staining showed decreased Ki67 level in footpad tumor tissue with YTHDC2 overexpression (Supplementary Fig. S4G). Collectively, these results supported that YTHDC2 acts as a tumor suppressor in bladder cancer.

### YTHDC2 modulates the stemness of bladder cancer cells

**Fig. 3:**
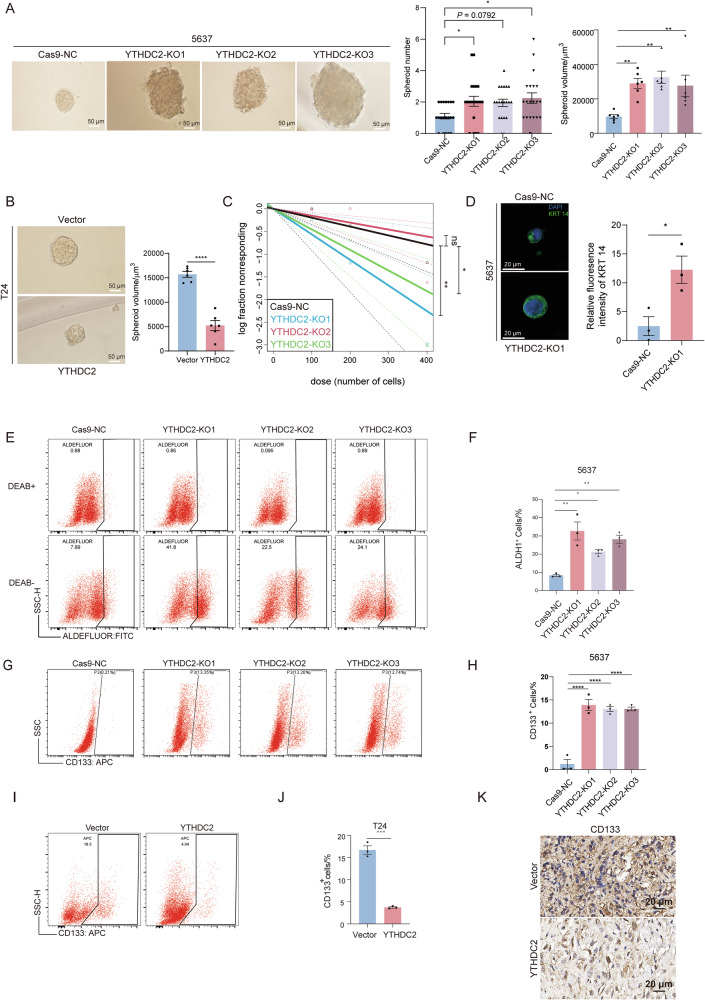
YTHDC2 inhibits BLCA’s self-renewal and cell stemness. **A** Tumor sphere formation assays to evaluate the self-renewal ability of Cas9-NC and YTHDC2-KO1-3 5637 cells. Representative phase contrast microscope images were used to show the morphology of spheroids. The spheroid numbers of each replicate and volume of each spheroid per 250 cells initially seeded was calculated. Quantification results were presented both on the right. Scale bar: 50 µm. **B** Representative phase contrast microscope images for tumorsphere formation assay in empty-vector (Vector) and YTHDC2-overexpressing groups (YTHDC2) of T24. The volume of spheroid in each group was measured with Image J. Quantitative results were presented on the right. Scale bar: 50 µm. **C** Extreme limiting dilution assay in Cas9-NC and YTHDC2 knockout 5637 cells. Fitting curve and statistical significance were both generated from ELDA software. **D** Representative images of the KRT14 immunofluorescence staining results of Cas9-NC and YTHDC2 knockout groups and fluorescence intensity quantification. Scale bar: 20 µm. **E**–**H** Immunofluorescence staining of ALDH1 and CD133 followed by flowcytometry analysis was conducted to identify CD133^+^/ALDH1^+^ stem cell subpopulation derived from cultures of Cas9-NC and YTHDC2 knockout groups of 5637 cells. **I**, **J** Flow cytometric analysis of CD133⁺ cells in T24 bladder cancer cells infected with control (Vector) or YTHDC2-overexpressing lentivirus. **I** Representative flow plots showing the proportion of CD133⁺ cells. **J** Quantification of CD133⁺ cell percentages, showing a significant reduction in the YTHDC2-overexpression group. **K** Representative immunohistochemical staining of CD133 in xenograft tumor sections derived from T24 cells infected with control or YTHDC2-overexpressing lentivirus. A marked reduction in CD133-positive cells was observed upon YTHDC2 overexpression. Scale bar: 20 μm. *P < 0.05, **P < 0.01, ***P < 0.001, ****P < 0.0001.

Bladder cancer recurrence and metastases after treatment have been closely linked to cancer stemness [18, 43]. The association of YTHDC2 with stemness-related genes and WNT signaling suggested its role in regulating cancer stemness. Indeed, tumorsphere formation assays showed that YTHDC2 knockout increased the number and volume of spheroids formed from 5637 single cells (Fig. 3A). Conversely, overexpression of YTHDC2 in T24 cells reduced the volume of spheroids (Fig. 3B). Extreme limiting dilution assays revealed that YTHDC2-KO1 and YTHDC2-KO3 polyclonal cells (with stronger knockout efficiency than YTHDC2-KO2; Fig. 2A) exhibited significantly higher tumor-initiating ability in 5637 cells under low-density conditions (Fig. 3C).

Keratin 14 (KRT14) was identified as a marker of tumor initiating cell subpopulation with stem-like properties in bladder tumorigenesis [44]. Immunofluorescence (IF) analysis revealed significantly elevated KRT14 levels in YTHDC2-knockout groups compared to controls (Fig. 3D). Furthermore, results of flow cytometry demonstrated that YTHDC2 knockout increased the ALDH1^+^ cells and CD133^+^ populations (markers for bladder cancer stem cells) in 5637-derived tumorspheres (Fig. 3E–H), while in T24, overexpression of YTHDC2 decreased the ratio of CD133^+^ subpopulation (Fig. 3I, J). Furthermore, we detected the cancer stemness biomarkers in T24 xenograft tumors using IHC, which showed reduced CD133 levels in YTHDC2-overexpressing tumor tissues compared to controls (Fig. 3K). Taken together, these results highlight YTHDC2’s role as a tumor suppressor by restraining cell stemness and the self-renewal ability.

### SOX2 is a key target gene of YTHDC2

**Fig. 4:**
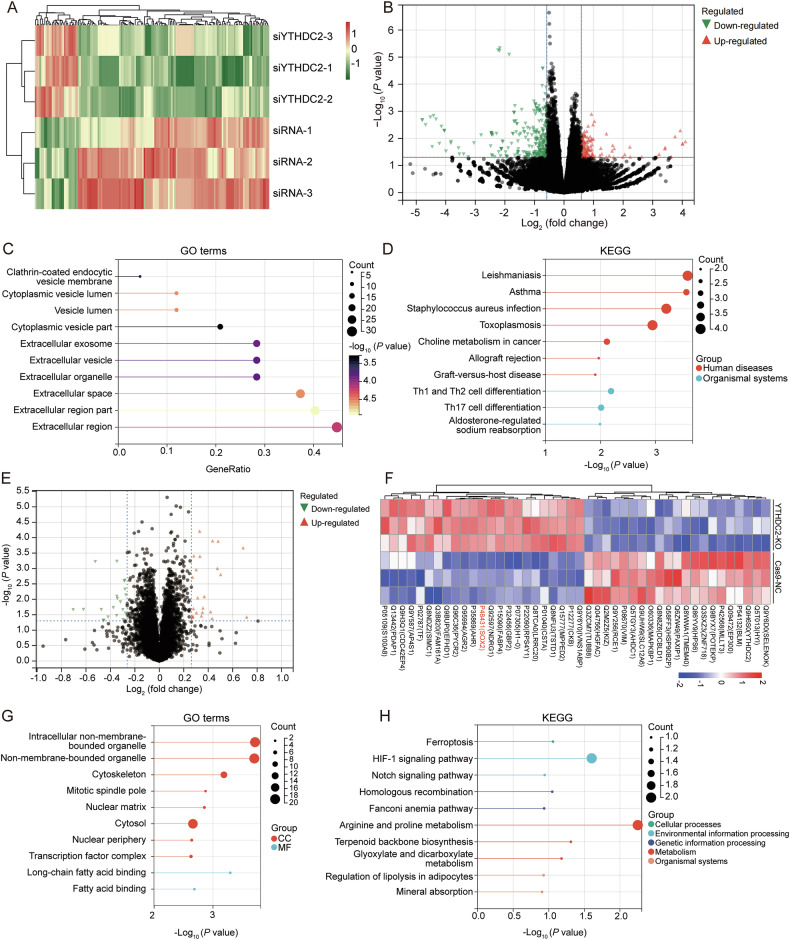
The transcriptomics and proteomics profiles of 5637 cells. **A** The heatmap presented differentially expressed transcripts identified by RNA-seq between siRNA group and si-YTHDC2 group. **B** The volcano plot showed all expression changes of transcripts in the RNA-seq dataset between group of scrambled siRNA and groups of siRNAs targeting *YTHDC2*. |log_2_FC| > 0.585, *P* < 0.05. **C** The lollipop chart showed the GO enrichment analysis of DEGs identified by RNA-seq. **D** The lollipop chart showed the KEGG enrichment analysis of DEGs identified by RNA-seq. **E** The volcano plot showed numbers of up-regulated and down-regulated proteins in the proteomics dataset between groups of Cas9-NC and YTHDC2 knockout 5637 cells. |log2FC| > 0.263, *P* < 0.05. **F** The heatmap presented differentially expressed proteins identified by proteomics between Cas9-NC and YTHDC2 knockout groups. **G** The lollipop chart showed the GO enrichment analysis of differentially expressed proteins identified by proteomics analysis. **H** The lollipop chart showed the KEGG enrichment analysis of differentially expressed proteins identified by proteomics analysis.

As an m^6^A reader, YTHDC2 can regulate both the RNA degradation and protein translation process of target genes [45]. To uncover how YTHDC2 suppresses bladder cancer, we conducted RNA sequencing, proteomics, and m^6^A methylated RNA immunoprecipitation (MeRIP) sequencing to identify the potential downstream effectors. RNA sequencing identified 128 significantly upregulated (log_2_FC > 0.585, *P* < 0.05) and 285 downregulated (log_2_FC < −0.585, *P* < 0.05) transcripts upon YTHDC2 silencing (Fig. 4A, B). The expression patterns, prognostic significance, and pathway enrichment of corresponding differentially expressed genes (DEGs) in TCGA-BLCA tissues are presented in Supplementary Figs. S5–S7. Moreover, GO and KEGG analysis revealed that the differentially expressed mRNAs were significantly enriched in regions such as the extracellular region and extracellular organelle, as well as in pathways related to leishmaniasis and asthma (Fig. 4C, D). These differentially expressed mRNAs and the enriched pathways seem to have little relevance to the YTHDC2-mediated alterations in tumor cell stemness, indicating YTHDC2 may exert its function by regulating protein levels. The proteomics identified 23 up-regulated (log_2_FC > 0.263, *P* < 0.05) and 21 down-regulated (log_2_FC < −0.263, *P* < 0.05) proteins after YTHDC2 knockout (Fig. 4E). Supplementary Fig. S8 shows the prognostic associations and pathway enrichment of these differentially expressed proteins (DEPs) in TCGA-BLCA. SOX2 was one of the up-regulated proteins in YTHDC2 knockout cells (Fig. 4F). GO and KEGG analysis revealed that the differentially expressed proteins were significantly enriched in process like ferroptosis, and molecular functions like transcription factor complex (Fig. 4G, H).

**Fig. 5:**
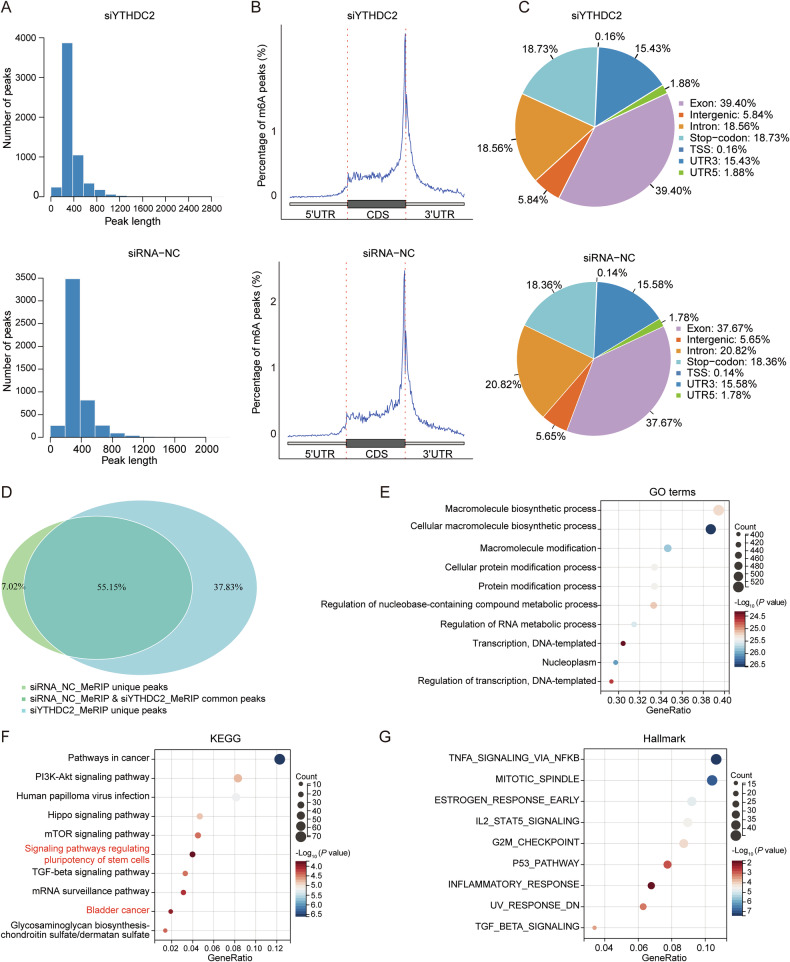
The m^6^A profiles in 5637 cells. **A** Peak length in MeRIP-seq between siYTHDC2 group (top) and siRNA-NC group (bottom). **B** The m^6^A peak distribution on structures of mRNA in MeRIP-seq between siYTHDC2 group (top) and siRNA-NC group (bottom). **C** Pie diagram of m^6^A peak distribution on RNA structure in MeRIP-seq between siYTHDC2 group (top) and siRNA-NC group (bottom). **D** Venn diagram showed the common peaks in MeRIP-seq between siYTHDC2 group and siRNA-NC group. **E** GO enrichment analysis of common peak genes identified by MeRIP-seq between siYTHDC2 group and siRNA-NC group. **F** KEGG enrichment analysis of common peak genes identified by MeRIP-seq between siYTHDC2 group and siRNA-NC group. **G** Hallmark enrichment analysis of common peak genes identified by MeRIP-seq between siYTHDC2 group and siRNA-NC group.

MeRIP-seq profiling of m^6^A modification in 5637 cells with/without YTHDC2 knockdown showed conserved methylation patterns, with no significant differences in peak number or distribution (Fig. 5A–C). The majority of peaks in both groups were 200–400 bps (Fig. 5A), and were located predominantly at the exon of CDS-3’UTR junction (Fig. 5B, C), indicating a post-transcriptional regulation [46]. Notably, 55.15% of m^6^A modified sites were identified as common peaks in control and si*YTHDC2* (Fig. 5D). Interestingly, the common peaks-localized genes were enriched in pathways regulating pluripotency of stem cells and related to bladder cancer (Fig. 5E–G).

**Fig. 6:**
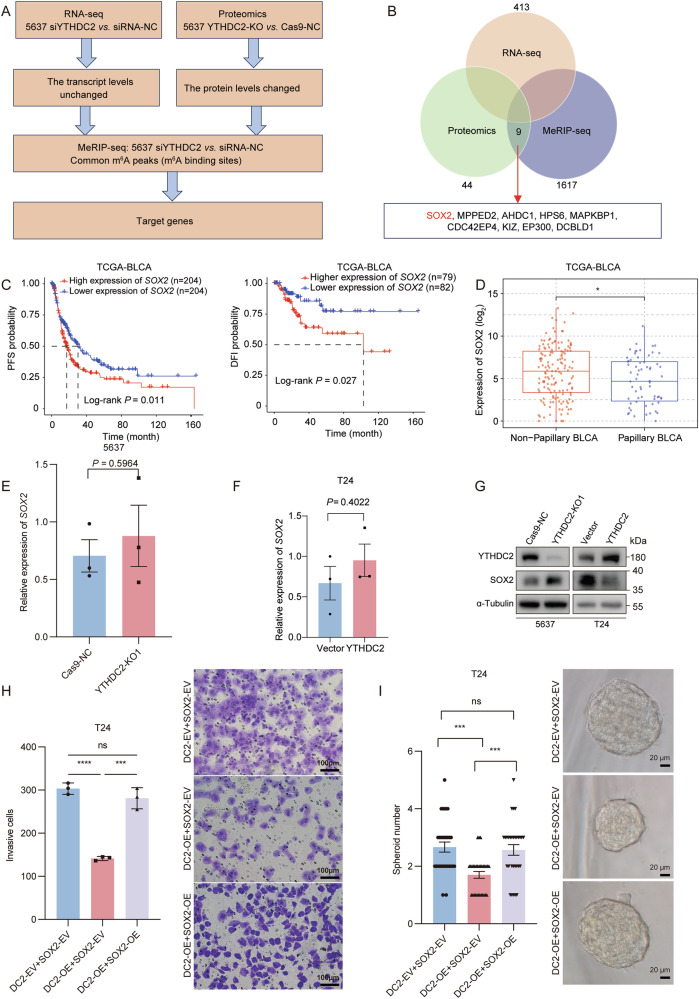
YTHDC2 attenuated SOX2’s expression at protein level but not mRNA level. **A**, **B** Overlap of datasets of common m^6^A peaks from MeRIP-seq, DEPs from proteomics and transcripts with no significant differential expression from RNA-seq. **C** The progression-free survival (left) and disease-free interval (right) of *SOX2* in BLCA patients from TCGA-BLCA dataset. The *P* values for survival analyses were determined using the log-rank test. **D** The mRNA expression of *SOX2* in subtypes of BLCA from TCGA-BLCA dataset. **E** The comparison of mRNA expression of *SOX2* between Cas9-NC and YTHDC2-KO1 groups of 5637 cells by RT-qPCR. Student’s *t* test, *P* = 0.5964. **F** The relative mRNA expression of *SOX2* in T24 cells control group and YTHDC2 overexpression group. Student’s *t* test, *P* = 0.4022. **G** SOX2 protein level changes after YTHDC2 knockout and overexpression respectively in 5637 and T24, visualized by Western blotting. Protein levels were normalized to α-Tubulin levels. **H** SOX2 overexpression rescued the impaired cell invasion ability of T24 by YTHDC2 overexpression. Representative images and quantification of invasive cells in DC2-EV+SOX2-EV, DC2-OE+SOX2-EV, DC2-OE+SOX2-OE groups using transwell invasion assay. Cells that passed through the Matrigel-coated membrane were stained with crystal violet. Data are shown as mean ± SD from three independent experiments, ns not significant. Scale bar, 100 μm. **I** The rescue effect of SOX2 overexpression to the impaired self-renewal ability (cancer stemness) was assessed by tumorsphere formation assay. Representative images and quantification of tumorspheres were obtained and counted under 40× phase contrast microscope. Sphere numbers were counted after 14 days of culture in FBS-free DMEM-F12 medium. Data represent mean ± SD from more than three independent experiments, ns not significant. Scale bar, 20 μm. *P < 0.05, **P < 0.01, ***P < 0.001, ****P < 0.0001.

The above sequencing results revealed that transcriptome changes exhibited limited association with cancer phenotypes, whereas proteomic alterations were more consistent with functional outcomes. Notably, m^6^A peaks were enriched near CDS-3’UTR junctions, suggesting translational regulation. Based on this, we focused on genes with stable mRNA levels but altered protein levels. By integrating transcriptomics, proteomics, and MeRIP-seq data, we identified nine YTHDC2 potential targets (SOX2, MPPED2, AHDC1, HPS6, MAPKBP1, CDC42EP4, KIZ, EP300, DCBLD1) showing protein-level changes and conserved m^6^A modification without RNA level alteration (Fig. 6A, B). Among these 9 potential targets, SOX2-clinically correlated with poor prognosis and aggressive BCa subtypes (Fig. 6C, D)-emerged as a key potential mediator of YTHDC2’s function in bladder cancer. Aligning with RNA sequencing and proteomics results, neither YTHDC2 knockout nor overexpression altered the expression of SOX2 mRNA (Fig. 6E, F). However, YTHDC2 knockout increased SOX2 protein level, whereas overexpression of YTHDC2 decreased the SOX2 protein abundance (Fig. 6G). Together, these results demonstrate YTHDC2 as a post-transcriptional regulator of SOX2.

To functionally validate SOX2 as a downstream target of YTHDC2, rescue experiments were performed by overexpressing SOX2 in YTHDC2-overexpressed T24 cells. Transwell invasion assays showed that SOX2 overexpression restored the invasion capacity of YTHDC2-overexpressed T24 cells (Fig. 6H). In addition, tumorsphere formation assays indicated a significant recovery of self-renewal ability upon SOX2 overexpression (Fig. 6I). These findings validated SOX2 as a key downstream effector mediating YTHDC2’s suppression of cancer stemness and phenotypic plasticity in bladder cancer.

### YTHDC2 binds to SOX2 mRNA and inhibits its translation into SOX2 protein

**Fig. 7:**
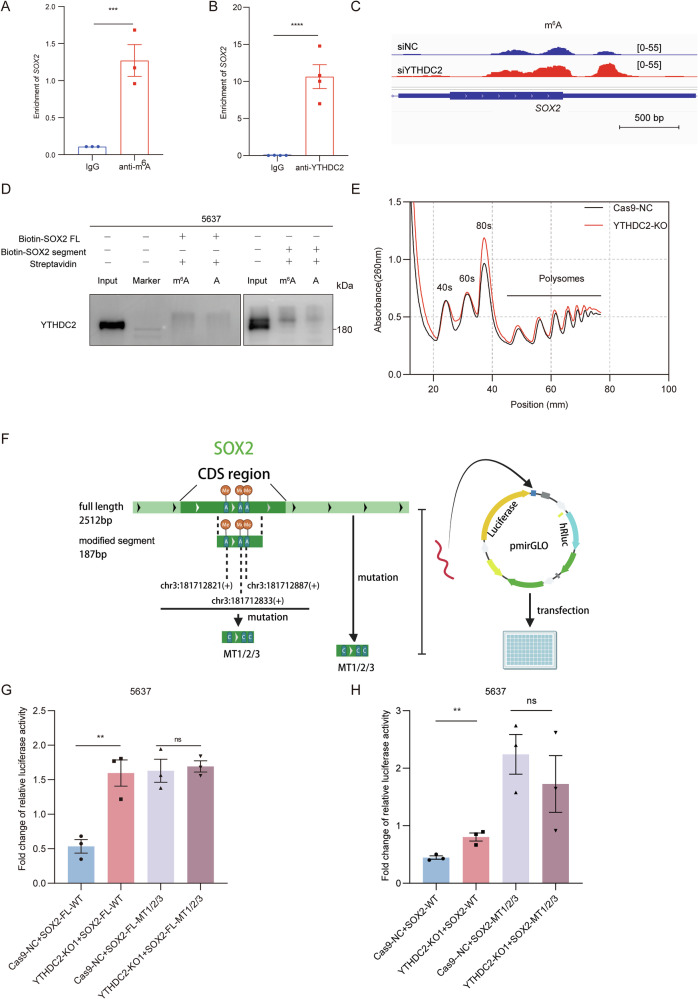
YTHDC2 attenuates SOX2’s gene expression by m^6^A-dependent modulation in translational process. **A** m^6^A modified segments on SOX2 mRNA were enriched by m^6^A antibody and amplified by primers specially designed for predicted modified region on SOX2 mRNA in MeRIP-qPCR assays. **B** RIP-qPCR assays were conducted to demonstrate YTHDC2’s binding to *SOX2* mRNA and amplify the protein bound mRNA. **C** The m^6^A-modified regions on *SOX2* mRNA before and after the silencing of *YTHDC2* by data from MeRIP-sequencing, visualized by IGV_2.18.4 software. **D** RNA pull-down assays and western blot were used to illustrate YTHDC2’s binding preference to m^6^A modified *SOX2* mRNA rather than non-modified one. **E** Polysome profiles of 5637 cells resolved by sucrose gradient centrifugation. Cytoplasmic extracts were subjected to ultracentrifugation through a 10–50% (w/v) sucrose gradient to separate ribosomal subunits, monosomes, and polysomes. Absorbance at 260 nm (A260) was continuously monitored across the gradient. Smoothed curves represent the distribution of ribosomal species, including 40S, 60S, 80S(monosomes), and polysomes. **F** Illustration for point mutations of *SOX2* sequences and plasmid design for DLRAs to unveil mode of YTHDC2’s modulation on SOX2. **G** Luciferase assays were performed by transfecting 5637 Cas9-NC or YTHDC2-KO cells with reporter plasmids containing SOX2 full length sequence, with or without multipoint mutations of three highly potential m^6^A sites, followed by luciferase coding sequences, or an empty vector (EV) containing luciferase only. MT1/2/3 referred to simultaneous multipoint mutation from adenosine(A) to cytosine(C) at site chr3:181712821(+), chr3:181712833(+), and chr3:181712887(+). Luciferase activity was detected after 72 h regular culture of transfected cells. **H** Luciferase assays were performed by transfecting 5637 Cas9-NC or YTHDC2-KO1 stable cell line with pmir-GLO plasmids containing 187 bp potential modified segment of *SOX2* sequence clones with or without multi-point mutations of m^6^A sites followed by luciferase coding sequences or an empty vector (EV) containing luciferase only. Mutation sites are depicted as above. *P  < 0.05, **P < 0.01, ***P < 0.001, ****P  < 0.0001.

We further investigated how YTHDC2 regulated SOX2. Gene Set Enrichment Analysis (GSEA) was performed based on YTHDC2 expression in the TCGA-BLCA cohort. Samples with high YTHDC2 expression showed significant enrichment of gene sets associated with translation repressor activity mRNA regulatory element binding (NES = 1.7463, *P* = 0.004) and *N*^*6*^-methyladenosine (m^6^A)-containing RNA binding (NES = 1.8121, *P* < 0.0001), suggesting a potential role of YTHDC2 in translational regulation and m^6^A-related RNA metabolism in bladder cancer (Supplementary Fig. S9A), suggesting its translational regulation potential. MeRIP-qPCR and agarose gel electrophoresis (AGE) analyses detected m^6^A modification of SOX2 mRNA in 5637 cells, with significant enrichment in m^6^A immunoprecipitates compared to IgG controls (Fig. 7A and Supplementary Fig. S9B, C). RNA immunoprecipitation (RIP) -qPCR further demonstrated YTHDC2’s interaction with SOX2 mRNA (Fig. 7B and Supplementary Fig. S9D), collectively implicating m^6^A-dependent recognition of SOX2 by YTHDC2.

To further confirm that YTHDC2 can directly bind to m^6^A-modified SOX2 mRNA, we performed RNA pull-down assays. Based on our MeRIP-seq data and m^6^A modification site mapping (Fig. 7C), two constructs were cloned into pcDNA3.1(+) vectors: one encoding the full-length SOX2 transcript and the other containing a 187-nt segment enriching m^6^A modifications (Supplementary Fig. S9E). Biotin-labeled RNA probes were transcribed by in vitro transcription using NTPs mixtures with m^6^A-modified adenosine or normal adenosine. Following the RNA pull-down, western blot of the precipitates showed more YTHDC2 enrichment using probes with m^6^A, either in precipitate by full length probe or by 187-nt segment probe (Fig. 7D), suggesting that YTHDC2 has higher affinity to bind to m^6^A-modified *SOX2* mRNA than to unmethylated transcripts.

We further investigated whether YTHDC2 affects the global translation efficiency. Polysome profiling using sucrose gradient fractionation were conducted to assess the translation efficiency difference between 5637 cells with or without YTHDC2 knockout. Polysome profiling directly measure the ribosome-bound mRNA fractions, which is considered as translationally active fractions. Our results revealed that YTHDC2 knockout increased the ribosome loading on 5637 cells’ mRNA (both monosomes and polysomes) (Fig. 7E), indicating enhanced translational efficiency.

To functionally verify YTHDC2’s m^6^A-dependent regulation of SOX2 translation, we performed luciferase reporter assays using both full-length SOX2 sequence (SOX2-FL-WT) and a 187-nt segment containing three predicted m^6^A sites (chr3:181712821/833/887(+)) (Supplementary Table S2), which were identified by integrating our MeRIP-seq data with the RMVar database (Fig. 7F). For the full-length SOX2 reporter (SOX2-FL-WT), YTHDC2 knockout significantly increased luciferase activity, whereas the triple mutant (SOX2-FL-MT1/2/3) remained unaffected (Fig. 7G). Similarly, in the 187-nt m^6^A-enriched fragment, YTHDC2 depletion enhanced wild-type reporter activity, but had no effect on the triple mutant (Fig. 7H). These results demonstrate that YTHDC2’s translational suppression of SOX2 strictly depends on these three m^6^A sites.

## Discussion

**Fig. 8:**
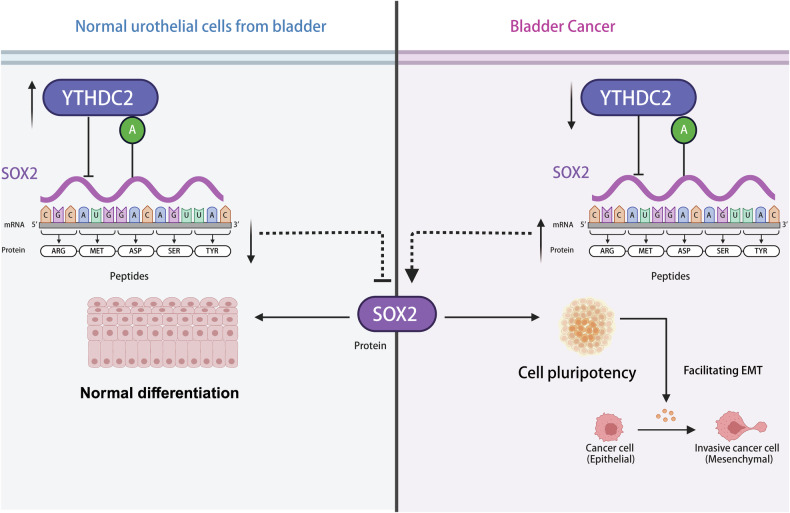
The mechanism of YTHDC2’s function in bladder cancer. A working model to illustrate how YTHDC2 inhibit tumor progression by down-regulating transcription factor SOX2 and restrain the CSC pool and EMT plasticity induced by SOX2. The working model was created in BioRender.com.

In the present study, we identified the m^6^A reader YTHDC2 as a tumor suppressor by inhibiting proliferation, EMT and stemness in bladder cancer. Patients with different subtypes and stages of bladder cancer consistently showed better prognosis when there is higher YTHDC2 expression in tumor. Mechanistically, YTHDC2 inhibits bladder cancer progression by binding to m^6^A-modified *SOX2* mRNA and suppressing SOX2 protein translation (Fig. 8). SOX2 functions in the CSCs’ self-renewal and stemness maintenance in invasive cancer types [47], and its depletion inhibits cancer invasion in cancers including BCa [12, 19, 48]. Our study also confirmed the potential binding site of YTHDC2 on SOX2 mRNA.

The crosstalk between the tumor plasticity induced by CSCs and the microenvironment has been long considered to activate tumor initiation and metastasis [49]. The highly recurrent and invasive features of different bladder cancer subtypes are virtually attributed to various BCSCs transformed by multiple gene alterations [50]. As an m^6^A reader, YTHDC2 was confirmed to regulate both the cell fate of human embryonic stem cells and the cancer development. It can determine hESCs’ neural differentiation by the crosstalk of RNA m^6^A and DNA 5mC [26]. Meanwhile, similar crosstalk of different methylations including YTHDC2-recognized one, has been uncovered in multiple cancers to modulate EMT-related oncogenes and the EMT process [51, 52]. YTHDC2 also influences cell fate determination in BCa. Our results show that YTHDC2 acts as a gatekeeper for CSC induction by restricting SOX2 expression. And our findings combined with others’ have revealed that SOX2 is a key regulator of bladder cancer’s invasion and lineage marker expression[19, 53]. Loss of YTHDC2 removes this restriction, allowing cancer cells to transform into CSCs, as evidenced by increased ALDH1^+^/CD133^+^ CSCs. This expansion of the CSC pool enhances EMT plasticity and invasion, consistent with findings in other cancers.

Cells in EMT process have been proved to be transitional, and there have been researches identifying the existence of subsequent MET after the EMT process to help cancer cell’s adhesion in situ [54]. Of note, it is mainly the population of stem cells in mesenchymal state and prone to metastasis that have been illustrated to possess higher susceptibility to ferroptosis [55]. Based on the two points of view, we assume that BCSCs induced by upregulated SOX2 in our study could have already undergone MET process after the EMT in a certain stage of culture. Yet, due to the limitation of culture time length, we didn’t manage to track the integral process of EMT-MET transition cycle, which in the future may need to be done using primary cells and under the surveillance of single-cell sequencing.

*N*^*6*^-methyladenosine modification of RNA exerts multi-layered regulatory effects throughout gene expression, sometimes collaborating with other methylation modifications. Primarily localized in coding sequences (CDS), m^6^A modulates translation elongation dynamics and cytoplasmic mRNA metabolism [56, 57]. In our study, YTHDC2 modulated SOX2 at translational level in bladder cancer cells, consistent with the established mechanism observed for this m^6^A reader across multiple cancer types. For one thing, YTHDC2 is not located exclusively in nucleus of BCa cell lines but also in cytoplasm with considerable abundance of expression according to our IF staining results (Supplementary Fig. S10A), which allows its diversified regulatory mechanisms and under some circumstances the preference of post-transcriptional regulation. For another, given the rapid phenotypic transitions necessary for stem cells during the EMT-MET cycle [58], regulating effector molecules through translational modulation with fewer steps and less energy consumption, may be more advantageous than relying on complicated crosstalk.

From a translational perspective, our findings raise the possibility of constructing synthetic gene circuits that selectively target cancer cells based on low activity of YTHDC2 expression. Since SOX2 plays a pivotal role in maintaining the self-renewal and differentiation capacities of various stem cell types, direct inhibition of SOX2 carries the risk of disrupting normal stem cell function. Restoring YTHDC2 expression in tumors may provide a safer means of suppressing SOX2-driven stemness and phenotypic plasticity.

In conclusion, our findings suggest that the YTHDC2-SOX2 regulatory axis may contribute to the high rates of recurrence and metastasis by sustaining cancer stem cell properties and enhancing EMT. The frequent downregulation of YTHDC2 in aggressive tumors highlights its potential as a prognostic biomarker. A YTHDC2-based risk stratification model should be prospectively validated against histopathology to optimize clinical decision-making.

## Materials and methods

### Tissue microarray and immunohistochemistry

Tissue microarrays (TMAs) were purchased from Outdo Biotech (Shanghai, China), identified by the array number as HBlaU079Su01. The histopathological diagnoses were independently reviewed by two pathologists and are documented in the patient survival materials, which also include data on the patients’ genders, age, treatment, overall survival, and tissue identification numbers. The TMA comprises a total of 63 BLCA tissue cores and 16 adjacent normal bladder tissue cores. In the analysis of YTHDC2 protein level between tumor and adjacent tissues, three tumor–adjacent pairs were excluded owing to the absence of normal bladder urothelium in the corresponding adjacent samples.

For the immunohistochemistry (IHC) assays, immunostaining aimed at YTHDC2 in the TMA was conducted by incubating tissue cores with an anti-YTHDC2 antibody from Abcam, followed by staining with a secondary antibody. The staining results were evaluated using two criteria: staining intensity, scored from 0 to 3, and the positive rate of staining, scored from 0 to 100%. The IHC score was then calculated by multiplying these two scores to quantify the expression of YTHDC2 in both BLCA and adjacent normal tissues.

### Cell culture

Cell lines, including bladder cancer cell lines such as 5637, UM-UC-3, T24, and J82, were all purchased from the Cell Bank of the Chinese Academy of Science (Shanghai, China). These cell lines were all authenticated using short tandem repeats (STRs) and tested for mycoplasma contamination. The bladder cancer cell lines 5637, T24, and J82 were cultured in RPMI 1640 medium (Gibco, Life Technology, Carlsbad, CA, USA) supplemented with 10% fetal bovine serum (FBS, Gibco) and 1% Penicillin-Streptomycin-Amphotericin B Solution (Beyotime Biotechnology, Shanghai, China). In contrast, UM-UC-3 was maintained in Dulbecco’s modified Eagle’s medium (DMEM, Gibco) with the same supplements. Additionally, tumorspheres derived from these cell lines were cultivated in the DMEM/Nutrient Mixture F-12 (DMEM/F-12) containing 0.2% B27 (Gibco), 20 ng/ml human Epidermal Growth Factor (hEGF), 10 ng/ml human Fibroblast Growth Factor (hFGF), 5 µg/ml insulin (Beyotime), and 0.4% Bovine Serum Albumin (BSA, Solarbio, Beijing, China). All cells were maintained in an incubator at 37 °C with 5% CO_2_.

### Lentivirus, plasmid construction and cell transfection

For YTHDC2 knockout, 5637 cell lines were transduced with CRISPR/Cas9 lentivirus containing sgRNA targeting the YTHDC2 gene, along with a corresponding control lentivirus (Genechem, Shanghai, China). For overexpression, lentiviral vectors harboring flag-tagged YTHDC2 cDNA (Genechem) were introduced into the T24 cell line. All stable cell lines were selected using 1 μg/ml puromycin (Biosharp, Anhui, China).

For dual luciferase reporter assays, pmirGLO vectors containing the SOX2 cDNA clone were synthesized by Sangon Biotech (Shanghai, China). For the RNA pull-down and rescue experiments, the SOX2 overexpression plasmid was constructed by inserting the full-length SOX2 sequence or segment of modified region into the pcDNA3.1(+) vector as showed in the illustration. All plasmids were transfected into cells using Lipofectamine 2000 (Invitrogen, Thermo Fisher Scientific).

### Western blot and antibodies

Cells were lysed using RIPA lysis buffer supplemented with protease and phosphatase inhibitors (Beyotime) to extract total protein. The clarified lysates were then separated by SDS-PAGE and transferred onto a 0.2 μm pore size PVDF membrane (Millipore). Membranes were blocked using 5% skim milk dissolved in TBST. Overnight incubation at 4 °C with the following primary antibodies were performed: anti-α-Tubulin (ab176560, Abcam), anti-YTHDC2 (ab220160, Abcam), anti-SOX2 (ab92494, Abcam), anti-E-cadherin (A3044, Abclonal), and anti-N-cadherin (A19083, Abclonal). HRP-labeled goat anti Rabbit IgG (A0208, Beyotime) were used as secondary antibody for membrane incubation. The specific bands were visualized using the Essential V6 imaging platform from UVITEC (Cambridge, UK).

### RNA extraction and RT-qPCR analysis

Total RNA was extracted by using Eastep Super Total RNA Extraction Kit (Promega, Madison, Wisconsin, USA) and the concentration of total RNA was quantified using Nanodrop spectrophotometers (Thermo Fisher Scientific). The reverse transcription reaction was performed using PrimeScript RT reagent Kit with gDNA Eraser (Takara, Japan). RT-qPCR analysis was performed by utilizing Genious 2X SYBR Green Fast qPCR Mix (Abclonal) and CFX Connect Real-Time PCR Detection System (Bio-Rad). All the primers utilized in RT-qPCR were synthesized by Sangon Biotech (Shanghai, China) and sequences were listed in the Supplementary Table S1.

### Cell counting kit-8 (CCK-8) assay

CCK-8 assays were employed to measure cell viability. Cells were plated in 96-well plates at a density of 1.5 × 10^3^ cells per well and incubated at 37 °C. Cell viability was assessed by the optical density (OD) measured in each well after adding CCK-8 solution (Dojindo Laboratories, Kumamoto, Japan) and incubating for 3 h.

### Colony formation assay

Cells ranging from 1 × 10^3^ to 1.5 × 10^3^, depending on the optimal seeding density for different cell lines, were plated in six-well plates (Corning, New York, USA) and incubated at 37 °C with 5% CO_2_. The medium, containing 10% FBS, was refreshed every three days. Two weeks after inoculation, cells were washed with PBS (Biosharp) at room temperature. Cells were then fixed for 30 min using 4% paraformaldehyde solution (Biosharp) and stained for 15 min with crystal violet staining solution (Beyotime). All experiments were performed in triplicate.

### Transwell-migration and invasion assay

Cells were trypsinized and resuspended in FBS-free medium. The resuspended cells were added with a density of 4 × 10^4^–8 × 10^4^ cells/well to transwell inserts of 8 μm (Corning) settled in 24-well plates where each well was added for 500 μl medium containing 10% FBS. After 24 h of culture in the incubator, cells migrating from the apical chamber to basolateral chamber were fixed by 4% paraformaldehyde and stained by crystal violet. Cells were quantified using Image J software.

### Tumorsphere formation assay

Dissociated cells were resuspended in D-PBS (Biosharp) as single cell suspension and inoculated to ultra-low attachment 96-well plates (200 cells/well) or 6-well plates (1 × 10^5^/well) (Corning) for sphere formation assay and flow cytometry. Sphere deriving cells were incubated in humidified atmosphere at 37 °C with 5% CO_2_ for 10–14 days. Formed spheres in optimal morphology were observed and quantified under inverted phase contrast microscope from Olympus.

### Extreme limiting dilution assay

For in vitro limiting dilution assays, PBS-resuspended single cells were seeded to an ultra-low attachment 96-well plates (Corning) and cultured in the sphere medium. After 14 days of incubation, cell line derived spheres were counted under inverted microscope and the non-responding rates were calculated by online bioinformatic tool *ELDA* from https://bioinf.wehi.edu.au/software/elda/index.html to perform the statistical analysis and generate the linear graph.

### Subcutaneous xenografts in nude mice

Four-week-old male Balb/c nude mice purchased through BIONT Biological Technology (Hubei, China) were randomly divided into two groups. Cells (5 × 10^6^) resuspended in the solution containing Matrigel and PBS in a ratio of 1:1 were injected subcutaneously into nude mice with the volume of 200 μl. The tumor volume and mouse weight were measured and were estimated by using the formula: V = 0.5 × D × d^2^ (V, volume; D, longitudinal diameter; d, latitudinal diameter). Mice were euthanized before the tumor size exceeded the requirements of animal ethics.

All animal experiments were ethically performed and authorized by the Experimental Animal Welfare Ethics Committee of Zhongnan Hospital of Wuhan University (ZN2021113). Group sizes were determined based on previous studies using similar animal models and are consistent with commonly accepted standards in the field. All eligible animals meeting quality control thresholds were included. No animals were excluded from the analysis.

### RNA sequencing

RNA sequencing was performed by RiboBio (Guangzhou, China). The standard total RNA samples for sequencing were extracted from about 1 × 10^6^ cells with RNeasy mini kit (QIAGEN, Düsseldorf, German) and were maintained at −80 °C before sequencing. The transcriptome sequencing was executed with high-throughput DNBSEQ sequencing technology platforms, followed by high-resolution digital imaging. The data analysis was performed through standard and customized bioinformatics pipelines. Raw data files were set as format of fastq.

### Proteomics analysis

TMT quantitative proteomics were delegated to Novogene (Beijing, China) for execution. Pellets containing 3 × 10^6^ cells for each sample were maintained in dry ice during transportation. Peptides from at least 110 μg total protein per sample were labeled with TMT and the mass spectrometry analysis was performed using the Q Exactive HF-X Hybrid Quadrupole-Orbitrap MS System from Thermo Fisher Scientific. The raw datasets from the MS analysis were matched with Uniprot database and the quantification data were used for the differential expression analysis as well as further function annotations and enrichment analysis.

### MeRIP sequencing and MeRIP-qPCR

The m^6^A modified RNA was enriched using m^6^A methylated RNA immunoprecipitation assays. For m^6^A profiling, MeRIP-seq was delegated to RiboBio (Guangzhou, China). RNA samples from 5637 cells were fragmented into size of ~200 bp and prepared for m^6^A methylated RNA immunoprecipitation assays by riboMeRIP m^6^A Transcriptome Profiling Kit (C11051-1, RiboBio) followed by library construction and high-throughput NGS sequencing.

For indicated m^6^A peaks validation, the fragmentation and immunoprecipitation were realized by using Magna MeRIP m^6^A Kit (Millipore). 300 μg total RNA per sample was extracted from 5637 cell pellets with RNeasy mini kit and were fragmented into size of 200 ~ 300 bp at 94 °C for 30 s. 300 μg fragmented RNA per sample was precipitated by 10 μg Anti-m^6^A (Part# MABE1006, Millipore) conjugated with ChIP Protein A/G Magnetic Beads (Part# CS203152, Millipore). After elution and purification, the precipitated m^6^A modified RNAs were analyzed by quantitative RT-PCR. The primers involved were designed for sequencing-identified m^6^A motif on mRNA of SOX2 and the PCR products were visualized by Agarose gel electrophoresis (AGE).

### RNA immunoprecipitation (RIP) assays

RIP assays were performed according to the Users Guides of Magna RIP^TM^ RNA-Binding Protein Immunoprecipitation Kit (Millipore). PBS-washed cell pellets were lysed with RIP lysis buffer followed by centrifugation. The supernatant from the lysate was incubated with beads-antibody complex to initiate the reaction. Subsequently, the immunoprecipitated RNA went through purification of which the products were eventually used for RT-qPCR to amplify the cDNA of YTHDC2-bound transcripts like *SOX2* with specific primers, followed by visualization with AGE.

### In vitro transcription and RNA pull-down assay

For synthesis of biotin-labeled RNA probes, in vitro transcription (IVT) was performed with the manufacturer’s instruction of Biotin RNA Labeling Mix (Roche, Basel, Switzerland). Briefly, 1 μg linearized plasmids with clones of *SOX2* or its modified segment per reaction were utilized as templates to synthesize biotin-labeled RNA probes with NTP labeling mixture under the catalyzation of T7 polymerase (Roche). By controlling the supply of *N*^*6*^-methyladenosine to the transcription system, each probe had been transcribed into two versions, respectively m^6^A modified and non-modified.

For in vitro RNA pull-down assays, supernatant from 5637 cell lysates was separated after centrifugation for the following pull-down procedure. Biotinylated RNA probes were incubated with dynabeads streptavidin (Invitrogen, Thermo Fisher Scientific) to promote the biotin-streptavidin combination. The probe-beads mixture was then added to the supernatant so as to activate the RNA-protein interaction. Proteins pulled down by RNA probes were eluted and identified by western blot.

### Dual luciferase reporter assay

Dual luciferase reporter assays (DLRAs) were used to assess the regulation of YTHDC2 to the expression of SOX2. Firstly, the wild type or mutant types sequences of respectively SOX2 and modified section of SOX2 were inserted into the pmirGLO Vector to build the transient expression constructs in KO and control groups of 5637 cells. After incubation in BeyoGold™ 96-Well White Opaque Plates (FCP968) for 72 h, the firefly and renilla luciferase activities in cells were detected with Dual-Lumi™ II Luciferase Reporter Gene Assay Kit (RG089S) and the luminescence was measured by GloMax® 20/20 Luminometer (E5311, Promega).

### Polysome profiling Assay

Polysome extraction buffer (PEB) was prepared with 20 mM Tris-HCl (pH 7.5), 50 mM NaCl, 10 mM MgCl₂, 1 mM DTT, 100 μg/ml cycloheximide (CHX), and 200 μg/ml heparin. Sucrose density gradients (10–50%) were manually prepared in PEB without Triton X-100. Gradients were ultracentrifuged using a Beckman Optima L-70 ultracentrifuge (Beckman Coulter, 344059) with an SW41 Ti rotor, and fractionation was performed using a BR-188 Density Gradient Fractionation System (BioComp Instruments), according to the manufacturer’s instructions.

Approximately 5 × 10⁷ 5637 cells were cultured in complete RPMI-1640 medium. Prior to harvest, cells were treated with 100 μg/ml CHX for 10 min at 37 °C to stabilize ribosome–mRNA complexes. Cell pellets were lysed in PEB supplemented with 1% Triton X-100, vortexed for 15 s, and incubated on ice for 30 min. Lysates were then centrifuged at 14,000 rpm for 30 min at 4 °C, and the supernatants were collected. One milliliter of clarified lysate was loaded onto the prepared sucrose gradients. Tubes were sealed with parafilm and centrifuged at 38,000 rpm for 2 h at 4 °C in an SW41 Ti rotor. Following centrifugation, gradients were fractionated using the BR-188 system and absorbance at 260 nm was recorded to generate polysome profiles.

### Stem cell immunofluorescence labeling and flow cytometry

Hallmarks CD133 and ALDH1 were selected for bladder cancer stem cell labeling in population of 5637 and T24 cell lines [59]. For ALDH1 labeling, cells were stained by using ALDEFLUOR Kit (#01700, STEMCELL Technologies, Vancouver, Canada) according to the manufacturer’s instructions. For CD133 labeling, cells going through ALDH assays were incubated with anti-CD133 (566596, BD Biosciences, New Jersey, USA).

Labeled single cell suspension were analyzed through a NovoCyte Flow Cytometer Systems from Agilent (Santa Clara, CA, USA) and the data were processed by software *CytoExpert*.

### Immunofluorescence staining

Immunofluorescence (IF) assay was performed to assess protein levels and protein subcellular localization. Briefly, samples were fixed, permeabilized, and blocked prior to immunostaining. The antibodies and reagents used for staining were respectively anti-YTHDC2 (Sigma), anti-Cytokeratin 14 (Proteintech), Antifade Mounting Medium with DAPI (P0131, Beyotime).

### Bioinformatics and multi-omics analyses

To investigate the clinical relevance, regulatory mechanisms, and biological functions of YTHDC2 in bladder cancer (BLCA) and other cancer types, we conducted a comprehensive bioinformatics analysis by integrating multi-omics data from publicly available resources. Raw and processed data were retrieved from The Cancer Genome Atlas (TCGA), Genotype-Tissue Expression (GTEx), Gene Expression Omnibus (GEO), and TARGET databases.

Expression analysis across normal tissues and cancers: Expression levels of YTHDC2 in normal and tumor tissues were analyzed using RNA-seq data from TCGA and GTEx (via UCSC Xena and TNMplot platforms [60]). Differential expression between tumor and normal, as well as across tumor grades, stages, recurrence status, and molecular subtypes (e.g., NMIBC vs. MIBC), were visualized with box plots and violin plots. Statistical significance was assessed using Student’s *t* test or Wilcoxon rank-sum test with a significance cutoff of *P* < 0.05.

Survival analysis: To evaluate the prognostic value of YTHDC2, Kaplan–Meier survival analyses were performed in both pan-cancer and BLCA cohorts. Patients were stratified into high and low expression groups using the optimal cutoff point automatically determined by maximally selected rank statistics, to enhance sensitivity in detecting prognostic differences. Overall survival (OS), progression-free survival (PFS), disease-free interval (DFI), and disease-specific survival (DSS) were analyzed using the Kaplan–Meier Plotter [61] (https://kmplot.com). Hazard ratios (HRs) and 95% confidence intervals (CIs) were calculated. Subgroup survival analysis was also performed in patients with high tumor mutation burden and different clinical stages.

Mutation correlation and tumor suppressor association: Expression correlation between *YTHDC2* and key tumor suppressors (e.g., *TP53*, *PTEN*, *RB1*) was analyzed using Spearman’s rank correlation in TCGA-BLCA and GEO (GSE13507) cohorts. Results were visualized with linear regression scatter plots and fitted confidence intervals.

Differential gene expression and protein levels: mRNA-level differentially expressed genes (DEGs) and proteomics-based differentially expressed proteins in BLCA were obtained from LinkedOmics [62]. DEGs were defined using the cutoff |log₂FC| > 1 and adjusted *P*-value < 0.05 (Benjamini–Hochberg FDR). Volcano plots were generated using the EnhancedVolcano R package. Overlapping DEGs and DEPs were used for downstream enrichment and pathway analysis.

Functional enrichment and pathway analysis: GO term, KEGG pathway, and MSigDB hallmark enrichment analyses were performed using the Database for Annotation, Visualization and Integrated Discovery (DAVID) tool [63] (https://david.ncifcrf.gov) and visualized with ggplot2 in R. Enrichment significance was defined at *FDR* < 0.05. Enrichment results of YTHDC2-related DEGs, high-correlation genes, and cancer genes were separately analyzed. For Hallmark gene sets, normalized enrichment scores (NES) and *P*-values were calculated using GSEA (Gene Set Enrichment Analysis).

Gene correlation and volcano-based significance ranking: Pearson correlation coefficients between YTHDC2 and all protein-coding genes in TCGA-BLCA were calculated using R (v4.2.1), and the significance was visualized as volcano plots, where cancer-related genes were highlighted after cross-referencing the UTHealth Catalogue of Cancer Genes (UTHealth) [64]. For each gene, we calculated the Pearson correlation coefficient and the corresponding *P*-value relative to YTHDC2 expression levels across the cohort. Genes with |Pearson coefficient|> 0.3 and adjusted *P*-value < 0.05 were considered significantly correlated. Positively and negatively correlated genes were used for downstream functional enrichment analyses.

Pathway activity inference: Potential regulatory roles of YTHDC2 and candidate genes in cancer-related pathways were inferred using PathwayMapper, CancerSEA, and GSCALite. Predicted activation/inhibition of pathways was based on gene set-level enrichment scores (GSVA) and correlation with key hallmark gene sets. Heatmaps were generated to show the consistency and specificity across genes and pathways.

### Statistical analysis

All the statistical analyses were performed with GraphPad Prism 8.0.2 software (GraphPad Software, San Diego, CA, USA). Statistical tests were selected based on the assumed data distribution and group size. Group differences were assessed using Student’s *t* test, non-parametric tests (Mann–Whitney U/Kruskal–Wallis), repeated measures analysis of variance (ANOVA), or one-way ANOVA, as appropriate. A two-sided *P*-value less than 0.05 was taken as statistically significant. Each experiment was performed in triplicate or more unless otherwise noted.

## Supplementary information

Supplementary figure legends and table footnotes

uncropped original western blots

Supplementary figures

Table. S1. oligonucleotide and sequences inserted in plasmid

Table. S2. m6A motifs prediction

## Data Availability

All data needed to evaluate the conclusions are present in the paper. Full and uncropped western blots are provided in the file “uncropped_original_western_blots.pdf” uploaded as supplementary material.
