# Supplementary material for: YTHDC2 suppresses bladder cancer by inhibiting SOX2-mediated tumor plasticity
Source: Cell Death Dis. 2025 Oct 27;16(1):765. doi: 10.1038/s41419-025-08079-w (PMC12559364; doi:10.1038/s41419-025-08079-w)
Supplement: Supplementary file 1 — Supplementary figure legends and table footnotes [file 41419_2025_8079_MOESM1_ESM.docx]

**Supplementary figures and tables**

**Fig. S1 High expression of *YTHDC2* correlates with better survival in cancer patients. (A -B)** Kaplan–Meier (K-M) survival analysis of bladder cancer patients in TCGA-BLCA cohort stratified by YTHDC2 expression. **A** Stage 2 patients (*n* = 130), showing significantly better overall survival in the high YTHDC2 expression group (HR = 0.50, 95% CI: 0.26–0.97; *P* = 0.038). **B** Patients with high tumor mutation burden (*n* = 204), also showing improved prognosis with higher YTHDC2 expression (HR = 0.59, 95% CI: 0.38–0.93; *P* = 0.021). Statistical analysis was performed using the log-rank test. **C** K-M curves showing the prognostic significance of YTHDC2 expression in multiple cancer types from the TCGA cohort. Patients were stratified into high and low expression groups based on the YTHDC2 expression levels. Higher YTHDC2 expression was significantly associated with improved overall survival in: Head and Neck Squamous Cell Carcinoma (TCGA-HNSC) (HR = 0.65, *P* = 0.0065), Kidney Renal Clear Cell Carcinoma (TCGA-KIRC) (HR = 0.62, P = 0.0027), Lung Adenocarcinoma (TCGA-LUAD) (HR = 0.61, *P* = 0.0093), Rectum Adenocarcinoma (TCGA-READ) (HR = 0.21, *P* = 1.8 × 10⁻⁵), Uterine Corpus Endometrial Carcinoma (TCGA-UCEC) (HR = 0.54, *P* = 0.0043). Log-rank tests were used to determine statistical significance. **D** Representative images of YTHDC2 IHC staining in tissues from different T stage bladder cancer(left), and the quantification of the staining by IHC score. Scale bar: 50 μm. Data were analyzed with Mann Whitney U test, *P* < 0.05. Tis, Tumor in-situ.

**Fig. S2 Correlation analysis between YTHDC2 and canonical tumor suppressor genes (TP53, PTEN, and RB1) in bladder cancer. A** Spearman correlation in the TCGA-BLCA cohort shows that YTHDC2 expression is positively associated with PTEN (r = 0.440), RB1 (r = 0.389), and to a lesser extent TP53 (r = 0.201) (*P* < 0.0001 for all). **B** Independent validation in the GSE13507 dataset confirms similar positive correlations between YTHDC2 and PTEN (r = 0.435), RB1 (r = 0.498), and TP53 (r = 0.167). Linear regression fits are shown with 95% confidence intervals (dotted lines). *P* < 0.05 for all.

**Fig. S3 Gene expressions in TCGA-BLCA associated with YTHDC2 and corresponding functional enrichment analysis.** **A** Volcano plots showed the positively and negatively correlated genes with YTHDC2. Data from TCGA-BLCA dataset were analyzed by using the LinkedOmics online tool (Pearson coefficient > 0.3 and *P* < 0.001 were considered as positive, Pearson coefficient < -0.3 and *P* < 0.001 were considered as negative). **B** Lollipop chart showed the GO functional enrichment analysis results of identified YTHDC2-correlated genes. GO terms were categorized by Biological Process, and Cellular Component. Terms with -log10(p-value) > 1.3 (*P* < 0.05) are considered significantly enriched and are indicated with colored lollipops. **C** Lollipop chart showed the KEGG enrichment analysis results of identified YTHDC2-correlated genes. Terms were categorized as in the chart and the conditions of significance are as stated in (**B**). **D** Bubble chart showed the Hallmark enrichment results of identified YTHDC2-correlated genes. **E** Volcano plots showed the cancer-related genes correlated with YTHDC2 in TCGA-BLCA dataset generated from LinkedOmics (Pearson coefficient > 0.3 and *P* < 0.001 were considered as positive, Pearson coefficient < -0.3 and *P* < 0.001 were considered as negative). **F** to **H** Bubble charts from (**F**) to (**H**) respectively showed the GO, KEGG, Hallmark enrichment analysis results of identified cancer-related genes associated with YTHDC2 in (**E**).

**Fig. S4 Supplementary functional verification of YTHDC2’s suppressor role *in vitro* and *in vivo*. A** The protein expression of YTHDC2 in BLCA cell lines. Protein expression levels were normalized to GAPDH. **B** CCK-8 assay showed that YTHDC2 knockout promoted 5637’s cell proliferation, OD value was detected at 450nm of wave length. Data were analyzed using repeated measures analysis of variance (ANOVA) followed by Tukey’s multiple comparisons test. *P* < 0.0001 for all. **C** Images of xenograft tumors dissected from nude mouse at the 35th day after the subcutaneous cell injection of YTHDC2-overexpressing or control T24 cells. **D** Representative images of xenograft tumor tissue dissected from nude mice, stained by hematoxylin and eosin (H&E). Scale bar: 50 µm. **E** Samples of tumor dissected from footpad of nude mice; upper row displays samples of control group injected with empty-vector transfected T24 cells and lower row display the experimental group injected with YTHDC2-overexpressed T24 cells. Tumor weight was measured and compared by Student’s *t*-test, **P* < 0.05**. F** Volume of tumor in-situ were monitored for 28 days before euthanasia of mice. Statistical significance was assessed using two-way ANOVA with Tukey’s multiple comparisons test. *P* < 0.0001. **G** Representative immunohistochemical staining of Ki-67 in tumor tissues derived from T24 cells infected with control or YTHDC2-overexpressing lentivirus. Scale bar: 20 μm.

**Fig. S5 The** **DEGs identified both by our RNA sequencing and in TCGA-BLCA and their clinical relevance. A** Dot plot showing RNA sequencing DEGs between tumor and normal tissues in TCGA-BLCA and GTEx, ranked by log₂ (fold change). Circle size represents the statistical significance (FDR). Input dataset, DEGs from RNA sequencing of si-NC and si-YTHDC2 5637 cells from our study. Threshold, FDR≤0.05. **B** Differential expression of genes between papillary and non-papillary subtypes of bladder cancer (BLCA). Input dataset, DEGs from RNA sequencing of si-NC and si-YTHDC2 5637 cells from our study. **C** Differential expression of genes among different pathological tumor stages (stage 2, 3, 4). Dot color indicates log₁₀(FDR), and size denotes significance levels (FDR < 0.05, < 0.01, or < 0.001). Input dataset, DEGs from RNA sequencing of si-NC and si-YTHDC2 5637 cells from our study.

**Fig. S6** Results of survival analyses of BLCA patients stratified respectively by indicated gene’s expression, including the difference of DFI, DSS, OS and PFS. Significant, cox *P* value ≤ 0.05. Gene set used, DEGs from RNA sequencing of si-NC and si-YTHDC2 5637 cells from our study. Survival data used, survival data from TCGA-BLCA.

**Fig. S7** **Pathway activity analysis based on expression levels of DEGs and correlation with YTHDC2 in bladder cancer in Fig. S6. A** Predicted regulatory effects of DEGs from Fig. S6 on cancer-related pathways in TCGA-BLCA. Each cell indicates the percentage of samples in which a given gene is predicted to activate (red) or inhibit (blue) a specific pathway. Pathway activity scores were estimated per sample using reverse-phase protein array (RPPA)-based models, and samples were grouped according to gene expression levels to evaluate differences in pathway activation. Only genes with statistically significant associations (FDR ≤ 0.05) with at least one pathway are shown. **B** Pairwise Spearman correlation analysis of activity scores among cancer-related pathways in TCGA-BLCA. Pathway scores were derived using Gene Set Variation Analysis (GSVA). The color scale indicates the correlation coefficient (*r*), with positive correlations in red and negative correlations in blue. Statistically significant associations are indicated as *P* ≤ 0.05 (*), FDR ≤ 0.05 (#).

**Fig. S8 Clinical significance and functional pathway predictions of proteomics-identified DEPs using data from TCGA-BLCA. A** Dot plot showing the association between protein-level DEPs from our proteomics data and survival in TCGA-BLCA, including DFI, DSS, OS, and PFS. Dot size reflects statistical significance (Cox *P* value), and color indicates hazard ratio (HR). Significant, Cox *P* value ≤ 0.05. **B** Predicted regulatory effects of DEPs on cancer-related pathways.

**Fig. S9 Supplementary experimental results for mechanism exploration of YTHDC2’s modulation to SOX2. A** GSEA analyses shows DEG *YTHDC2* was enriched in signaling pathway of translation repressor activity mRNA regulatory element binding and *N^6^*-methyladenosine containing RNA binding in group using TCGA-BLCA data. **B** MeRIP-qPCR were used to verify the m^6^A modification on SOX2 mRNA by MeRIP-seq profiling. Primers were specially design for full length SOX2 mRNA. Enrichment was quantified relative to the Cq value of input. **C** Visualization by AGE (agarose gel electrophoresis) of MeRIP-qPCR enrichments. Replicates = 3. **D** Visualization by AGE of RIP-qPCR products enriched by anti-YTHDC2. Replicates = 4. **E** Illustration to briefly summarize the process of RNA pull-down by specific probes. The illustrations were created in BioRender.com.

**Fig. S10 A** Representative images of the IF staining for localization of YTHDC2 protein expression within cells of 5637 and T24. Cells were simultaneously stained with DAPI to visualize the nuclei. Scale bar: 20 μm.

**Table S1.** Primers and sequences of plasmids used in research.

**Table S2.** Results of m^6^A motifs prediction with data from open-access methylation database RMVar after screening by the modified region mapped by MeRIP-seq.
