# Supplementary material for: YTHDC2 suppresses bladder cancer by inhibiting SOX2-mediated tumor plasticity
Source: Cell Death Dis. 2025 Oct 27;16(1):765. doi: 10.1038/s41419-025-08079-w (PMC12559364; doi:10.1038/s41419-025-08079-w)
Supplement: Supplementary file 2 — uncropped original western blots [file 41419_2025_8079_MOESM2_ESM.pdf]

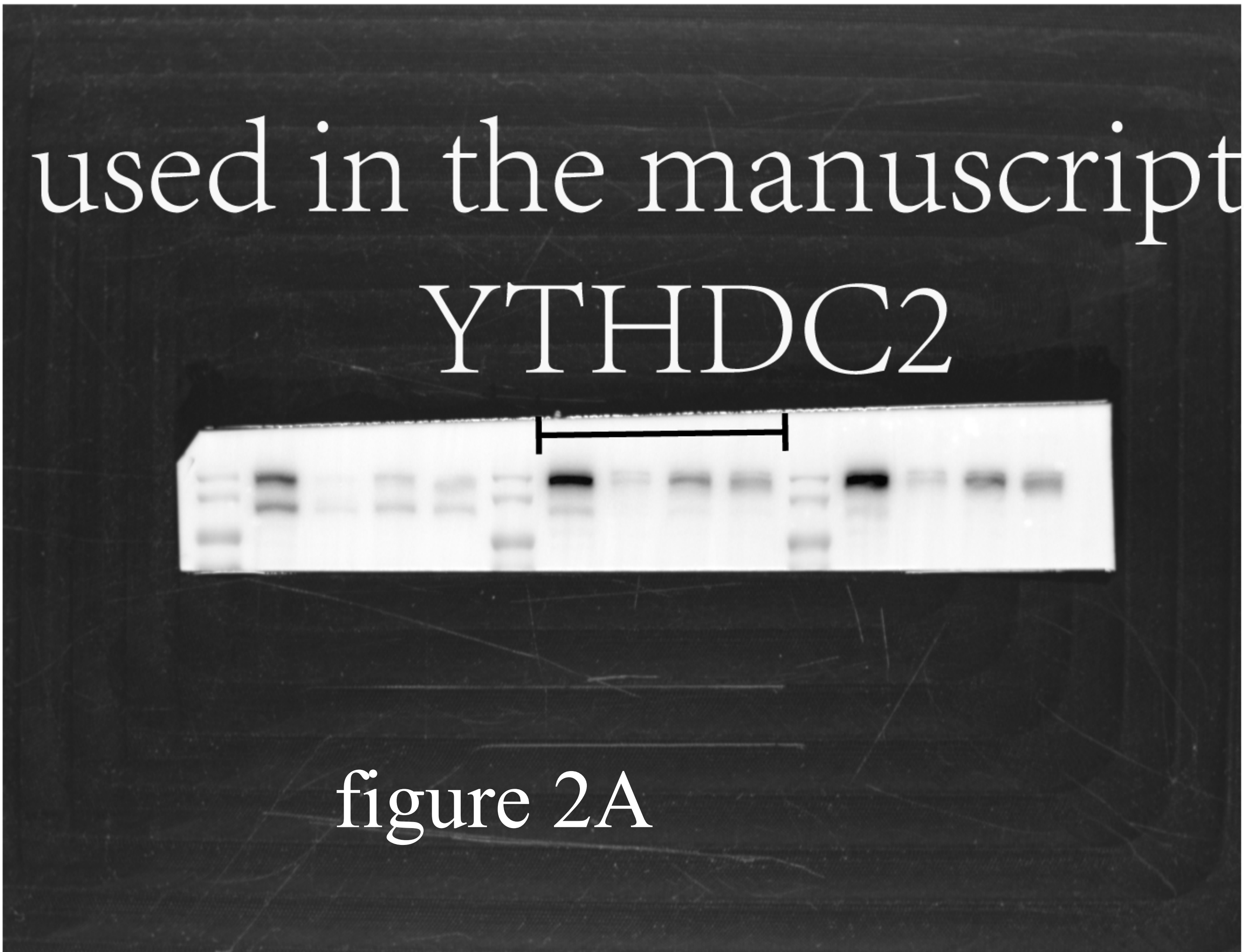
used in the manuscript
YTHDC2
figure 2A

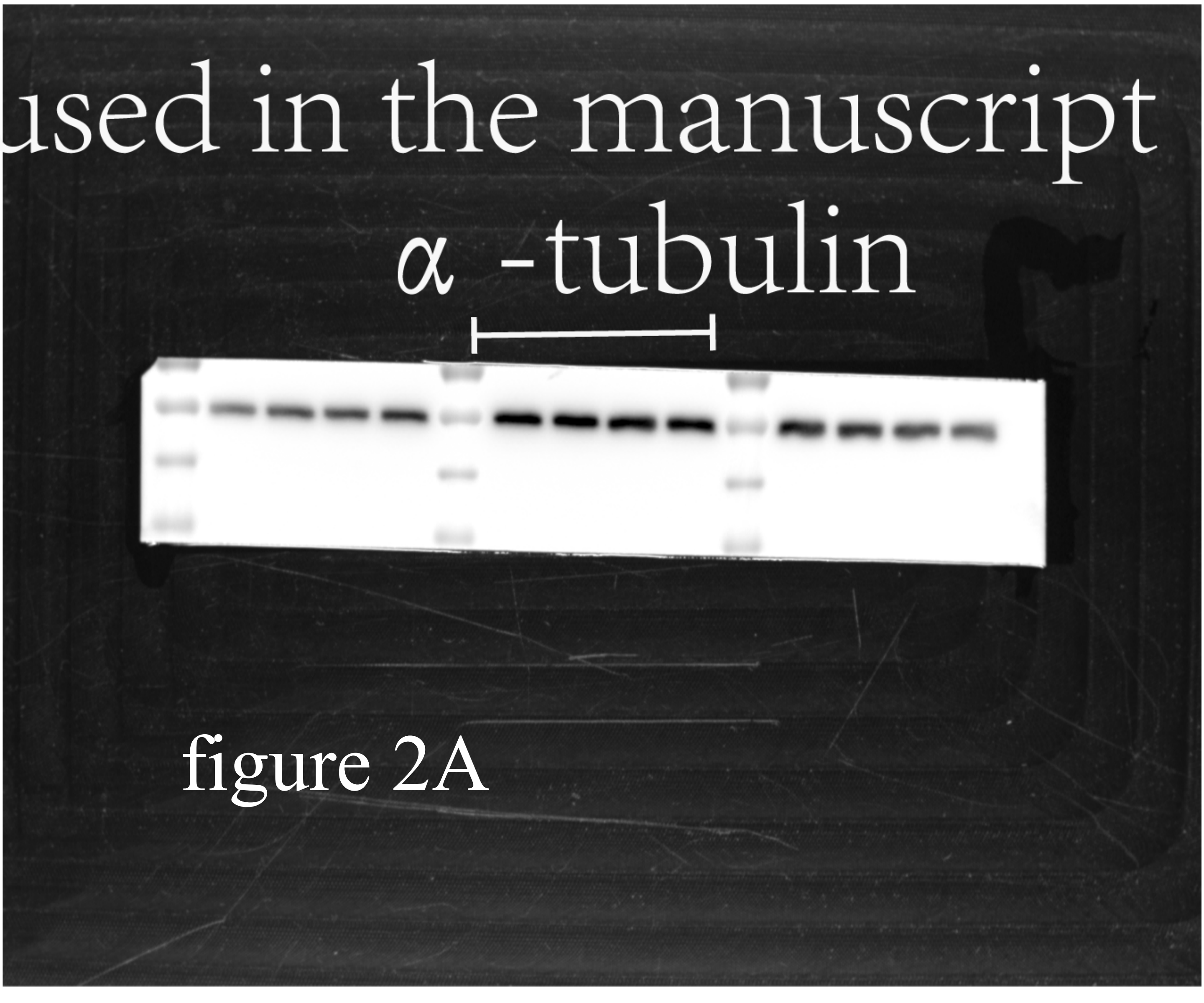
used in the manuscript
α -tubulin
figure 2A

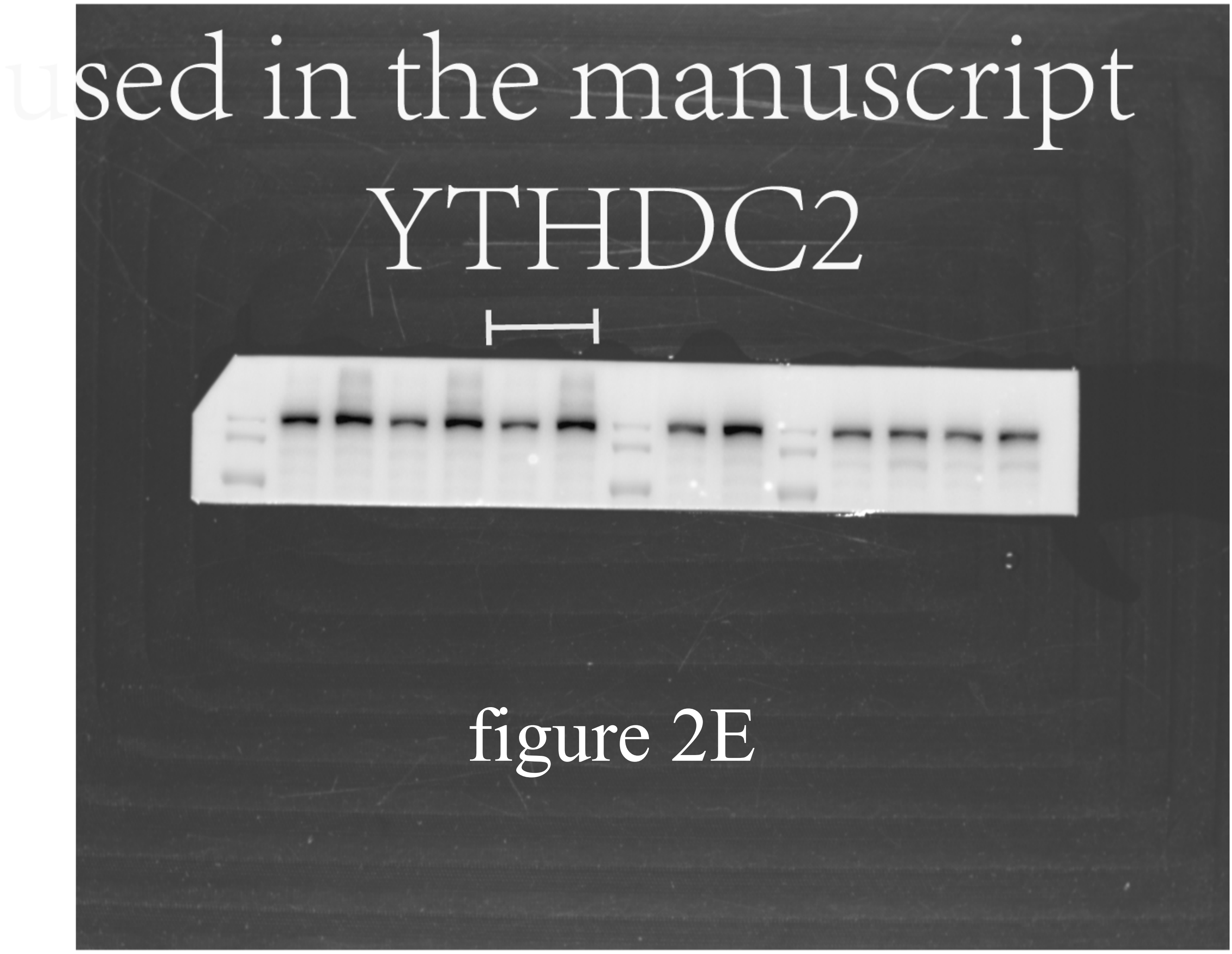
used in the manuscript
YTHDC2
figure 2E

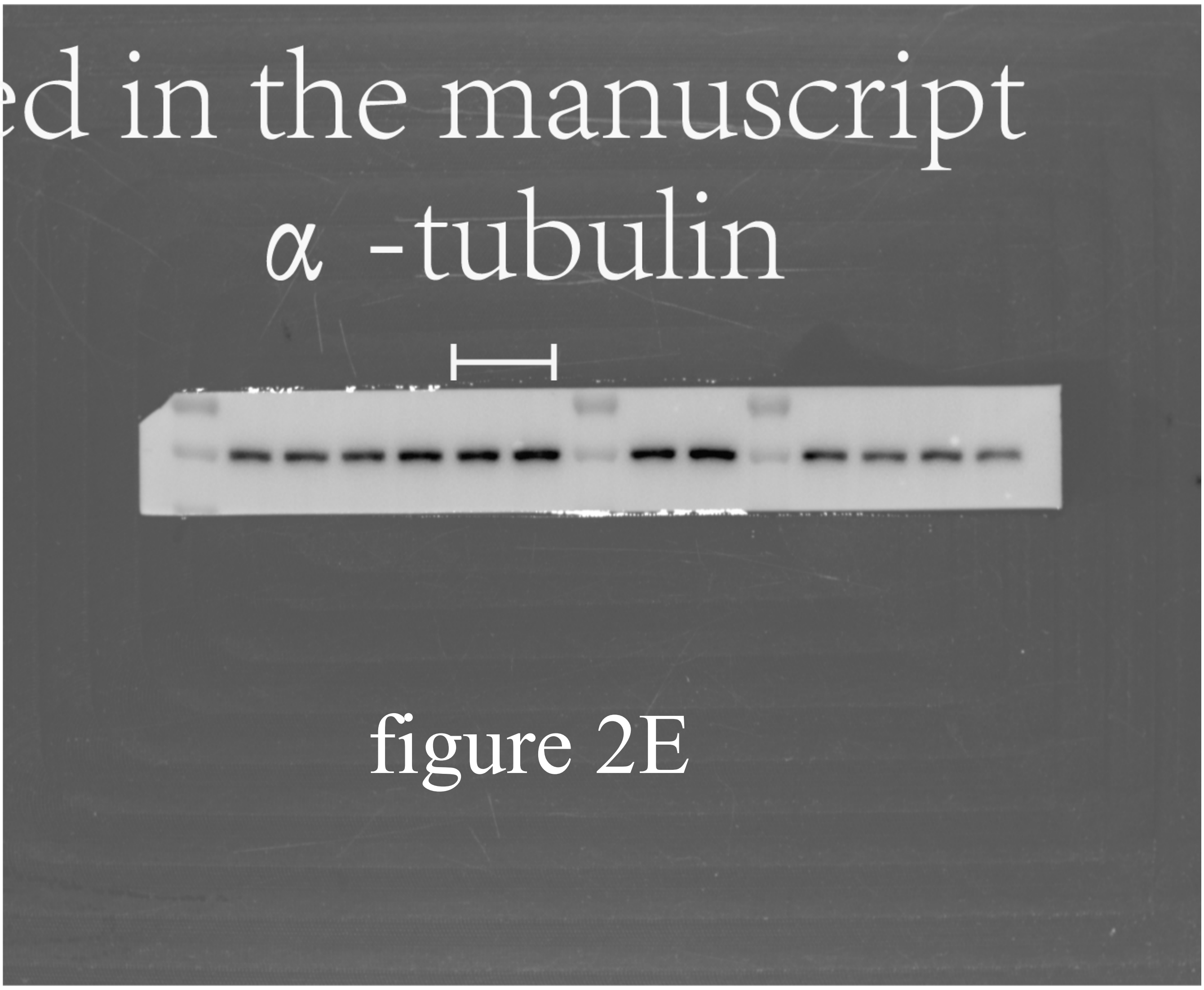
used in the manuscript
α -tubulin
figure 2E

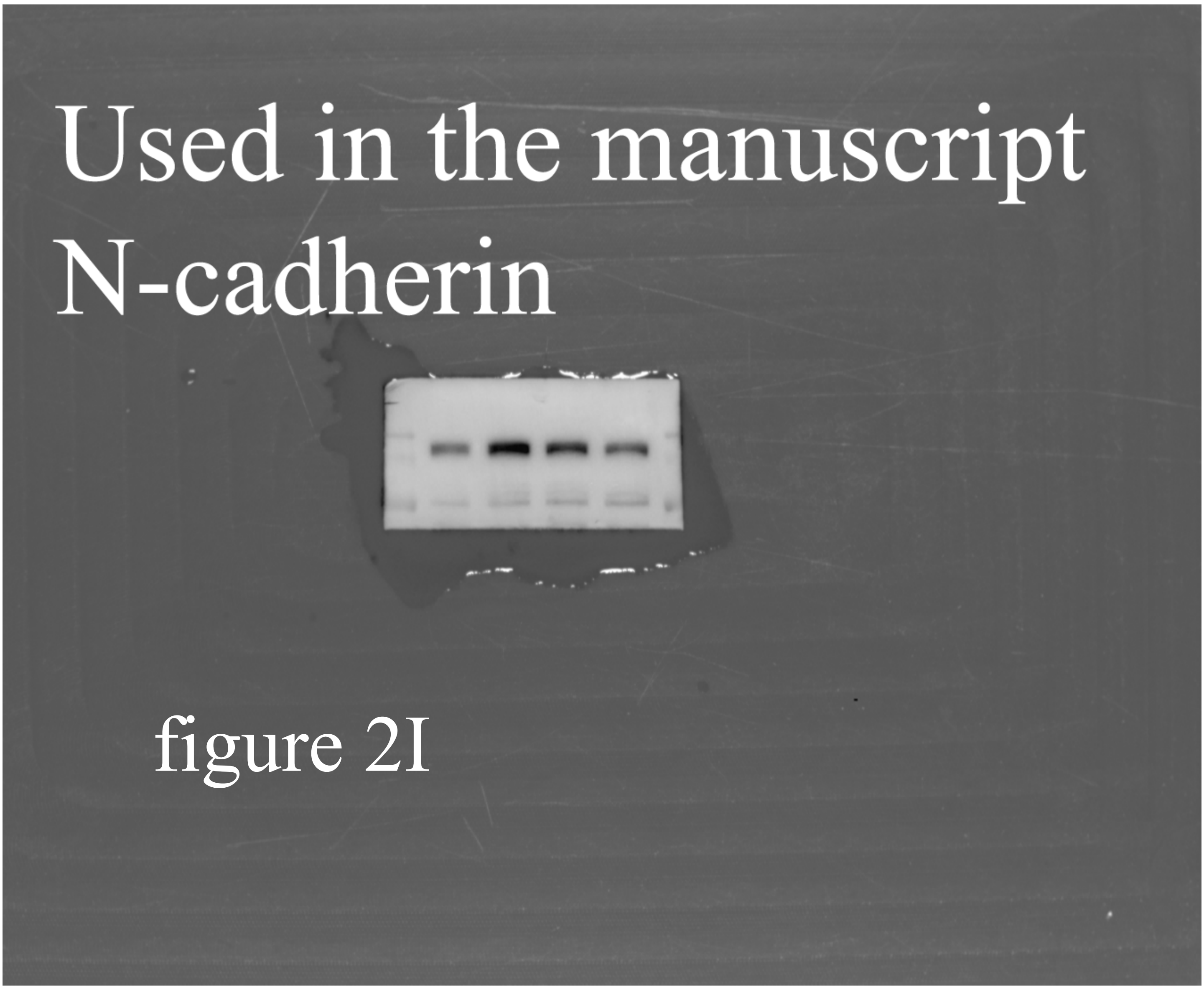
Used in the manuscript
N-cadherin
figure 2I

Used in the manuscript
E-cadherin

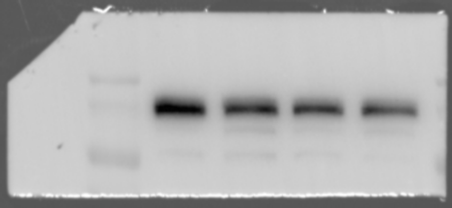

figure 2I

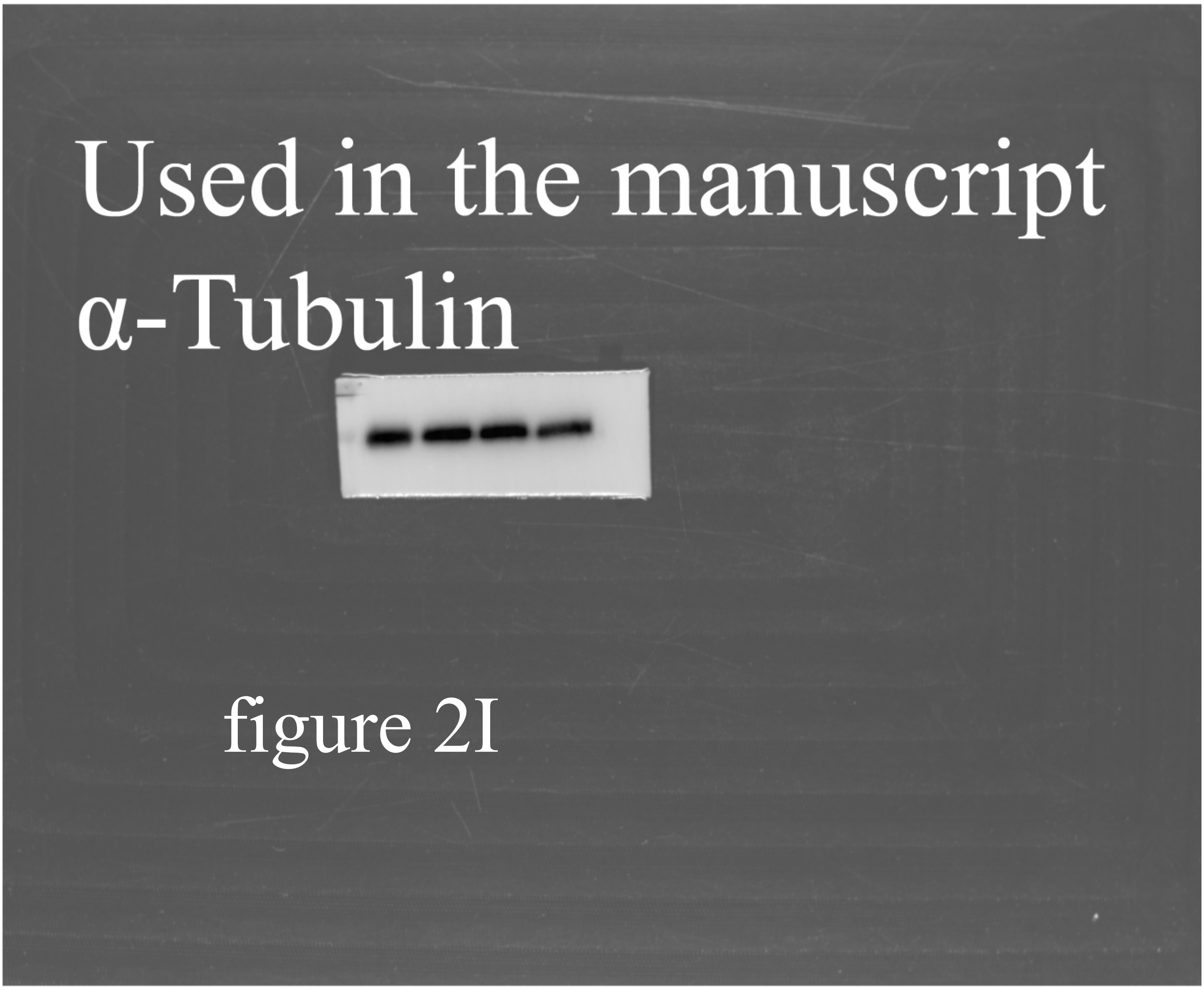
Used in the manuscript
α-Tubulin
figure 2I

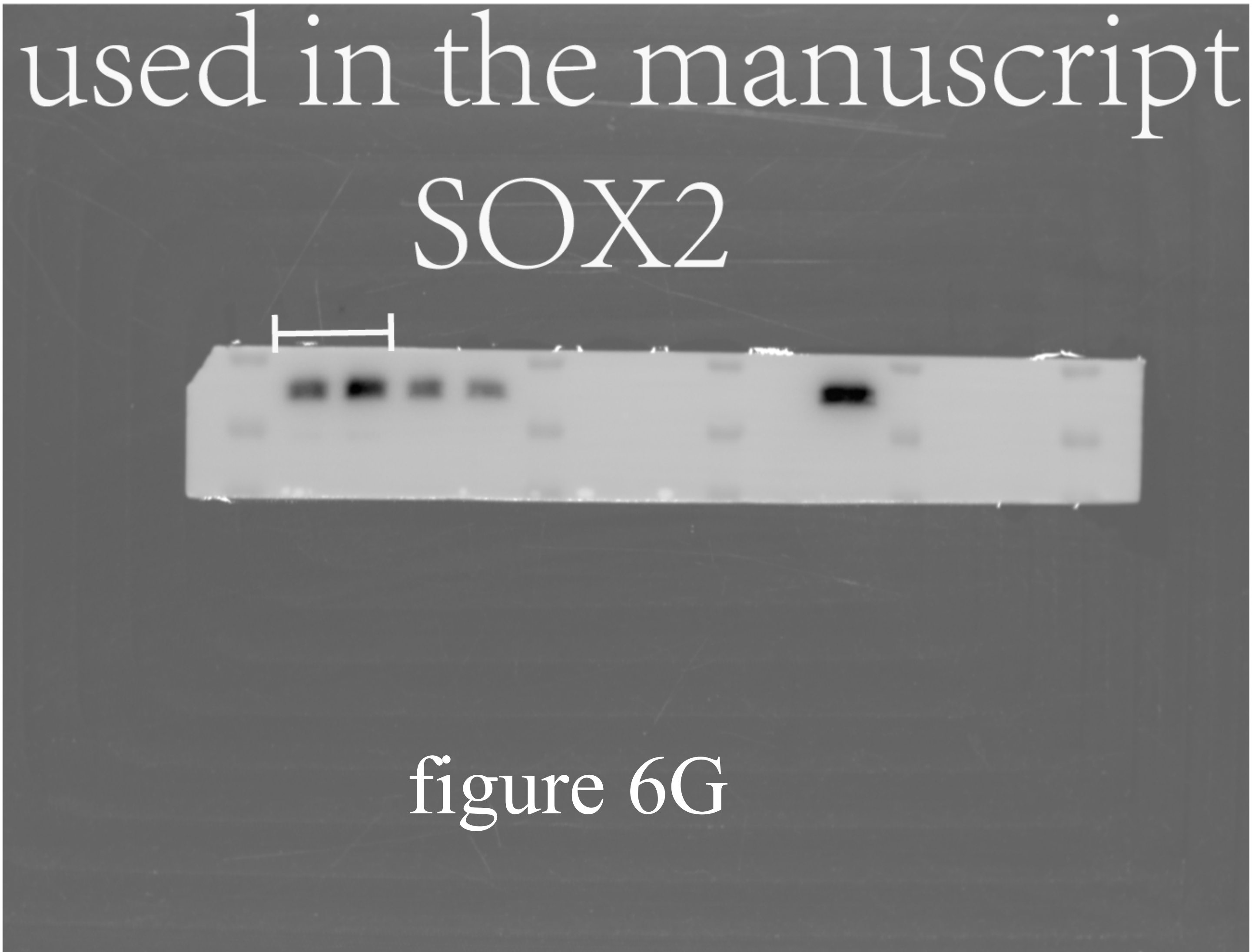
used in the manuscript
SOX2
figure 6G

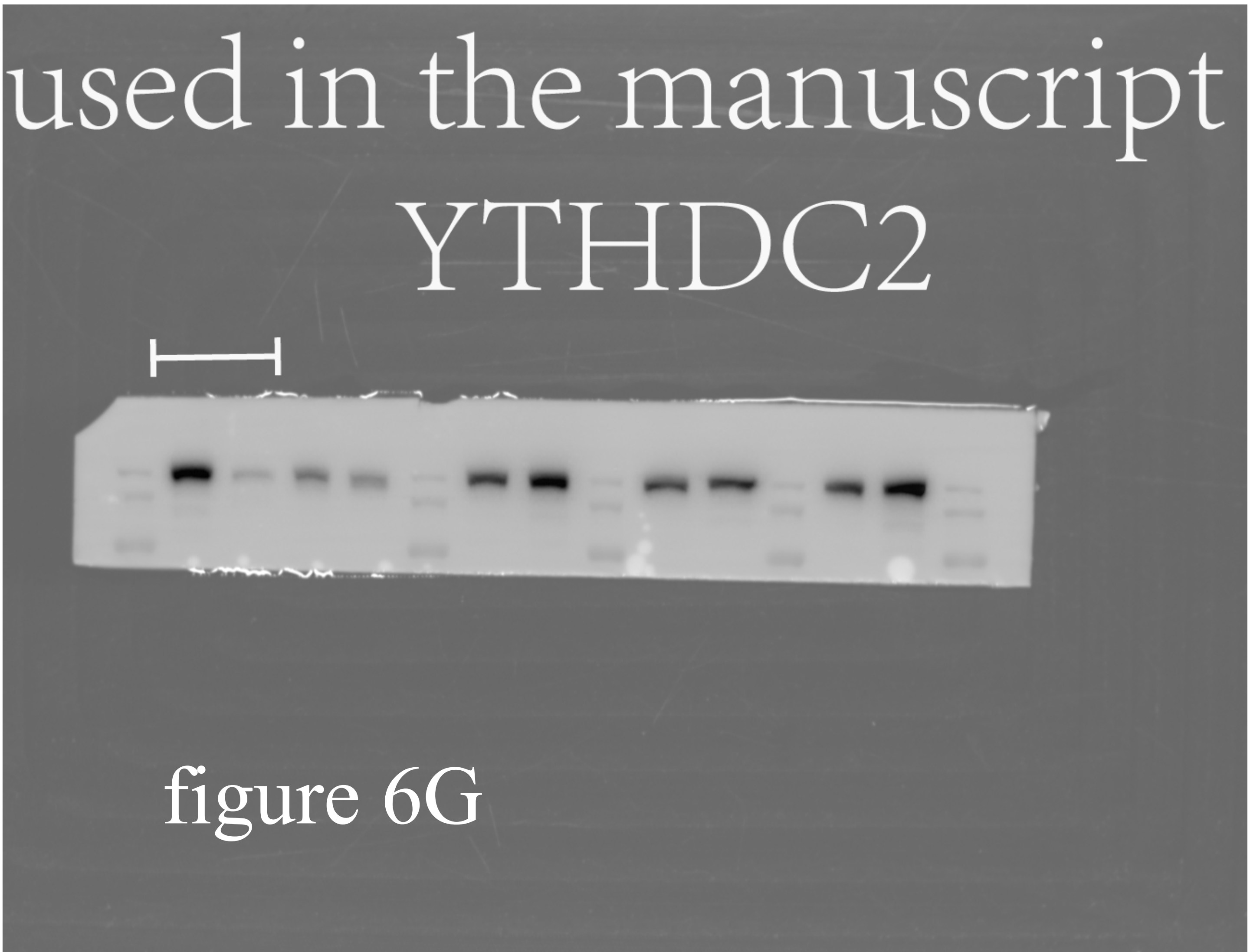
used in the manuscript
YTHDC2
figure 6G

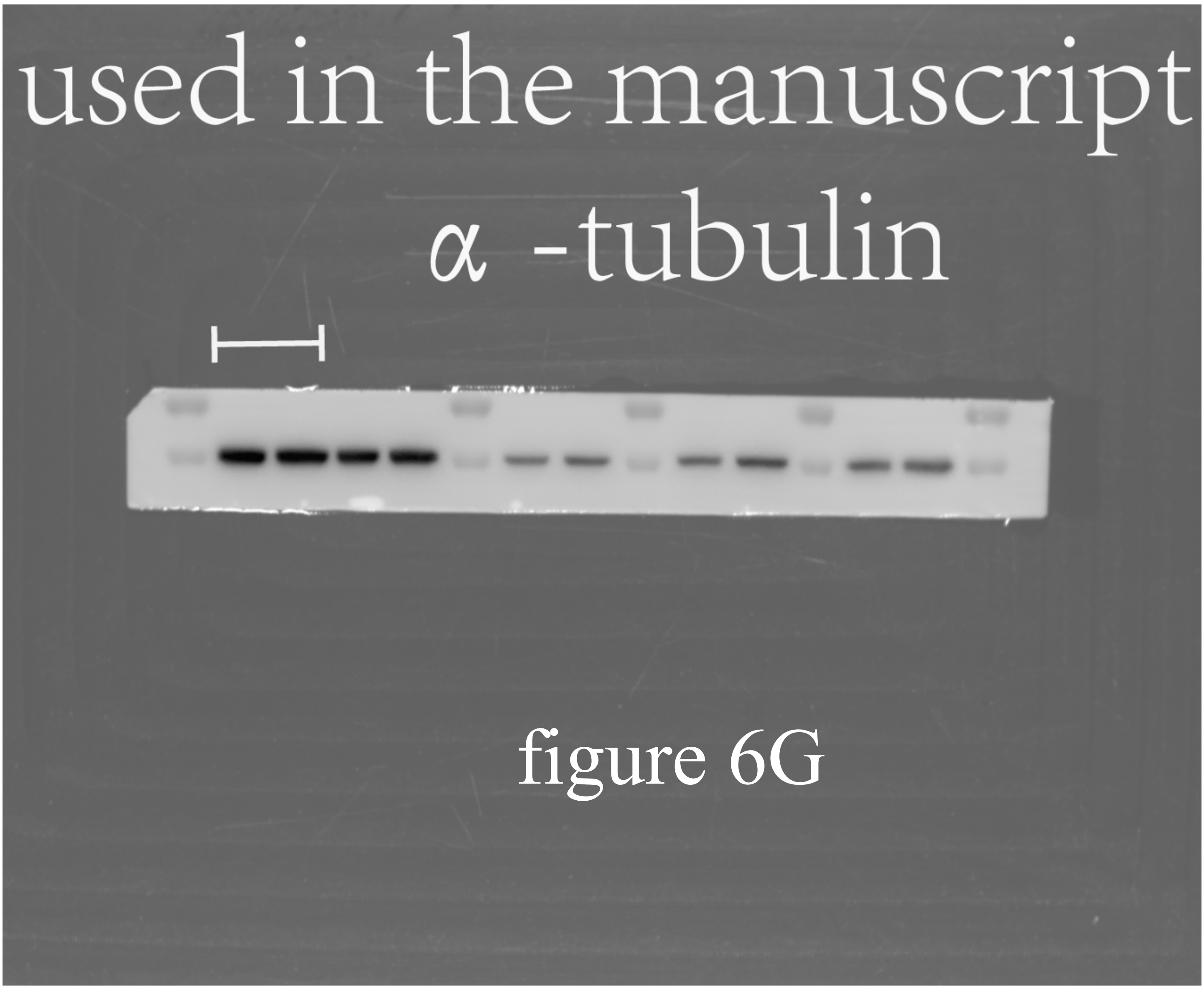
used in the manuscript
α -tubulin
figure 6G

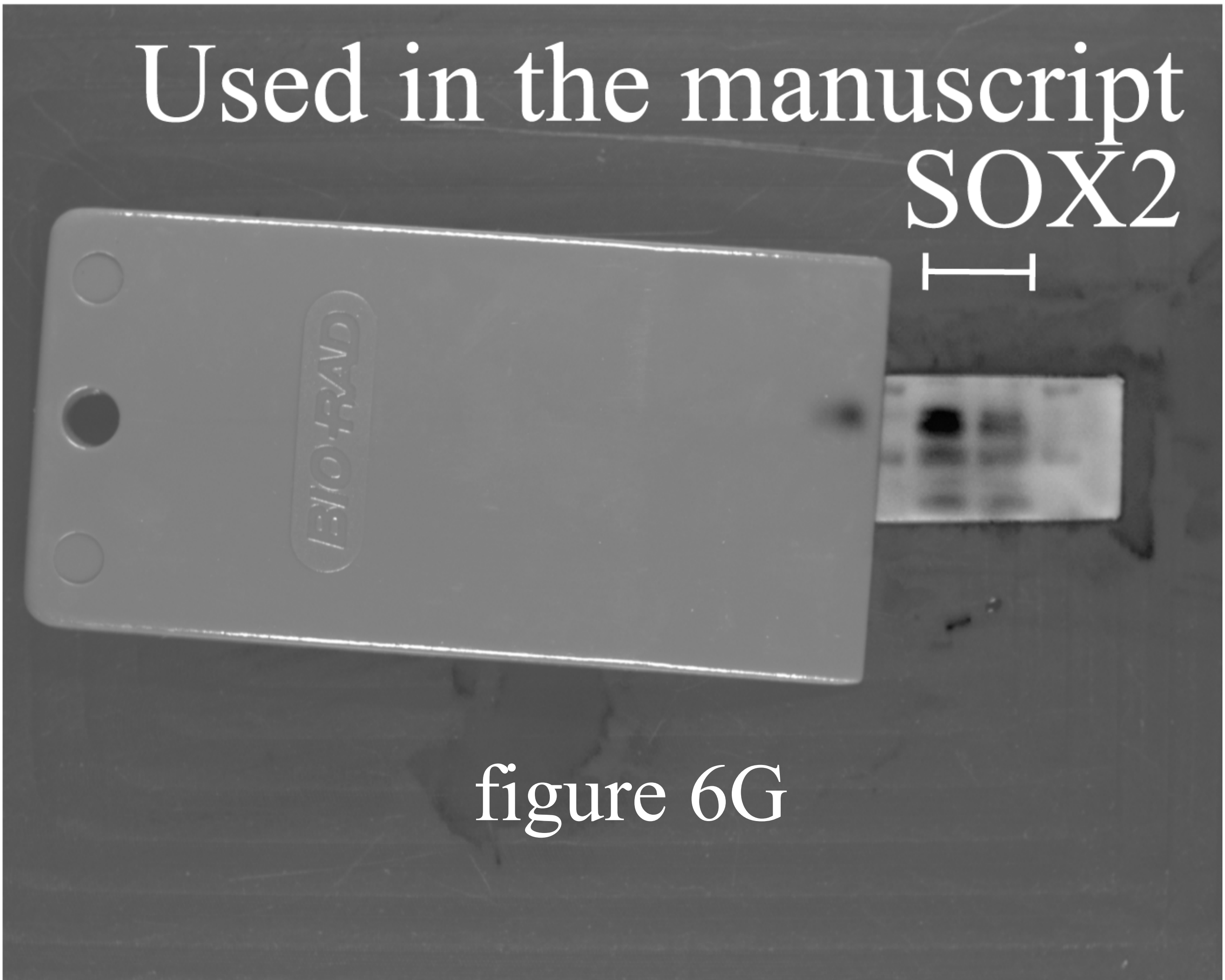
Used in the manuscript
SOX2
figure 6G

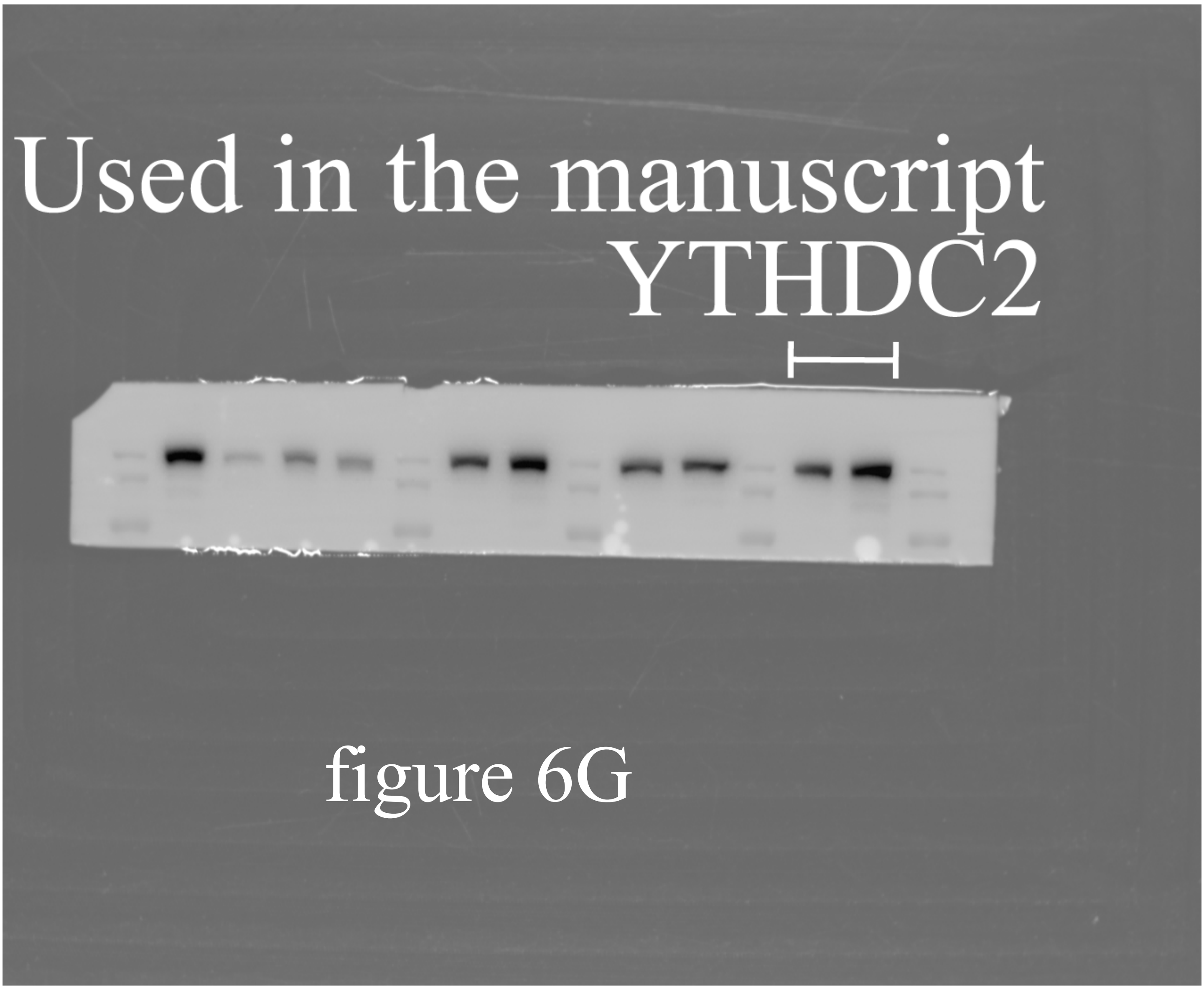
Used in the manuscript
YTHDC2
figure 6G

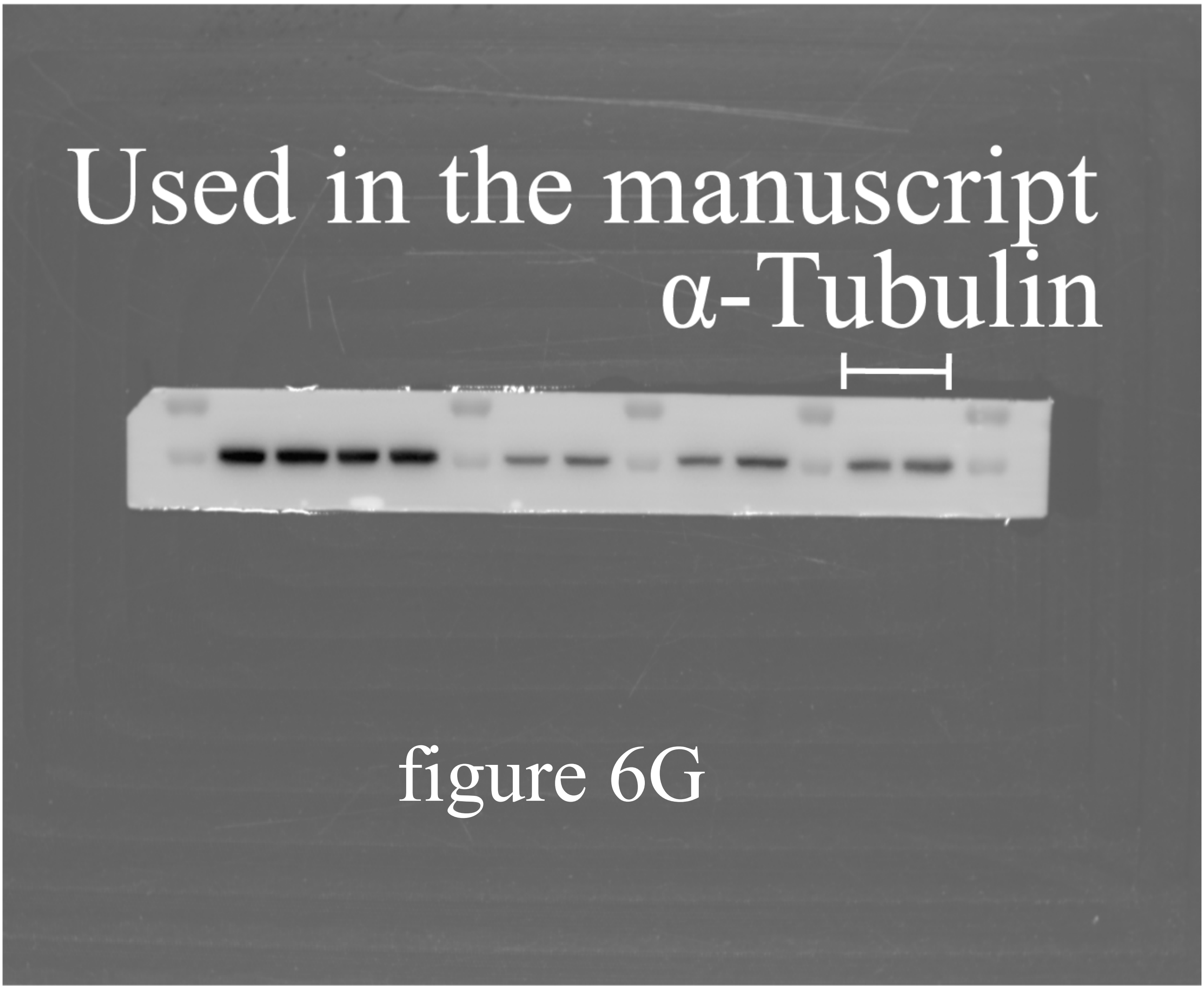
Used in the manuscript
α-Tubulin
figure 6G

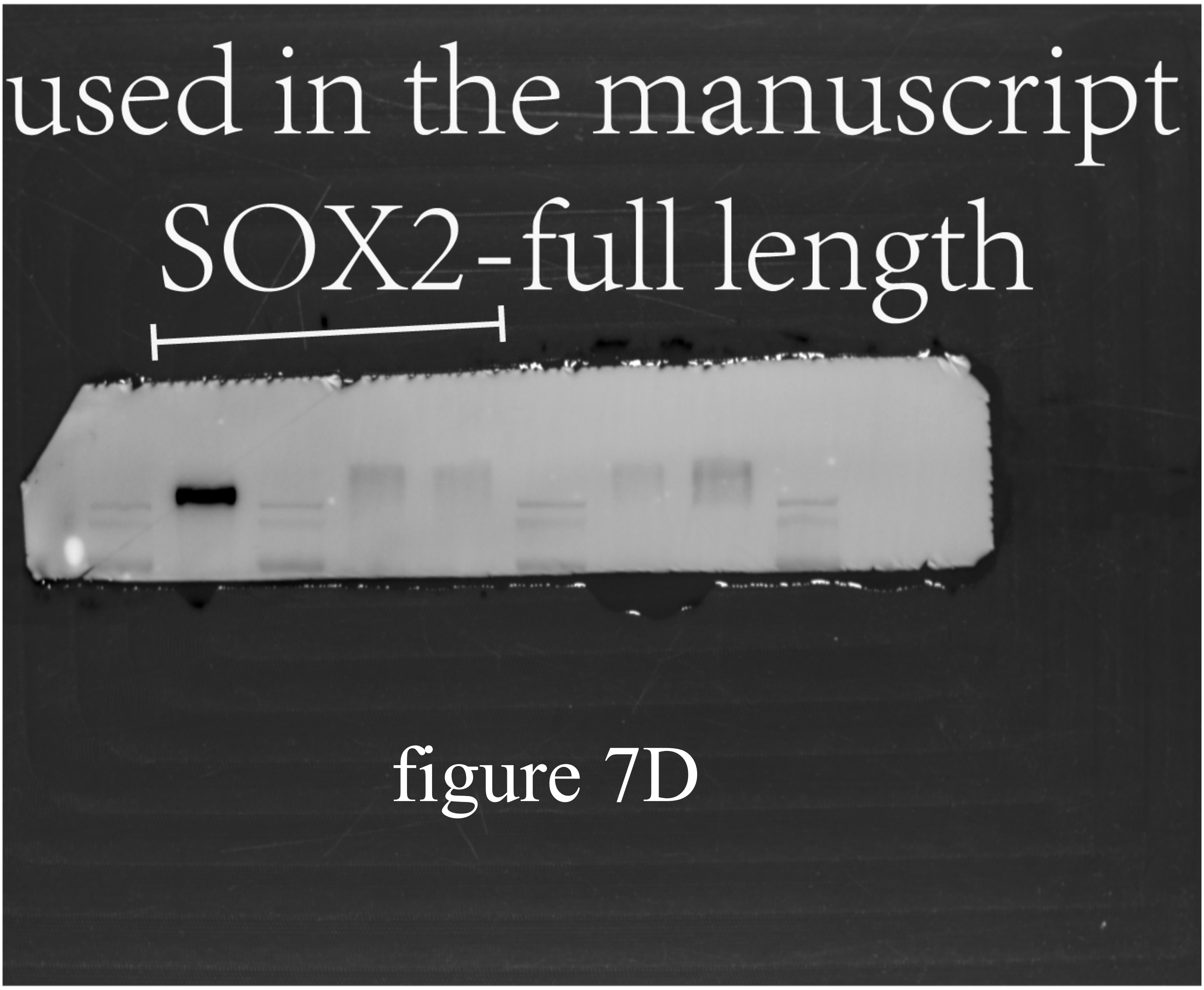
used in the manuscript
SOX2-full length
figure 7D

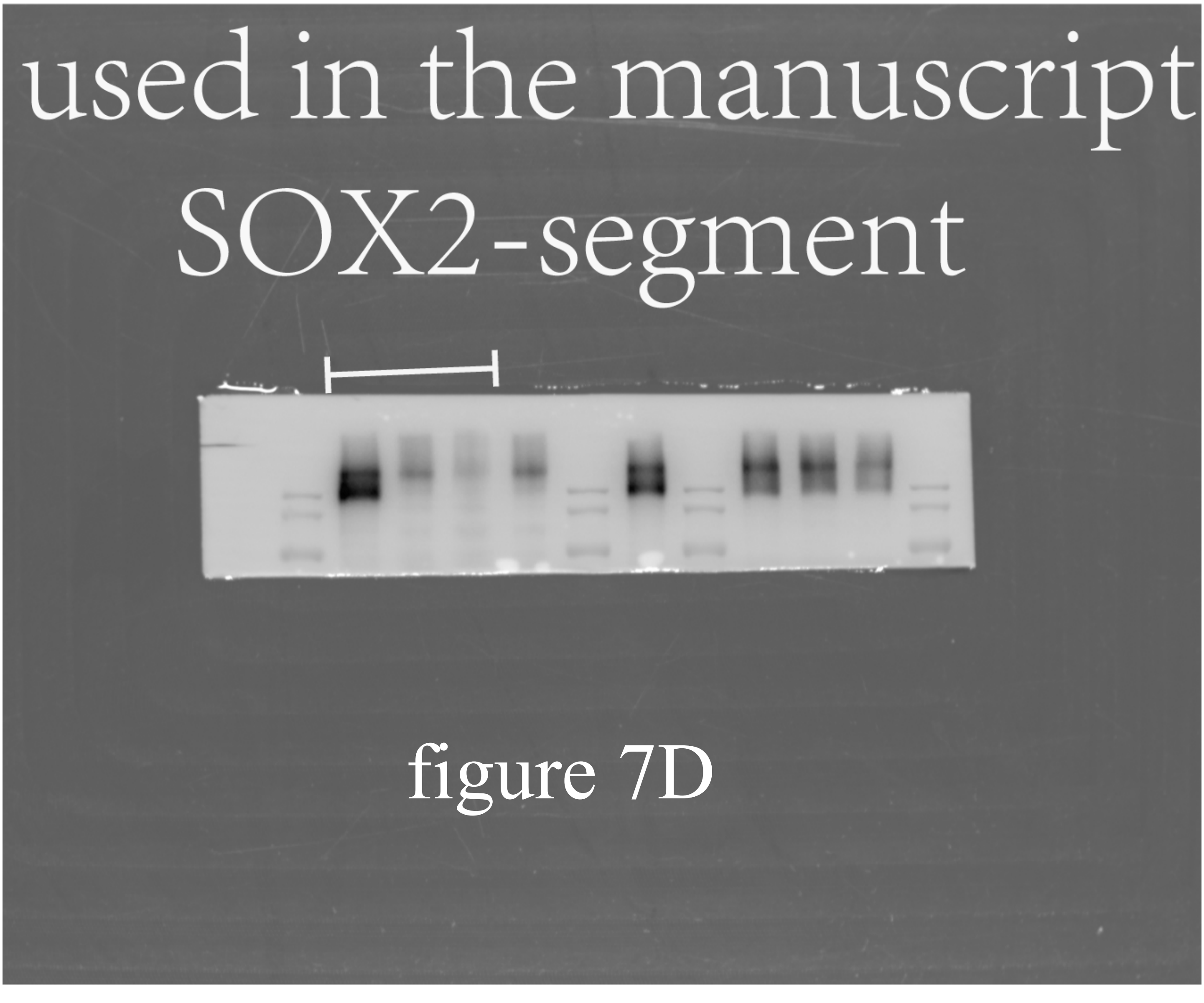
used in the manuscript
SOX2-segment
figure 7D

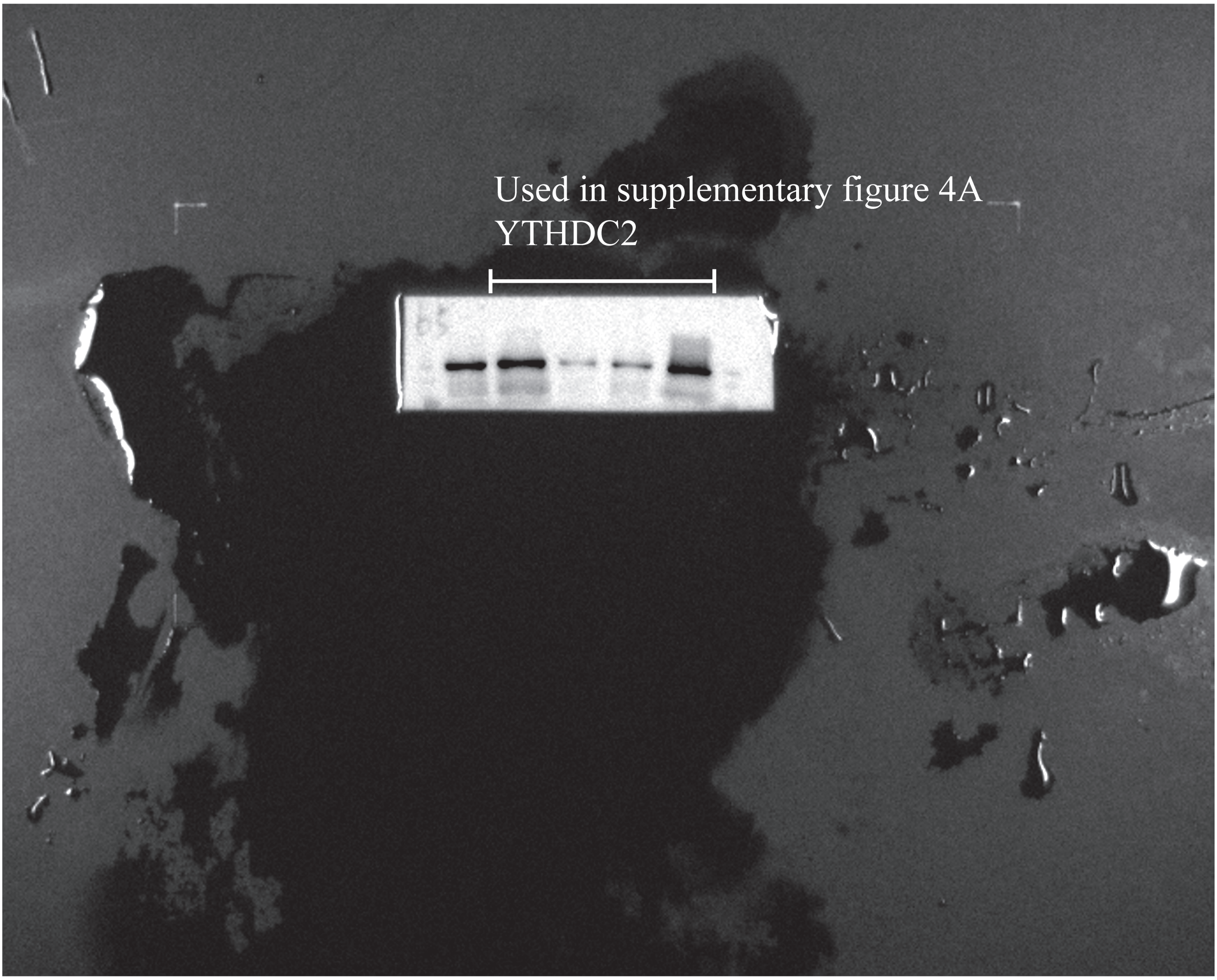
Used in supplementary figure 4A
YTHDC2

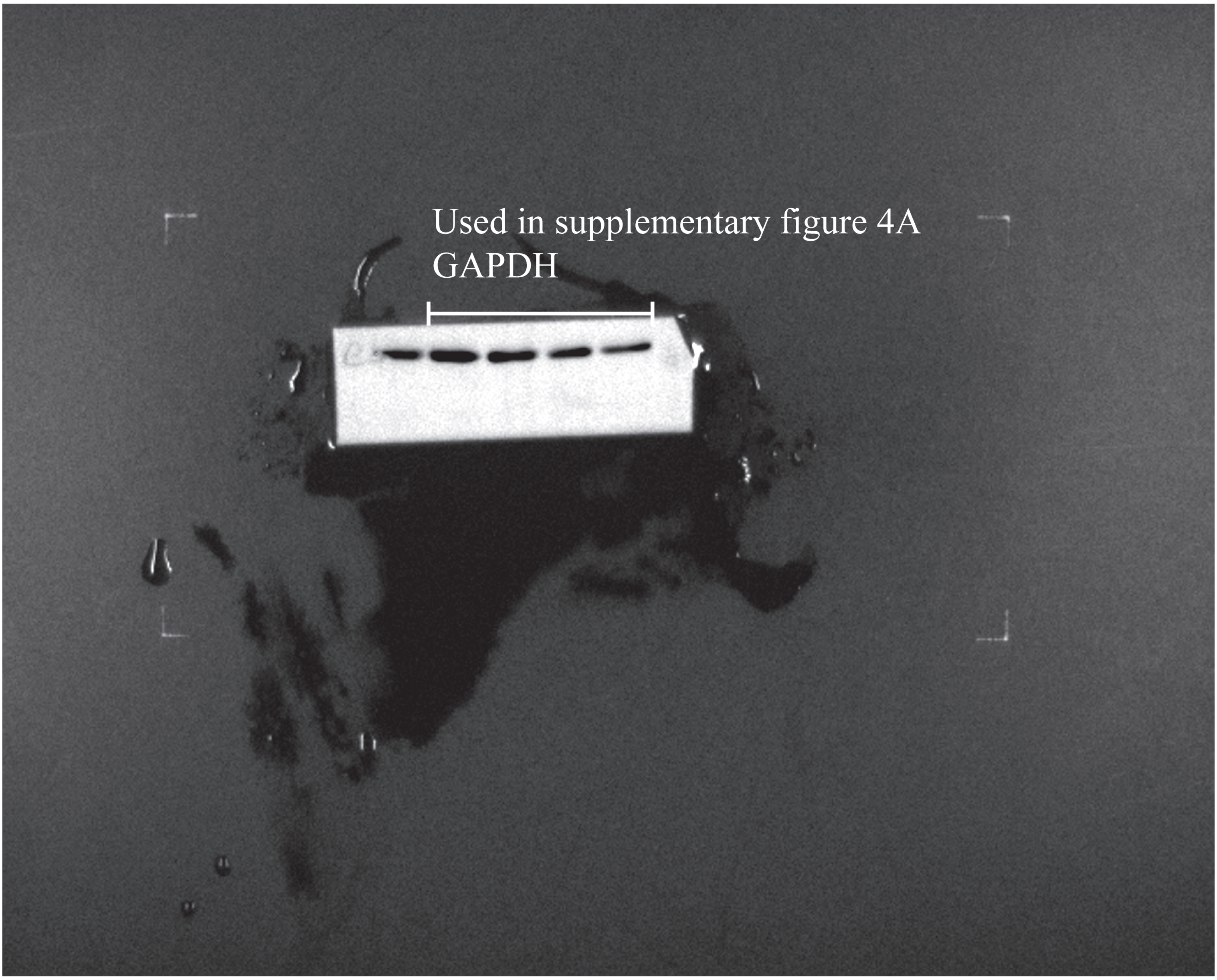
Used in supplementary figure 4A
GAPDH
