# Supplementary material for: YTHDC2 suppresses bladder cancer by inhibiting SOX2-mediated tumor plasticity
Source: Cell Death Dis. 2025 Oct 27;16(1):765. doi: 10.1038/s41419-025-08079-w (PMC12559364; doi:10.1038/s41419-025-08079-w)

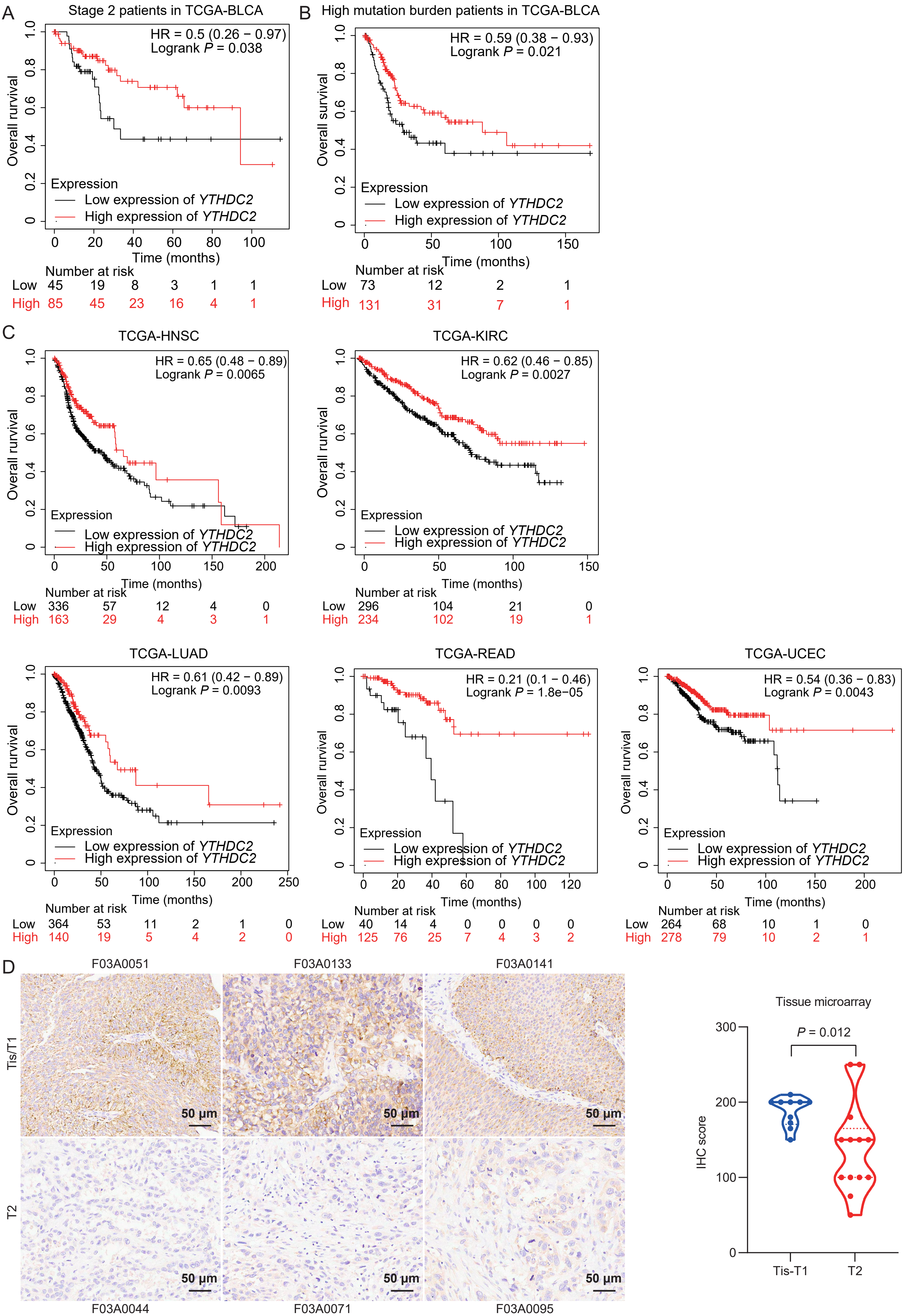
A
Stage 2 patients in TCGA-BLCA
HR = 0.5 (0.26 − 0.97)
Logrank P = 0.038
Overall rurvival
Expression
Low expression of YTHDC2
High expression of YTHDC2
Time (months)
Number at risk
Low 45 19 8 3 1 1
High 85 45 23 16 4 1
B
High mutation burden patients in TCGA-BLCA
HR = 0.59 (0.38 − 0.93)
Logrank P = 0.021
Overall survival
Expression
Low expression of YTHDC2
High expression of YTHDC2
Time (months)
Number at risk
Low 73 12 2 1
High 131 31 7 1
C
TCGA-HNSC
HR = 0.65 (0.48 − 0.89)
Logrank P = 0.0065
Overall rurvival
Expression
Low expression of YTHDC2
High expression of YTHDC2
Time (months)
Number at risk
Low 336 57 12 4 0
High 163 29 4 3 1
TCGA-KIRC
HR = 0.62 (0.46 − 0.85)
Logrank P = 0.0027
Overall rurvival
Expression
Low expression of YTHDC2
High expression of YTHDC2
Time (months)
Number at risk
Low 296 104 21 0
High 234 102 19 1
TCGA-LUAD
HR = 0.61 (0.42 − 0.89)
Logrank P = 0.0093
Overall rurvival
Expression
Low expression of YTHDC2
High expression of YTHDC2
Time (months)
Number at risk
Low 364 53 11 2 1 0
High 140 19 5 4 2 0
TCGA-READ
HR = 0.21 (0.1 − 0.46)
Logrank P = 1.8e−05
Overall rurvival
Expression
Low expression of YTHDC2
High expression of YTHDC2
Time (months)
Number at risk
Low 40 14 4 0 0 0 0
High 125 76 25 7 4 3 2
TCGA-UCEC
HR = 0.54 (0.36 − 0.83)
Logrank P = 0.0043
Overall rurvival
Expression
Low expression of YTHDC2
High expression of YTHDC2
Time (months)
Number at risk
Low 264 68 10 1 0
High 278 79 10 2 1
D
F03A0051
F03A0133
F03A0141
Tis/T1
T2
50 μm
F03A0044
F03A0071
F03A0095
Tissue microarray
P = 0.012
IHC score
Tis-T1
T2

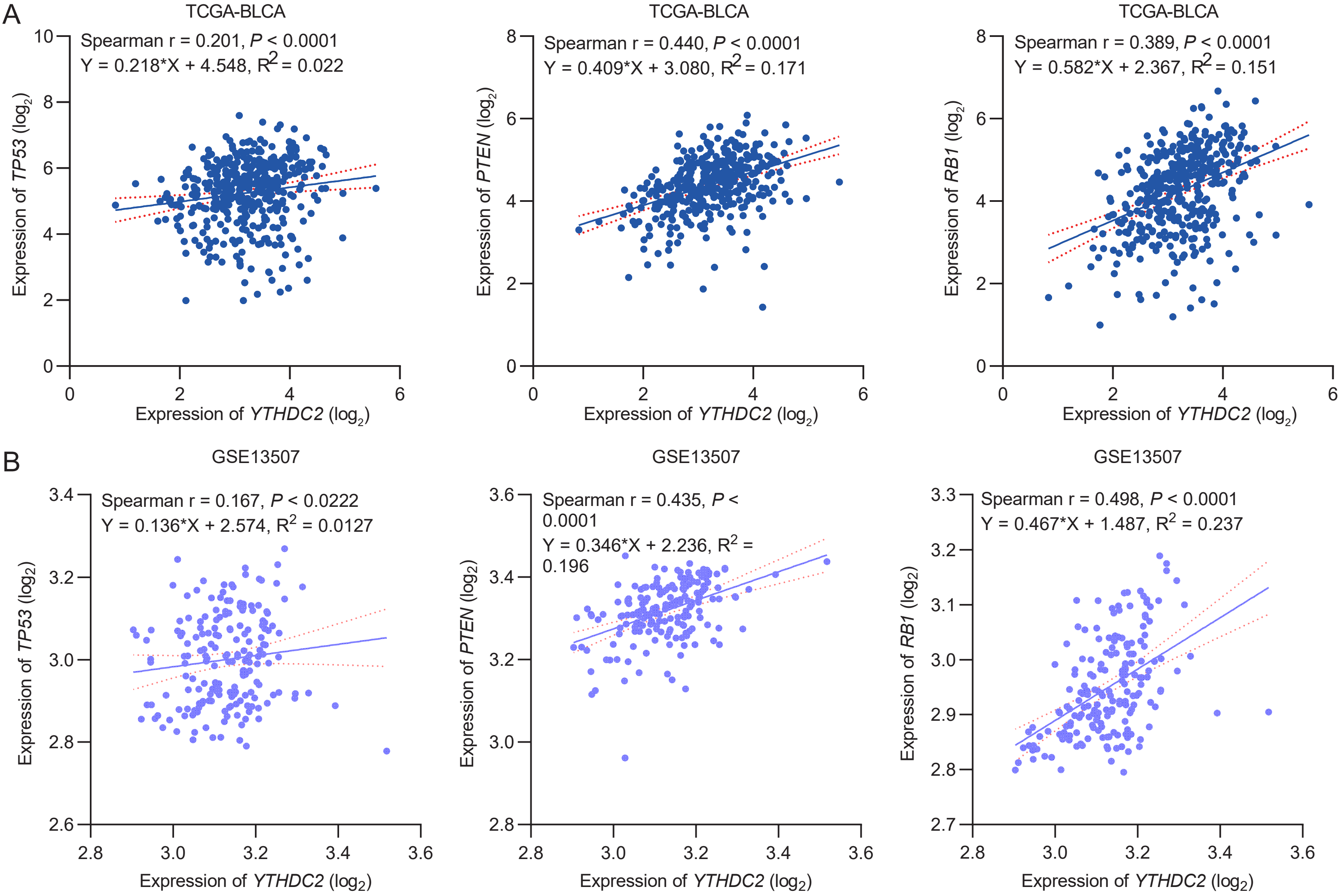
A
TCGA-BLCA
Spearman r = 0.201, P < 0.0001
Y = 0.218*X + 4.548, R2 = 0.022
Expression of TP53 (log2)
Expression of YTHDC2 (log2)
TCGA-BLCA
Spearman r = 0.440, P < 0.0001
Y = 0.409*X + 3.080, R2 = 0.171
Expression of PTEN (log2)
Expression of YTHDC2 (log2)
TCGA-BLCA
Spearman r = 0.389, P < 0.0001
Y = 0.582*X + 2.367, R2 = 0.151
Expression of RB1 (log2)
Expression of YTHDC2 (log2)
B
GSE13507
Spearman r = 0.167, P < 0.0222
Y = 0.136*X + 2.574, R2 = 0.0127
Expression of TP53 (log2)
Expression of YTHDC2 (log2)
GSE13507
Spearman r = 0.435, P < 0.0001
Y = 0.346*X + 2.236, R2 = 0.196
Expression of PTEN (log2)
Expression of YTHDC2 (log2)
GSE13507
Spearman r = 0.498, P < 0.0001
Y = 0.467*X + 1.487, R2 = 0.237
Expression of RB1 (log2)
Expression of YTHDC2 (log2)

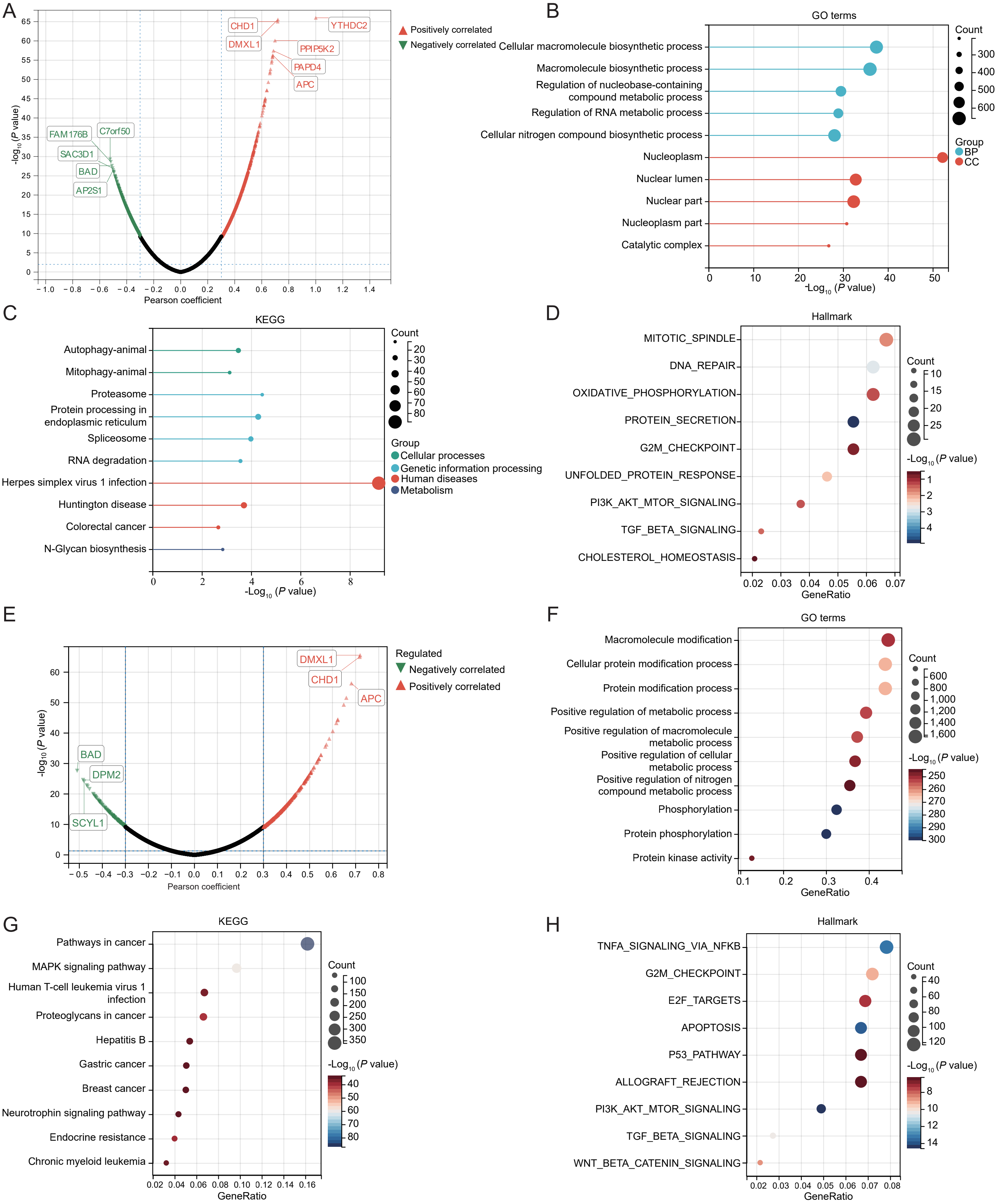
A
Positively correlated
Negatively correlated
CHD1
YTHDC2
DMXL1
PPIP5K2
PAPD4
APC
FAM176B
C7orf50
SAC3D1
BAD
AP2S1
-log10 (P value)
Pearson coefficient
B
GO terms
Cellular macromolecule biosynthetic process
Macromolecule biosynthetic process
Regulation of nucleobase-containing compound metabolic process
Regulation of RNA metabolic process
Cellular nitrogen compound biosynthetic process
Nucleoplasm
Nuclear lumen
Nuclear part
Nucleoplasm part
Catalytic complex
-Log10 (P value)
Count
Group
BP
CC
C
KEGG
Autophagy-animal
Mitophagy-animal
Proteasome
Protein processing in endoplasmic reticulum
Spliceosome
RNA degradation
Herpes simplex virus 1 infection
Huntington disease
Colorectal cancer
N-Glycan biosynthesis
-Log10 (P value)
Count
Group
Cellular processes
Genetic information processing
Human diseases
Metabolism
D
Hallmark
MITOTIC_SPINDLE
DNA_REPAIR
OXIDATIVE_PHOSPHORYLATION
PROTEIN_SECRETION
G2M_CHECKPOINT
UNFOLDED_PROTEIN_RESPONSE
PI3K_AKT_MTOR_SIGNALING
TGF_BETA_SIGNALING
CHOLESTEROL_HOMEOSTASIS
GeneRatio
Count
-Log10 (P value)
E
Regulated
Negatively correlated
Positively correlated
DMXL1
CHD1
APC
BAD
DPM2
SCYL1
-log10 (P value)
Pearson coefficient
F
GO terms
Macromolecule modification
Cellular protein modification process
Protein modification process
Positive regulation of metabolic process
Positive regulation of macromolecule metabolic process
Positive regulation of cellular metabolic process
Positive regulation of nitrogen compound metabolic process
Phosphorylation
Protein phosphorylation
Protein kinase activity
GeneRatio
Count
-Log10 (P value)
G
KEGG
Pathways in cancer
MAPK signaling pathway
Human T-cell leukemia virus 1 infection
Proteoglycans in cancer
Hepatitis B
Gastric cancer
Breast cancer
Neurotrophin signaling pathway
Endocrine resistance
Chronic myeloid leukemia
GeneRatio
Count
-Log10 (P value)
H
Hallmark
TNFA_SIGNALING_VIA_NFKB
G2M_CHECKPOINT
E2F_TARGETS
APOPTOSIS
P53_PATHWAY
ALLOGRAFT_REJECTION
PI3K_AKT_MTOR_SIGNALING
TGF_BETA_SIGNALING
WNT_BETA_CATENIN_SIGNALING
GeneRatio
Count
-Log10 (P value)

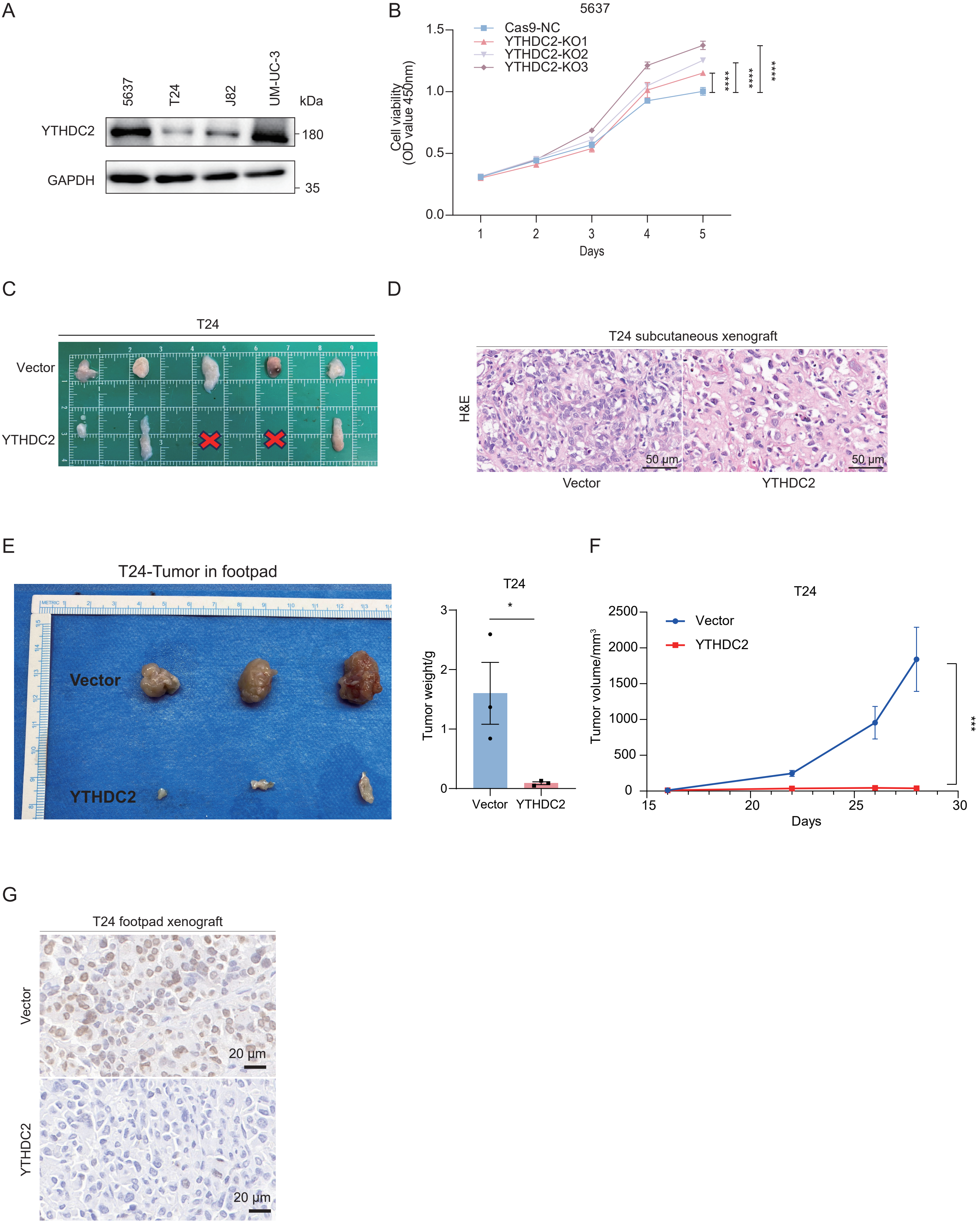

A
5637
T24
J82
UM-UC-3
kDa
YTHDC2
180
GAPDH
35
B
5637
Cas9-NC
YTHDC2-KO1
YTHDC2-KO2
YTHDC2-KO3
Cell viability (OD value 450nm)
Days
****
****
****
C
T24
Vector
YTHDC2
D
T24 subcutaneous xenograft
H&E
50 μm
50 μm
Vector
YTHDC2
E
T24-Tumor in footpad
Vector
YTHDC2
T24
Tumor weight/g
Vector
YTHDC2
*
F
T24
Vector
YTHDC2
Tumor volume/mm3
Days
***
G
T24 footpad xenograft
Vector
YTHDC2
20 μm
20 μm

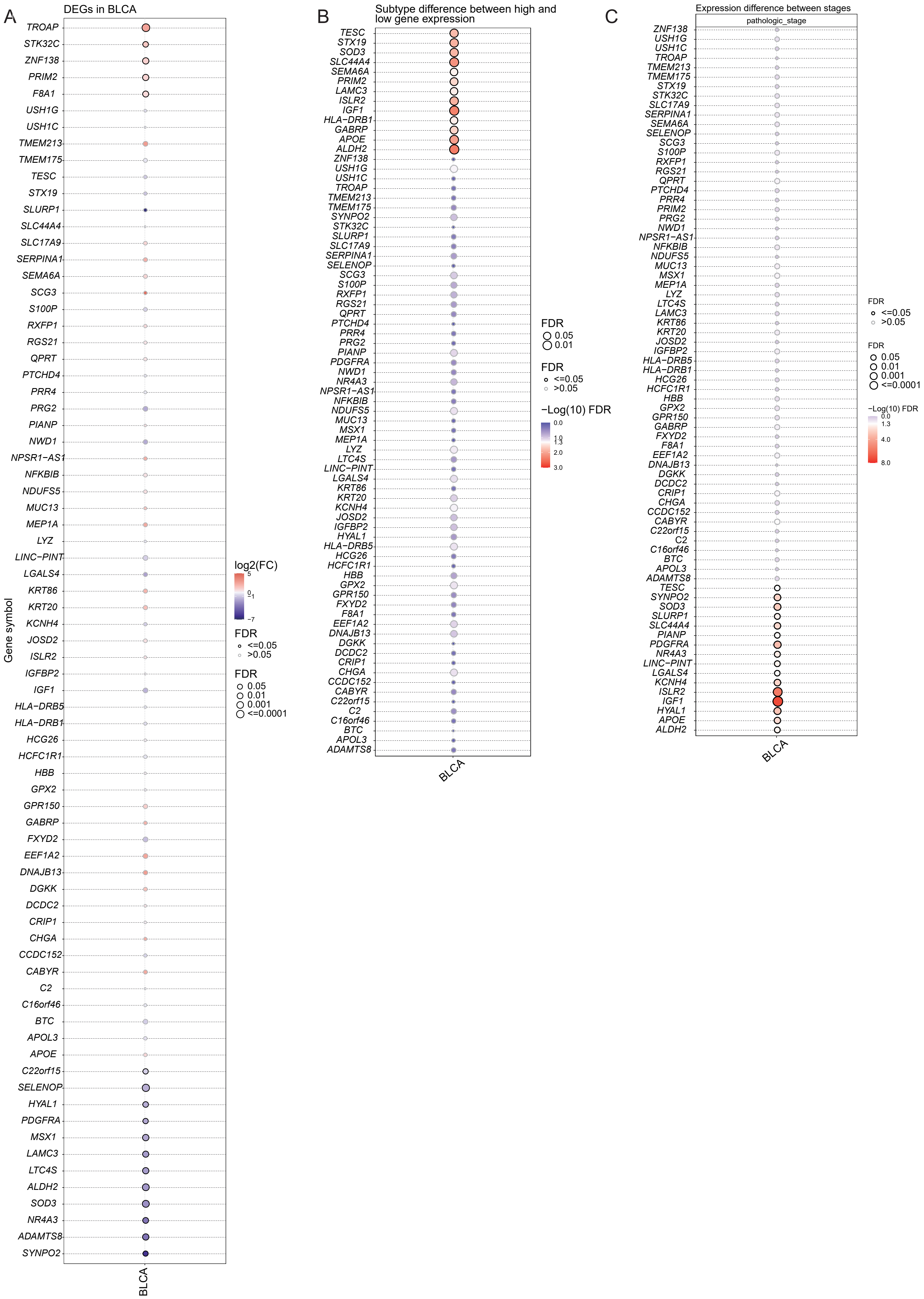
A
DEGs in BLCA
TROAP
STK32C
ZNF138
PRIM2
F8A1
USH1G
USH1C
TMEM213
TMEM175
TESC
STX19
SLURP1
SLC44A4
SLC17A9
SERPINA1
SEMA6A
SCG3
S100P
RXFP1
RGS21
QPRT
PTCHD4
PRR4
PRG2
PIANP
NWD1
NPSR1−AS1
NFKBIB
NDUFS5
MUC13
MEP1A
LYZ
LINC−PINT
LGALS4
KRT86
KRT20
KCNH4
JOSD2
ISLR2
IGFBP2
IGF1
HLA−DRB5
HLA−DRB1
HCG26
HCFC1R1
HBB
GPX2
GPR150
GABRP
FXYD2
EEF1A2
DNAJB13
DGKK
DCDC2
CRIP1
CHGA
CCDC152
CABYR
C2
C16orf46
BTC
APOL3
APOE
C22orf15
SELENOP
HYAL1
PDGFRA
MSX1
LAMC3
LTC4S
ALDH2
SOD3
NR4A3
ADAMTS8
SYNPO2
Gene symbol
BLCA
log2(FC)
5
0
1
−1
−7
FDR
<=0.05
>0.05
FDR
0.05
0.01
0.001
<=0.0001
B
Subtype difference between high and low gene expression
TESC
STX19
SOD3
SLC44A4
SEMA6A
PRIM2
LAMC3
ISLR2
IGF1
HLA−DRB1
GABRP
APOE
ALDH2
ZNF138
USH1G
USH1C
TROAP
TMEM213
TMEM175
SYNPO2
STK32C
SLURP1
SLC17A9
SERPINA1
SELENOP
SCG3
S100P
RXFP1
RGS21
QPRT
PTCHD4
PRR4
PRG2
PIANP
PDGFRA
NWD1
NR4A3
NPSR1−AS1
NFKBIB
NDUFS5
MUC13
MSX1
MEP1A
LYZ
LTC4S
LINC−PINT
LGALS4
KRT86
KRT20
KCNH4
JOSD2
IGFBP2
HYAL1
HLA−DRB5
HCG26
HCFC1R1
HBB
GPX2
GPR150
FXYD2
F8A1
EEF1A2
DNAJB13
DGKK
DCDC2
CRIP1
CHGA
CCDC152
CABYR
C22orf15
C2
C16orf46
BTC
APOL3
ADAMTS8
BLCA
FDR
0.05
0.01
FDR
<=0.05
>0.05
−Log(10) FDR
0.0
1.0
1.3
2.0
3.0
C
Expression difference between stages
pathologic_stage
ZNF138
USH1G
USH1C
TROAP
TMEM213
TMEM175
STX19
STK32C
SLC17A9
SERPINA1
SEMA6A
SELENOP
SCG3
S100P
RXFP1
RGS21
QPRT
PTCHD4
PRR4
PRIM2
PRG2
NWD1
NPSR1−AS1
NFKBIB
NDUFS5
MUC13
MSX1
MEP1A
LYZ
LTC4S
LAMC3
KRT86
KRT20
JOSD2
IGFBP2
HLA−DRB5
HLA−DRB1
HCG26
HCFC1R1
HBB
GPX2
GPR150
GABRP
FXYD2
F8A1
EEF1A2
DNAJB13
DGKK
DCDC2
CRIP1
CHGA
CCDC152
CABYR
C22orf15
C2
C16orf46
BTC
APOL3
ADAMTS8
TESC
SYNPO2
SOD3
SLURP1
SLC44A4
PIANP
PDGFRA
NR4A3
LINC−PINT
LGALS4
KCNH4
ISLR2
IGF1
HYAL1
APOE
ALDH2
BLCA
FDR
<=0.05
>0.05
FDR
0.05
0.01
0.001
<=0.0001
−Log(10) FDR
0.0
1.3
4.0
8.0

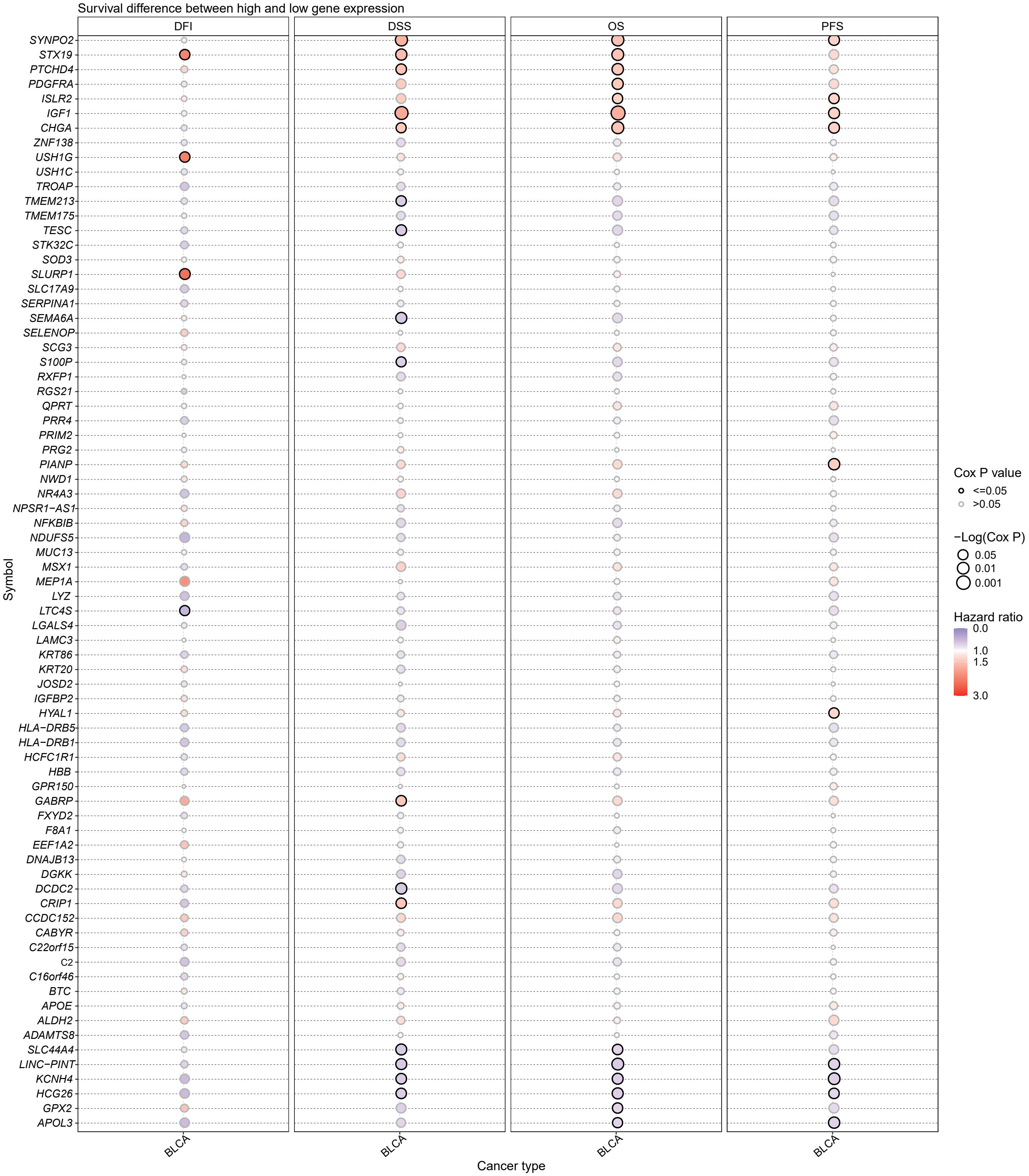

Survival difference between high and low gene expression
DFI
DSS
OS
PFS
Symbol
SYNPO2
STX19
PTCHD4
PDGFRA
ISLR2
IGF1
CHGA
ZNF138
USH1G
USH1C
TROAP
TMEM213
TMEM175
TESC
STK32C
SOD3
SLURP1
SLC17A9
SERPINA1
SEMA6A
SELENOP
SCG3
S100P
RXFP1
RGS21
QPRT
PRR4
PRIM2
PRG2
PIANP
NWD1
NR4A3
NPSR1−AS1
NFKBIB
NDUFS5
MUC13
MSX1
MEP1A
LYZ
LTC4S
LGALS4
LAMC3
KRT86
KRT20
JOSD2
IGFBP2
HYAL1
HLA−DRB5
HLA−DRB1
HCFC1R1
HBB
GPR150
GABRP
FXYD2
F8A1
EEF1A2
DNAJB13
DGKK
DCDC2
CRIP1
CCDC152
CABYR
C22orf15
C2
C16orf46
BTC
APOE
ALDH2
ADAMTS8
SLC44A4
LINC−PINT
KCNH4
HCG26
GPX2
APOL3
BLCA
BLCA
BLCA
BLCA
Cancer type
Cox P value
<=0.05
>0.05
−Log(Cox P)
0.05
0.01
0.001
Hazard ratio
0.0
1.0
1.5
3.0

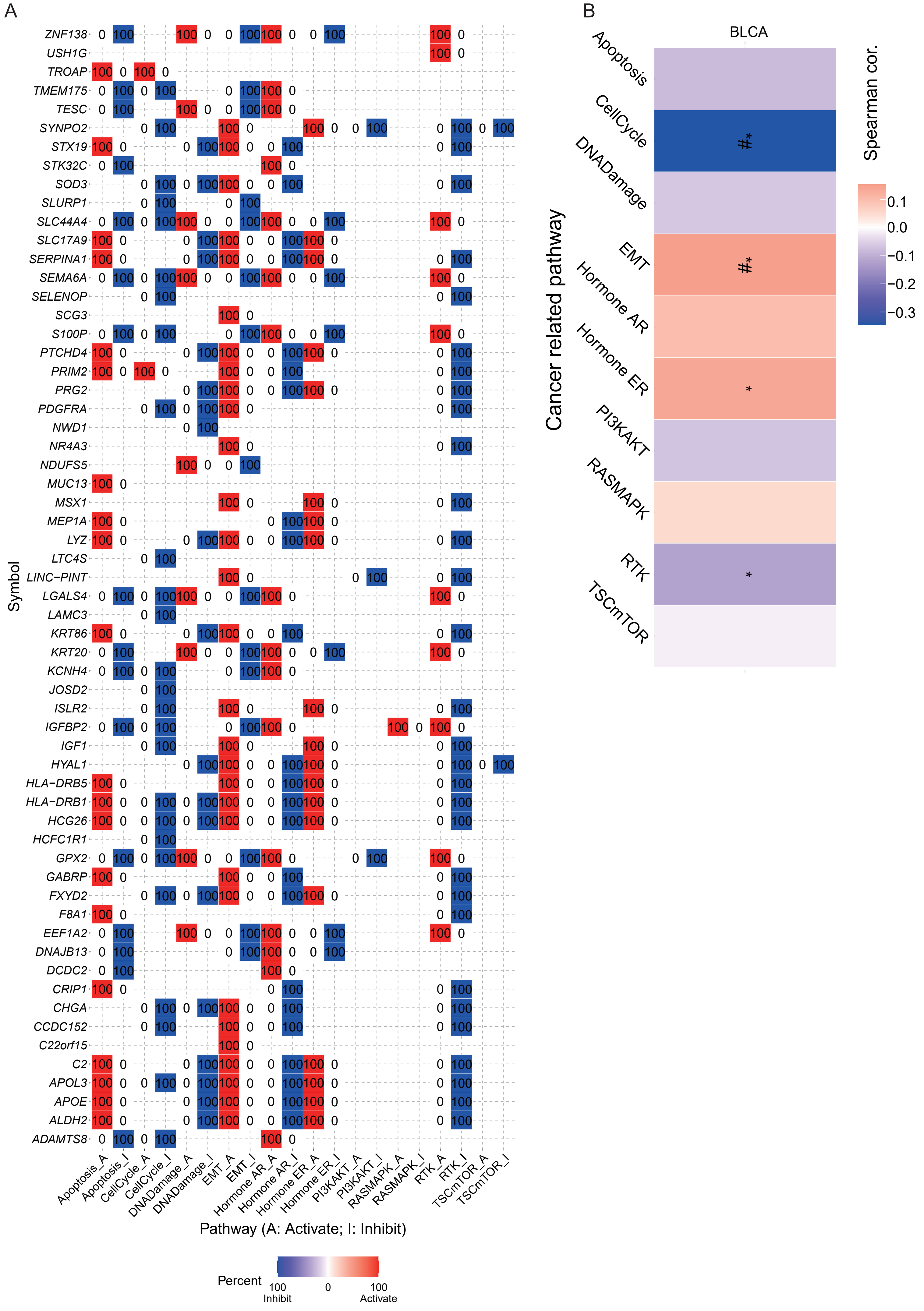
A
B
Symbol
ZNF138
USH1G
TROAP
TMEM175
TESC
SYNPO2
STX19
STK32C
SOD3
SLURP1
SLC44A4
SLC17A9
SERPINA1
SEMA6A
SELENOP
SCG3
S100P
PTCHD4
PRIM2
PRG2
PDGFRA
NWD1
NR4A3
NDUFS5
MUC13
MSX1
MEP1A
LYZ
LTC4S
LINC−PINT
LGALS4
LAMC3
KRT86
KRT20
KCNH4
JOSD2
ISLR2
IGFBP2
IGF1
HYAL1
HLA−DRB5
HLA−DRB1
HCG26
HCFC1R1
GPX2
GABRP
FXYD2
F8A1
EEF1A2
DNAJB13
DCDC2
CRIP1
CHGA
CCDC152
C22orf15
C2
APOL3
APOE
ALDH2
ADAMTS8
Apoptosis_A
Apoptosis_I
CellCycle_A
CellCycle_I
DNADamage_A
DNADamage_I
EMT_A
EMT_I
Hormone AR_A
Hormone AR_I
Hormone ER_A
Hormone ER_I
PI3KAKT_A
PI3KAKT_I
RASMAPK_A
RASMAPK_I
RTK_A
RTK_I
TSCmTOR_A
TSCmTOR_I
Pathway (A: Activate; I: Inhibit)
Percent
100
Inhibit
0
100
Activate
BLCA
Cancer related pathway
Apoptosis
CellCycle
DNADamage
EMT
Hormone AR
Hormone ER
PI3KAKT
RASMAPK
RTK
TSCmTOR
Spearman cor.
0.1
0.0
−0.1
−0.2
−0.3

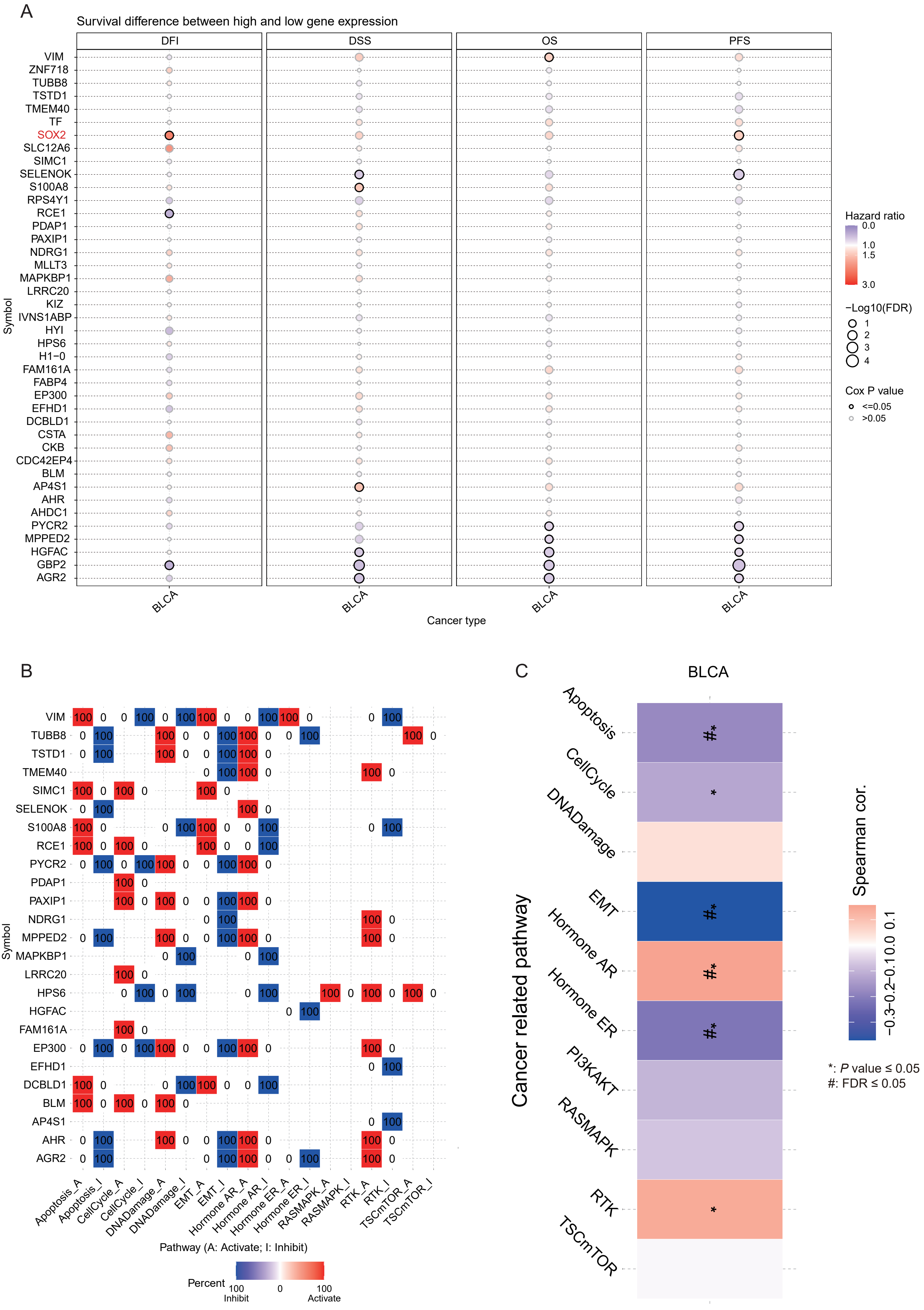
A
Survival difference between high and low gene expression
DFI
DSS
OS
PFS
VIM
ZNF718
TUBB8
TSTD1
TMEM40
TF
SOX2
SLC12A6
SIMC1
SELENOK
S100A8
RPS4Y1
RCE1
PDAP1
PAXIP1
NDRG1
MLLT3
MAPKBP1
LRRC20
KIZ
IVNS1ABP
HYI
HPS6
H1−0
FAM161A
FABP4
EP300
EFHD1
DCBLD1
CSTA
CKB
CDC42EP4
BLM
AP4S1
AHR
AHDC1
PYCR2
MPPED2
HGFAC
GBP2
AGR2
Symbol
BLCA
Cancer type
Hazard ratio
0.0
1.0
1.5
3.0
−Log10(FDR)
1
2
3
4
Cox P value
<=0.05
>0.05
B
Symbol
Apoptosis_A
Apoptosis_I
CellCycle_A
CellCycle_I
DNADamage_A
DNADamage_I
EMT_A
EMT_I
Hormone AR_A
Hormone AR_I
Hormone ER_A
Hormone ER_I
RASMAPK_A
RASMAPK_I
RTK_A
RTK_I
TSCmTOR_A
TSCmTOR_I
Pathway (A: Activate; I: Inhibit)
Percent
100
Inhibit
0
100
Activate
C
BLCA
Apoptosis
CellCycle
DNADamage
EMT
Hormone AR
Hormone ER
PI3KAKT
RASMAPK
RTK
TSCmTOR
Cancer related pathway
Spearman cor.
−0.3
−0.2
−0.1
0.0
0.1
*: P value ≤ 0.05
#: FDR ≤ 0.05

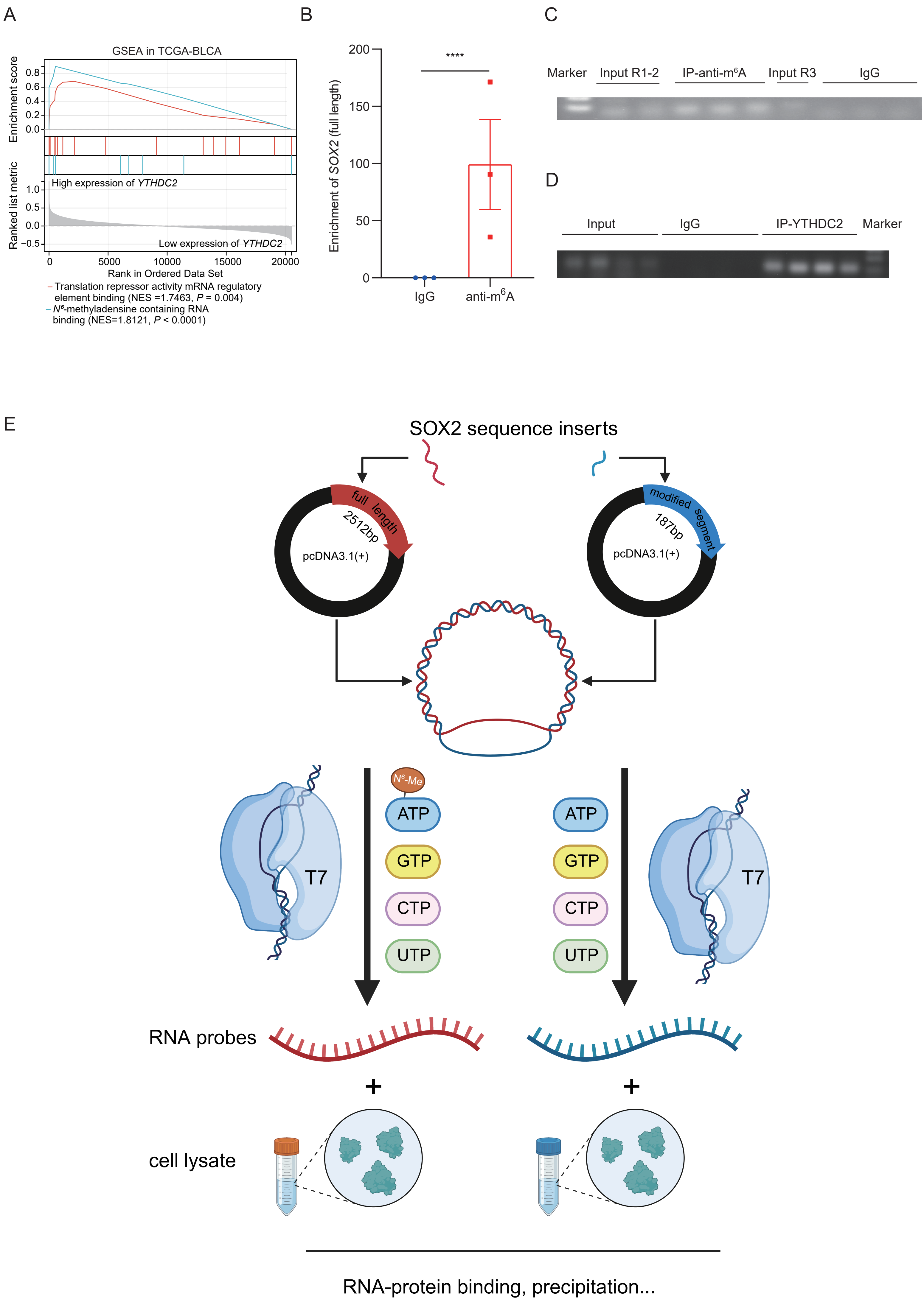

A
GSEA in TCGA-BLCA
Enrichment score
Ranked list metric
High expression of YTHDC2
Low expression of YTHDC2
Rank in Ordered Data Set
– Translation repressor activity mRNA regulatory element binding (NES =1.7463, P = 0.004)
– N6-methyladensine containing RNA binding (NES=1.8121, P < 0.0001)
B
Enrichment of SOX2 (full length)
****
IgG
anti-m6A
C
Marker
Input R1-2
IP-anti-m6A
Input R3
IgG
D
Input
IgG
IP-YTHDC2
Marker
E
SOX2 sequence inserts
full length
2512bp
pcDNA3.1(+)
modified segment
187bp
pcDNA3.1(+)
N6-Me
ATP
GTP
CTP
UTP
T7
RNA probes
cell lysate
RNA-protein binding, precipitation...

A

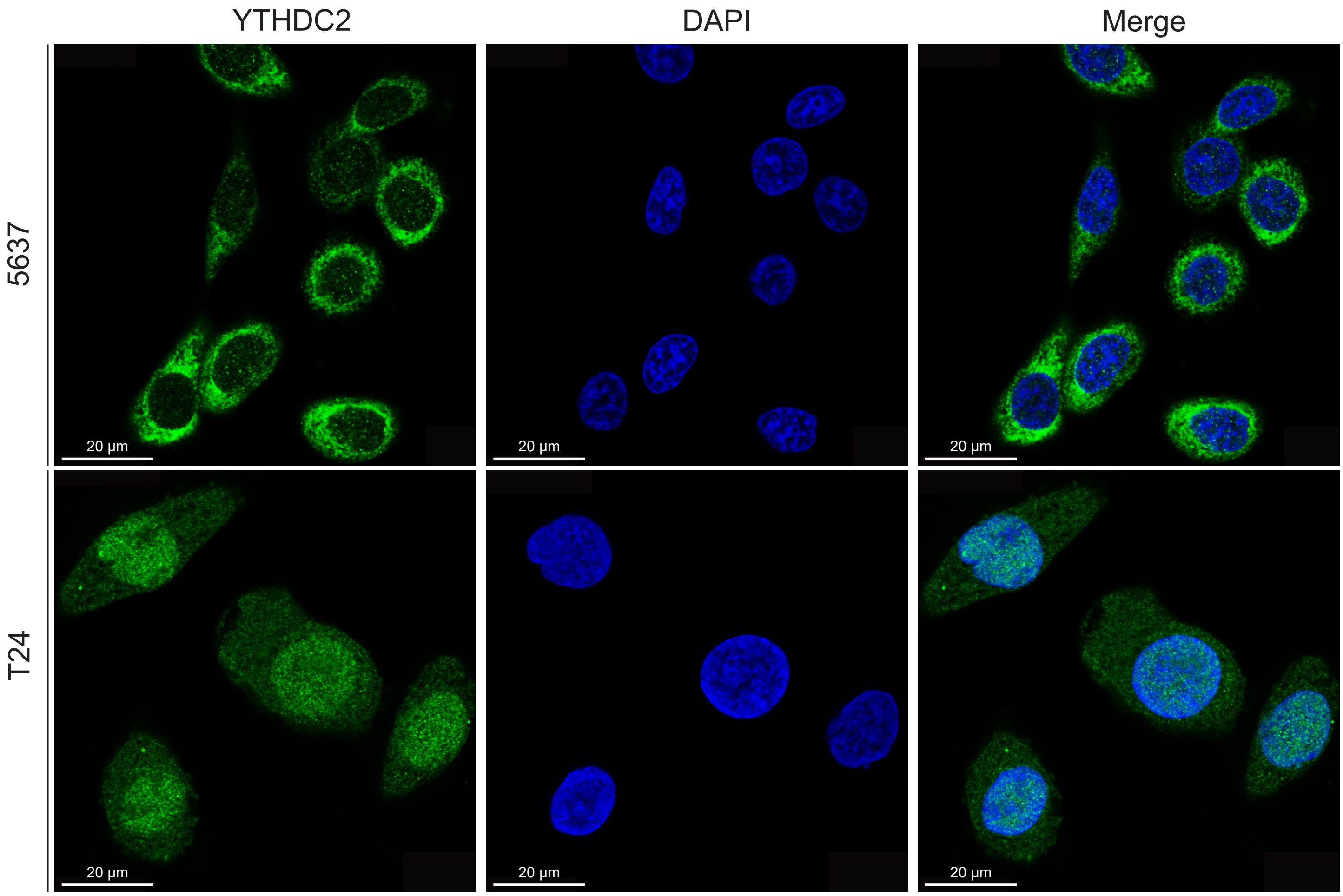

Supplement: Supplementary file 3 — Supplementary figures [file 41419_2025_8079_MOESM3_ESM.pdf]
