# Supplementary material for: YTHDC2 suppresses bladder cancer by inhibiting SOX2-mediated tumor plasticity
Source: Cell Death Dis. 2025 Oct 27;16(1):765. doi: 10.1038/s41419-025-08079-w (PMC12559364; doi:10.1038/s41419-025-08079-w)
Supplement: Supplementary file 4 — Table. S1. oligonucleotide and sequences inserted in plasmid [file 41419_2025_8079_MOESM4_ESM.pdf]

**Table S1.**		
**Name**	**Forward**	**Reverse**
**Primer sequences for qRT-PCR**		
YTHDC2	CAAGACAAGTGGGCGACTCA	AGGAACTGAGGAATCTGTGTGG
SOX2	ACATGAACGGCTGGAGCAA	GTAGGACATGCTGTAGGTGGG
GAPDH	CCATGGAGAAGGCTGGGG	CAAAGTTGTCATGGATGACC
**Primer sequences for RIP**		
YTHDC2	CAAGACAAGTGGGCGACTCA	AGGAACTGAGGAATCTGTGTGG
SOX2	ACATGAACGGCTGGAGCAA	GTAGGACATGCTGTAGGTGGG
**Primer sequences for MeRIP**		
SOX2	ACATGAACGGCTGGAGCAA	GTAGGACATGCTGTAGGTGGG
SOX2 m6A modified section	AATAGCATGGCGAGCGGG	GTACTGCAGGGCGCTCAC
**Gene Sequences use in palsmid design for RNA pull-down assays**		
SOX2 FL	GATGGTTGTCTATTAACTTGTTCAAAAAAGTATCAGGAGTTGTCAAGGCAGAGAAGAGAGTGTTTGCAAAAGGGGGAAAGTAGTTTGCTGCCTCTTTAAGACT<br>AGGACTGAGAGAAAGAAGAGGAGAGAGAAAGAAAGGGAGAGAAGTTTGAGCCCCAGGCTTAAGCCTTTCCAAAAAATAATAATAACAATCATCGGCGGCGGC<br>AGGATCGGCCAGAGGAGGAGGGAAGCGCTTTTTTTGATCCTGATTCCAGTTTGCCTCTCTCTTTTTTTCCCCCAAATTATTCTTCGCCTGATTTTCCTCGCGGAG<br>CCCTGCGCTCCCGACACCCCCGCCCGCCTCCCCTCCTCCTCTCCCCCCGCCCGCGGGCCCCCCAAAGTCCCGGCCGGGCCGAGGGTCGGCGGCCGCCGGCGGGC<br>CGGGCCCGCGCACAGCGCCCGCATGTACAACATGATGGAGACGGAGCTGAAGCCGCCGGGCCCGCAGCAAACTTCGGGGGGCGGCGGCGGCAACTCCACCGC<br>GGCGGCGGCCGGCGGCAACCAGAAAAACAGCCCGGACCGCGTCAAGCGGCCCATGAATGCCTTCATGGTGTGGTCCCGCGGGCAGCGGCGCAAGATGGCCCA<br>GGAGAACCCCAAGATGCACAACTCGGAGATCAGCAAGCGCCTGGGCGCCGAGTGGAAACTTTTGTCGGAGACGGAGAAGCGGCCGTTCATCGACGAGGCTAA<br>GCGGCTGCGAGCGCTGCACATGAAGGAGCACCCGGATTATAAATACCGGCCCCGGCGGAAAACCAAGACGCTCATGAAGAAGGATAAGTACACGCTGCCCGG<br>CGGGCTGCTGGCCCCCGGCGGCAATAGCATGGCGAGCGGGGTCGGGGTGGGCGCCGGCCTGGGCGCGGGCGTGAACCAGCGCATGGACAGTTACGCGCACAT<br>GAACGGCTGGAGCAACGGCAGCTACAGCATGATGCAGGACCAGCTGGGCTACCCGCAGCACCCGGGCCTCAATGCGCACGGCGCAGCGCAGATGCAGCCCAT<br>GCACCGCTACGACGTGAGCGCCCTGCAGTACAACTCCATGACCAGCTCGCAGACCTACATGAACGGCTCGCCCACCTACAGCATGTCCTACTCGCAGCAGGGC<br>ACCCCTGGCATGGCTCTTGGCTCCATGGGTTCGGTGGTCAAGTCCGAGGCCAGCTCCAGCCCCCCTGTGGTTACCTCTTCCTCCCACTCCAGGGCGCCCTGCCA<br>GGCCGGGGACCTCCGGGACATGATCAGCATGTATCTCCCCGGCGCCGAGGTGCCGGAACCCGCCGCCCCCAGCAGACTTCACATGTCCCAGCACTACCAGAGC<br>GGCCCGGTGCCCGGCACGGCCATTAACGGCACACTGCCCCTCTCACACATGTGAGGGCCGGACAGCGAACTGGAGGGGGGAGAAATTTTCAAAGAAAAACGA<br>GGGAAATGGGAGGGGTGCAAAAGAGGAGAGTAAGAAACAGCATGGAGAAAACCCGGTACGCTCAAAAAGAAAAAGGAAAAAAAAAAATCCCATCACCCACA<br>GCAAATGACAGCTGCAAAAGAGAACACCAATCCCATCCACACTCACGCAAAAACCGCGATGCCGACAAGAAAACTTTTATGAGAGAGATCCTGGACTTCTTTT<br>TGGGGGACTATTTTTGTACAGAGAAAACCTGGGGAGGGTGGGGAGGGCGGGGGAATGGACCTTGTATAGATCTGGAGGAAAGAAAGCTACGAAAAACTTTTT<br>AAAAGTTCTAGTGGTACGGTAGGAGCTTTGCAGGAAGTTTGCAAAAGTCTTTACCAATAATATTTAGAGCTAGTCTCCAAGCGACGAAAAAAATGTTTTAATAT<br>TTGCAAGCAACTTTTGTACAGTATTTATCGAGATAAACATGGCAATCAAAATGTCCATTGTTTATAAGCTGAGAATTTGCCAATATTTTTCAAGGAGAGGCTTCT<br>TGCTGAATTTTGATTCTGCAGCTGAAATTTAGGACAGTTGCAAACGTGAAAAGAAGAAAATTATTCAAATTTGGACATTTTAATTGTTTAAAAATTGTACAAAA<br>GGAAAAAATTAGAATAAGTACTGGCGAACCATCTCTGTGGTCTTGTTTAAAAAGGGCAAAAGTTTTAGACTGTACTAAATTTTATAACTTACTGTTAAAAGCAA<br>AAATGGCCATGCAGGTTGACACCGTTGGTAATTTATAATAGCTTTTGTTCGATCCCAACTTTCCATTTTGTTCAGATAAAAAAAACCATGAAATTACTGTGTTTG<br>AAATATTTTCTTATGGTTTGTAATATTTCTGTAAATTTATTGTGATATTTTAAGGTTTTCCCCCCTTTATTTTCCGTAGTTGTATTTTAAAAGATTCGGCTCTGTA<br>TTATTTGAATCAGTCTGCCGAGAATCCATGTATATATTTGAACTAATATCATCCTTATAACAGGTACATTTTCAACTTAAGTTTTTACTCCATTATGCACAGTTTG<br>AGATAAATAAATTTTTGAAATATGGACACTGAAA	
SOX2 segment	GGGGTGGGCGCCGGCCTGGGCGCGGGCGTGAACCAGCGCATGGACAGTTACGCGCACATGAACGGCTGGAGCAACGGCAGCTACAGCATGATGCAGGACCAG<br>CTGGGCTACCCGCAGCACCCGGGCCTCAATGCGCACGGCGCAGCGCAGATGCAGCCCATGCACCGCTACGACGTGAGCGCCCTGC	
**Gene Sequences use in palsmid design for Dual Luciferase Reporter assays**		
SOX2-FL-WT	GATGGTTGTCTATTAACTTGTTCAAAAAAGTATCAGGAGTTGTCAAGGCAGAGAAGAGAGTGTTTGCAAAAGGGGGAAAGTAGTTTGCTGCCTCTTTAAGACT<br>AGGACTGAGAGAAAGAAGAGGAGAGAGAAAGAAAGGGAGAGAAGTTTGAGCCCCAGGCTTAAGCCTTTCCAAAAAATAATAATAACAATCATCGGCGGCGGC<br>AGGATCGGCCAGAGGAGGAGGGAAGCGCTTTTTTTGATCCTGATTCCAGTTTGCCTCTCTCTTTTTTTCCCCCAAATTATTCTTCGCCTGATTTTCCTCGCGGAG<br>CCCTGCGCTCCCGACACCCCCGCCCGCCTCCCCTCCTCCTCTCCCCCCGCCCGCGGGCCCCCCAAAGTCCCGGCCGGGCCGAGGGTCGGCGGCCGCCGGCGGGC<br>CGGGCCCGCGCACAGCGCCCGCATGTACAACATGATGGAGACGGAGCTGAAGCCGCCGGGCCCGCAGCAAACTTCGGGGGGCGGCGGCGGCAACTCCACCGC<br>GGCGGCGGCCGGCGGCAACCAGAAAAACAGCCCGGACCGCGTCAAGCGGCCCATGAATGCCTTCATGGTGTGGTCCCGCGGGCAGCGGCGCAAGATGGCCCA<br>GGAGAACCCCAAGATGCACAACTCGGAGATCAGCAAGCGCCTGGGCGCCGAGTGGAAACTTTTGTCGGAGACGGAGAAGCGGCCGTTCATCGACGAGGCTAA<br>GCGGCTGCGAGCGCTGCACATGAAGGAGCACCCGGATTATAAATACCGGCCCCGGCGGAAAACCAAGACGCTCATGAAGAAGGATAAGTACACGCTGCCCGG<br>CGGGCTGCTGGCCCCCGGCGGCAATAGCATGGCGAGCGGGGTCGGGGTGGGCGCCGGCCTGGGCGCGGGCGTGAACCAGCGCATGGACAGTTACGCGCACAT<br>GAACGGCTGGAGCAACGGCAGCTACAGCATGATGCAGGACCAGCTGGGCTACCCGCAGCACCCGGGCCTCAATGCGCACGGCGCAGCGCAGATGCAGCCCAT<br>GCACCGCTACGACGTGAGCGCCCTGCAGTACAACTCCATGACCAGCTCGCAGACCTACATGAACGGCTCGCCCACCTACAGCATGTCCTACTCGCAGCAGGGC<br>ACCCCTGGCATGGCTCTTGGCTCCATGGGTTCGGTGGTCAAGTCCGAGGCCAGCTCCAGCCCCCCTGTGGTTACCTCTTCCTCCCACTCCAGGGCGCCCTGCCA<br>GGCCGGGGACCTCCGGGACATGATCAGCATGTATCTCCCCGGCGCCGAGGTGCCGGAACCCGCCGCCCCCAGCAGACTTCACATGTCCCAGCACTACCAGAGC<br>GGCCCGGTGCCCGGCACGGCCATTAACGGCACACTGCCCCTCTCACACATGTGAGGGCCGGACAGCGAACTGGAGGGGGGAGAAATTTTCAAAGAAAAACGA<br>GGGAAATGGGAGGGGTGCAAAAGAGGAGAGTAAGAAACAGCATGGAGAAAACCCGGTACGCTCAAAAAGAAAAAGGAAAAAAAAAAATCCCATCACCCACA<br>GCAAATGACAGCTGCAAAAGAGAACACCAATCCCATCCACACTCACGCAAAAACCGCGATGCCGACAAGAAAACTTTTATGAGAGAGATCCTGGACTTCTTTT<br>TGGGGGACTATTTTTGTACAGAGAAAACCTGGGGAGGGTGGGGAGGGCGGGGGAATGGACCTTGTATAGATCTGGAGGAAAGAAAGCTACGAAAAACTTTTT<br>AAAAGTTCTAGTGGTACGGTAGGAGCTTTGCAGGAAGTTTGCAAAAGTCTTTACCAATAATATTTAGAGCTAGTCTCCAAGCGACGAAAAAAATGTTTTAATAT<br>TTGCAAGCAACTTTTGTACAGTATTTATCGAGATAAACATGGCAATCAAAATGTCCATTGTTTATAAGCTGAGAATTTGCCAATATTTTTCAAGGAGAGGCTTCT<br>TGCTGAATTTTGATTCTGCAGCTGAAATTTAGGACAGTTGCAAACGTGAAAAGAAGAAAATTATTCAAATTTGGACATTTTAATTGTTTAAAAATTGTACAAAA<br>GGAAAAAATTAGAATAAGTACTGGCGAACCATCTCTGTGGTCTTGTTTAAAAAGGGCAAAAGTTTTAGACTGTACTAAATTTTATAACTTACTGTTAAAAGCAA<br>AAATGGCCATGCAGGTTGACACCGTTGGTAATTTATAATAGCTTTTGTTCGATCCCAACTTTCCATTTTGTTCAGATAAAAAAAACCATGAAATTACTGTGTTTG<br>AAATATTTTCTTATGGTTTGTAATATTTCTGTAAATTTATTGTGATATTTTAAGGTTTTCCCCCCTTTATTTTCCGTAGTTGTATTTTAAAAGATTCGGCTCTGTA<br>TTATTTGAATCAGTCTGCCGAGAATCCATGTATATATTTGAACTAATATCATCCTTATAACAGGTACATTTTCAACTTAAGTTTTTACTCCATTATGCACAGTTTG<br>AGATAAATAAATTTTTGAAATATGGACACTGAAA	

SOX2-FL-MT1/2/3	GATGGTTGTCTATTAACTTGTTCAAAAAAGTATCAGGAGTTGTCAAGGCAGAGAAGAGAGTGTTTGCAAAAGGGGGAAAGTAGTTTGCTGCCTCTTTAAGACT<br>AGGACTGAGAGAAAGAAGAGGAGAGAGAAAGAAAGGGAGAGAAGTTTGAGCCCCAGGCTTAAGCCTTTCCAAAAAATAATAATAACAATCATCGGCGGCGGC<br>AGGATCGGCCAGAGGAGGAGGGAAGCGCTTTTTTTGATCCTGATTCCAGTTTGCCTCTCTCTTTTTTTCCCCCAAATTATTCTTCGCCTGATTTCCTCGCGGAG<br>CCCTGCGCTCCCGACACCCCCGCCCGCCTCCCCTCCTCCTCTCCCCCCGCCCGCGGGCCCCCCAAAGTCCCGGCCGGGCCGAGGGTCGGCGGCCGCCGGCGGGC<br>CGGGCCCGCGCACAGCGCCCGCATGTACAACATGATGGAGACGGAGCTGAAGCCGCCGGGCCCGCAGCAAACTTCGGGGGGCGGCGGCGGCAACTCCACCGC<br>GGCGGCGGCCGGCGGCAACCAGAAAAACAGCCCGGACCGCGTCAAGCGGCCCATGAATGCCTTCATGGTGTGGTCCCGCGGGCAGCGGCGCAAGATGGCCCA<br>GGAGAACCCCAAGATGCACAACTCGGAGATCAGCAAGCGCCTGGGCGCCGAGTGGAAACTTTTGTCGGAGACGGAGAAGCGGCCGTTCATCGACGAGGCTAA<br>GCGGCTGCGAGCGCTGCACATGAAGGAGCACCCGGATTATAAATACCGGCCCCGGCGGAAAACCAAGACGCTCATGAAGAAGGATAAGTACACGCTGCCCGG<br>CGGGCTGCTGGCCCCCGGCGGCAATAGCATGGCGAGCGGGGTCGGGGTGGGCGCCGGCCTGGGCGCGGGCGTGACCCAGCGCATGGCCAGTTACGCGCACAT<br>GAACGGCTGGAGCAACGGCAGCTACAGCATGATGCAGGCCCAGCTGGGCTACCCGCAGCACCCGGGCCTCAATGCGCACGGCGCAGCGCAGATGCAGCCCAT<br>GCACCGCTACGACGTGAGCGCCCTGCAGTACAACTCCATGACCAGCTCGCAGACCTACATGAACGGCTCGCCCACCTACAGCATGTCCTACTCGCAGCAGGGC<br>ACCCCTGGCATGGCTCTTGGCTCCATGGGTTCGGTGGTCAAGTCCGAGGCCAGCTCCAGCCCCCCTGTGGTTACCTCTTCCTCCCACTCCAGGGCGCCCTGCCA<br>GGCCGGGGACCTCCGGGACATGATCAGCATGTATCTCCCCGGCGCCGAGGTGCCGGAACCCGCCGCCCCCAGCAGACTTCACATGTCCCAGCACTACCAGAGC<br>GGCCCGGTGCCCGGCACGGCCATTAACGGCACACTGCCCCTCTCACACATGTGAGGGCCGGACAGCGAACTGGAGGGGGGAGAAATTTTCAAAGAAAAACGA<br>GGGAAATGGGAGGGGTGCAAAAGAGGAGAGTAAGAAACAGCATGGAGAAAACCCGGTACGCTCAAAAAGAAAAAGGAAAAAAAAAAAATCCCATCACCCACA<br>GCAAATGACAGCTGCAAAAGAGAACACCAATCCCATCCACACTCACGCAAAAACCGCGATGCCGACAAGAAAACTTTTATGAGAGAGATCCTGGACTTCTTTT<br>TGGGGGACTATTTTTGTACAGAGAAAACCTGGGGAGGGTGGGGAGGGCGGGGGAATGGACCTTGTATAGATCTGGAGGAAAGAAAGCTACGAAAAACTTTTT<br>AAAAGTTCTAGTGGTACGGTAGGAGCTTTGCAGGAAGTTTGCAAAAGTCTTTACCAATAATATTTAGAGCTAGTCTCCAAGCGACGAAAAAAATGTTTTAATAT<br>TTGCAAGCAACTTTTGTACAGTATTTATCGAGATAAACATGGCAATCAAAATGTCCATTGTTTATAAGCTGAGAATTTGCCAATATTTTTCAAGGAGAGGCTTCT<br>TGCTGAATTTTGATTCTGCAGCTGAAATTTAGGACAGTTGCAAACGTGAAAAGAAGAAAATTATTCAAATTTGGACATTTTAATTGTTTAAAAATTGTACAAAA<br>GGAAAAAATTAGAATAAGTACTGGCGAACCATCTCTGTGGTCTTGTTTAAAAAGGGCAAAAGTTTTAGACTGTACTAAATTTTATAACTTACTGTTAAAAGCAA<br>AAATGGCCATGCAGGTTGACACCGTTGGTAATTTATAATAGCTTTTGTTCGATCCCAACTTTCCATTTTGTTCAGATAAAAAAAACCATGAAATTACTGTGTTTG<br>AAATATTTTCTTATGGTTTGTAATATTTCTGTAAATTTATTGTGATATTTTAAGGTTTTCCCCCCTTTATTTTCCGTAGTTGTATTTTAAAAGATTCGGCTCTGTA<br>TTATTTGAATCAGTCTGCCGAGAATCCATGTATATATTTGAACTAATATCATCCTTATAACAGGTACATTTTCAACTTAAGTTTTTACTCCATTATGCACAGTTTG<br>AGATAAATAAATTTTTGAAATATGGACACTGAAA
SOX2-WT	GGGGTGGGCGCCGGCCTGGGCGCGGGCGTGAACCAGCGCATGGACAGTTACGCGCACATGAACGGCTGGAGCAACGGCAGCTACAGCATGATGCAGGACCAG<br>CTGGGCTACCCGCAGCACCCGGGCCTCAATGCGCACGGCGCAGCGCAGATGCAGCCCATGCACCGCTACGACGTGAGCGCCCTGC
SOX2-MT1/2/3	GGGGTGGGCGCCGGCCTGGGCGCGGGCGTGACCCAGCGCATGGCCAGTTACGCGCACATGAACGGCTGGAGCAACGGCAGCTACAGCATGATGCAGGCCCAG<br>CTGGGCTACCCGCAGCACCCGGGCCTCAATGCGCACGGCGCAGCGCAGATGCAGCCCATGCACCGCTACGACGTGAGCGCCCTGC
