# Supplementary material for: YTHDC2 suppresses bladder cancer by inhibiting SOX2-mediated tumor plasticity
Source: Cell Death Dis. 2025 Oct 27;16(1):765. doi: 10.1038/s41419-025-08079-w (PMC12559364; doi:10.1038/s41419-025-08079-w)
Supplement: Supplementary file 5 — Table. S2. m6A motifs prediction [file 41419_2025_8079_MOESM5_ESM.pdf]

Modification Type	Chromosome Position	Source	Species	Gene	Variant(s)	SNP Position	Database	Transcriptome Regulation	Disease	Detail
m6A	chr3:181712821(+)	MeRIP-seq (Medium)	Human	SOX2,SOX2-OT	rs750665327	chr3:181712822..181712822	dbSNP153	circRNA 4		Detail
m6A	chr3:181712821(+)	MeRIP-seq (Medium)	Human	SOX2,SOX2-OT	rs104893803	chr3:181712823..181712823	dbSNP153	circRNA 4	Anophthalmia/microphthalmia-esophageal atresia syndrome;	Detail
m6A	chr3:181712833(+)	miCLIP (High)	Human	SOX2,SOX2-OT	rs959093909	chr3:181712833..181712833	dbSNP153	circRNA 4		Detail
m6A	chr3:181712833(+)	miCLIP (High)	Human	SOX2,SOX2-OT	rs1247718049	chr3:181712835..181712835	dbSNP153	circRNA 4		Detail
m6A	chr3:181712821(+)	MeRIP-seq (Medium)	Human	SOX2,SOX2-OT	rs750665327	chr3:181712822..181712822	ICGC	circRNA 4		Detail
m6A	chr3:181712887(+)	Prediction (Low)	Human	SOX2,SOX2-OT	rs104893799	chr3:181712889..181712889	dbSNP153	RBP 2<br>circRNA 4	Anophthalmia/microphthalmia-esophageal atresia syndrome;	Detail
